# Deficient AMPK-SENP1-Sirt3 signaling impairs mitochondrial complex I function in Parkinson’s disease model

**DOI:** 10.1186/s40035-025-00489-2

**Published:** 2025-07-01

**Authors:** Xiaoyu Sun, Jianyi Shen, Yimei Shu, Tianshi Wang, Lu He, Ruinan Shen, Yifan Zhou, Jinke Cheng, Suzhen Lin, Jianqing Ding

**Affiliations:** 1 Department of Neurology and Institute of Neurology, Ruijin Hospital, Shanghai Jiao Tong University School of Medicine, 197 Ruijin Second Road, Shanghai, 200025 China; 2 Institute of Aging and Tissue Regeneration, Renji Hospital, Shanghai Jiao Tong University School of Medicine, 160 Pujian Road, Shanghai, 200135 China; 3 Department of Nephrology, Renji Hospital, Shanghai Jiao Tong University School of Medicine, Shanghai, China; 4 Department of Neurology, Shanghai Sixth People’s Hospital, Shanghai Jiao Tong University School of Medicine, Shanghai, China; 5 Department of Biochemistry and Molecular Cell Biology, Shanghai Key Laboratory for Tumor Microenvironment and Inflammation, Shanghai Jiao Tong University School of Medicine, Shanghai, China

**Keywords:** Parkinson's disease, SENP1, Sirt3, Mitochondrial complex I, MPTP

## Abstract

**Background:**

Epidemiological studies have revealed increased Parkinson’s disease (PD) risk among individuals exposed to pesticides like 1-methyl-4-phenyl-1,2,3,6-tetrahydropyridine (MPTP). MPTP is frequently used to induce PD-like symptoms in research models by disrupting mitochondrial complex I (CI) function and causing dopaminergic neuronal loss in the nigrostriatal region. However, the pathway(s) through which MPTP impairs mitochondrial CI function remain to be elucidated. In this study, we aim to identify the molecular mechanisms through which MPTP modulates CI function and define the specific subunits of mitochondrial CI affected by MPTP.

**Methods:**

Male mice encompassing either wild-type Sirt3 or Sirt3 K223R de-SUMOylation mutation, were intraperitoneally injected with either MPTP or saline. In vitro experiments were conducted using the SH-SY5Y cell line with or without the Sirt3 de-SUMOylation mutation. Movement performance, mitochondrial function, and protein acetylation were evaluated.

**Results:**

MPTP exposure, both in vitro and in vivo, disrupted the AMPK–SENP1–Sirt3 axis, leading to impairment of mitochondrial function. Specifically, MPTP suppressed activation of AMPK, impeding the entry of SENP1 into the mitochondria. The lack of mitochondrial SENP1 resulted in increased levels of SUMOylated Sirt3, which inhibited its deacetylase activity. This led to a significant increase in the acetylation of CI subunits NDUFS3 and NDUFA5, which resulted in reduced CI activity and inhibition of mitochondrial function, and eventually dopaminergic neuronal death. In this pathway, sustained deSUMOylation mutation of Sirt3 (K223R in mice, K288R in humans) mitigated the impact of MPTP on mitochondrial dysregulation, as well as dopaminergic neuronal death and behavioral deficits.

**Conclusion:**

The disordered AMPK-SENP1-Sirt3 pathway plays a crucial role in the MPTP-induced CI dysfunction and PD-like phenotype, which provide valuable insights into the mechanisms of PD pathogenesis.

**Supplementary Information:**

The online version contains supplementary material available at 10.1186/s40035-025-00489-2.

## Background

Parkinson's disease (PD) is a progressive neurodegenerative disorder characterized by the loss of dopaminergic neurons in the substantia nigra (SN), resulting in a variety of motor symptoms. The exact cause of PD is unknown, but a combination of genetic and environmental factors is believed to contribute to its onset. Epidemiological studies have identified a strong relationship between pesticide exposure and elevated risk of PD. These PD-related compounds include 1-methyl-4-phenyl-1,2,3,6-tetrahydropyridine (MPTP), rotenone, paraquat, and daidzein manganese [1–3], which have all been proven effective in PD model establishment [3]. Among them, MPTP stands as a well-defined PD-inducing neurotoxin, causing significant dopaminergic neuronal death at pathogenic dose administration [4, 5].

Metabolized into 1-methyl-4-phenylpyridinium ion (MPP^+^), MPTP can successfully cross the blood–brain barrier and be transferred into dopaminergic neurons [6, 7]. Existing evidence demonstrates that MPTP/MPP^+^ selectively disrupts the operation of mitochondrial complex I (CI), leading to energy depletion, oxidative stress, altered mitochondrial morphology and ultimately cellular apoptosis [4, 8–10]. Mitochondria, as the “powerhouse” of the cell, generate ATP through oxidative phosphorylation. As the first enzyme in the respiratory chain, CI oxidizes NADH produced from the Krebs cycle, and passes two electrons from the NADH to reduce ubiquinone to ubiquinol. This reaction is considered as the rate-limiting step as CI is the major gate for electrons translocation, generating the proton-motive force across inner mitochondrial membrane. Therefore, CI plays a central role in energy metabolism. CI deficiency has long been indicated in the SN of PD patients [11]. CI dysfunction alone is suggested sufficient to induce progressive, human-like parkinsonism [12].

The mitochondrial CI is composed of 45 different polypeptides, including 14 “core” subunits forming the functional enzyme which is assumed sufficient for energy production and 31 “supernumerary/accessory” subunits [13]. Studies of mitochondria from PD patients demonstrated increased oxidative damage in selective subunits ND4, ND5, NDUFS2, and NDUFV1 [14]. Moreover, Engrailed-1 enhances the expression of two critical CI subunits, NDUFS1 and NDUFS3, thereby increasing CI activity and mitigating MPTP-induced mitochondrial damage [15]. Additionally, MPTP might reduce the protein level of subunit NDUFV2 [16]. However, the exact mechanism by which MPTP induces CI malfunction is still unclear.

Reversible acetylation is a crucial modification that controls protein function. The majority of studied cases of acetylation propose that acetylation of mitochondrial enzymes is an inhibitory mark [17]. Nicotinamide adenine dinucleotide (NAD^+^)-dependent deacetylase sirtuin3 (Sirt3) is the main enzyme governing mitochondrial acetylation. Diminished Sirt3 expression has been detected in the substantia nigra pars compacta of PD subjects [18]. Sirt3 level is also reduced after MPTP treatment [19] and Sirt3 knockout dramatically exacerbates the loss of nigrostriatal dopaminergic neurons in MPTP-induced PD mice [20]. Although previous research revealed that Sirt3 plays a role in regulating acetylation of NDUFA9 [21], how Sirt3 dysfunction is involved in PD pathophysiology and the causes of its dysregulation remain largely unexplored.

Given that the precise mechanisms underlying MPTP-induced CI damage remain unclear, we employed both in vitro and in vivo models to investigate the impact of MPTP exposure on CI dysfunction and Sirt3 dysregulation.

## Methods

### Animals

Both wild-type (WT) and Sirt3 K223R mutant mice, maintained on a C57BL/6 genetic background, were used in this study. The Sirt3 K223R mice were provided by Dr. Jinke Cheng (Shanghai Jiao Tong University School of Medicine, Shanghai, China) [22]. Mice were maintained on a standard chow diet (Research Diets, D12590; New Brunswick, NJ). All procedures strictly adhered to the guidelines outlined in the Guide for the Care and Use of Laboratory Animals [23] and were approved by the Experimental Animal Ethical Committee at Shanghai Jiao Tong University School of Medicine.

### Antibodies

For Western blotting assays, the following primary antibodies were used: anti-GAPDH (1:1000; Cell Signaling, 5174; Danvers, MA), anti-ATP5A (1:300; Abcam, 110273; Cambridge, MA), anti-Sirt3 (1:1000; Cell Signaling, 5490), anti-NDUFS2 (1:4000; Abcam, 110249), anti-NDUFA5 (1:1000; Proteintech, 16640; Rosemont, IL), anti-NDUFS3 (1:2000; Abcam, 14711), anti-NDUFA9 (1:1000; Abcam, 14713), anti-NDUFV1 (1:2000; Proteintech, 11238), anti-NDUFS7 (1:2000; Proteintech, 15728), anti-SENP1 (1:1000; Abcam, 108981), anti-AMP-activated protein kinase (AMPK) (1:1000; Cell Signaling, 2532), anti-SUMO1 (1:1000, custom-made by Cheng's team) [24], anti-phosphorylated AMPK (P-AMPK) (1:1000; Cell Signaling, 2535), anti-Pan-AcK (Acetyl-conjugated proteins; 1:1000; Cell Signaling, 9441), and anti-tyrosine hydroxylase (TH)(1:200; Abcam, 112). For immunoprecipitations, anti-Sirt3 and anti-Pan-AcK were used. For immunofluorescence, chicken TH polyclonal antibody (anti-TH, Abcam, 76442; 1:1000) and ionized calcium-binding adapter molecule 1 (IBA1) (anti-IBA1, Wako, 01919741; 1:1000; Richmond, VA) were used.

Secondary antibodies used in this study included Goat Anti-Rabbit IgG (H + L) Antibody (1:10,000; Cell Signaling, 35401), Goat Anti-Mouse IgG (H + L) Antibody (1:10,000; Abcam, 205719) and Mouse Anti-Rabbit IgG (Conformation Specific) (L27A9) mAb (1:3000; Cell Signaling, 5127); as well as Goat Anti-Rabbit IgG H&L (Alexa Fluor® 488) (1:1000; Abcam, 150077) and Goat Anti-Chicken IgY H&L (Alexa Fluor® 594) (1:1000; Abcam, 150172) for immunofluorescence.

### MPTP treatment

A cohort of 3-month-old male Sirt3 K223R mice and their WT littermates were intraperitoneally injected with either MPTP/HCl (30 mg/kg of body weight, Sigma, M0896; St. Louis, MO) or an equivalent volume of 0.9% saline for 5 consecutive days. The volume of MPTP injected in each mouse was adjusted using a correction factor based on the body weight of each animal. The MPTP solution was prepared at a concentration of 3 mg/mL, and the injection volume was calculated to ensure a consistent dose of 30 mg/kg for each mouse. This approach allowed us to standardize the MPTP exposure across all animals, accounting for variations in body weight.

### Animal behavioral test

The rotarod [25–27], wire hanging [28], swimming [29, 30], open field [31], and pole tests [32, 33] can effectively evaluate motor impairments in PD models. The selection of tests can differ among different research teams [34]. We adopted four distinct behavioral tests, including open field test, rotarod test, pole test and wire hanging test in this study. Behavioral tests were performed by researchers blinded to experimental conditions. Animal coding, testing, and data analysis were conducted by separate persons to maintain blinding until completion of preliminary analyses.

#### Open field test

The open field test was conducted using a plexiglass box measuring 50 cm × 50 cm × 15 cm, from which a 6 cm × 6 cm grid was cut at the bottom. Each mouse was placed in the box, in a quiet and dimly lit environment. Following a 10-min adaptation period, the movement of the mouse was recorded for 5 min, including the distance traveled and the time spent in motion. This test was repeated 5 times, with intervals ranging from 5 to 30 min.

#### Rotarod test

The rotarod test used a 6-cm diameter roller rotating at 15 rounds/min. After 5 adaptations, test was performed. The duration of mouse remaining balanced and mobile on the roller was measured. The test was repeated every 5–30 min for an average of 5 consecutive times.

#### Pole test

The pole test used a straight wooden pole 0.8 cm in diameter and 60 cm high, with a small wooden ball at the top, covered with gauze to prevent mice from slipping. Each mouse was positioned at the pole's summit, facing upwards [35]. The time from initiation of movement until both front paws touching the ground at the bottom of the pole was recorded. The test was repeated every 5–30 min for an average of 5 consecutive times.

#### Wire hanging test

For the wire hanging test, a horizontal metal rod (approximately 1.5 mm in diameter) was fixed 30 cm above the ground. The top of the metal rod was covered with a 1-cm wooden layer to prevent mice from flipping over and sitting on it. During the experiment, the mice were suspended from the metal pole, and the time taken to reach the ground was recorded. The test was repeated every 5–30 min for an average of 5 consecutive times.

### Immunofluorescence

Immunofluorescence staining was performed as previously described [36]. Briefly, mice were deeply anesthetized via intraperitoneal injection of sodium pentobarbital (50 mg/kg), confirmed by the absence of a pedal withdrawal reflex (toe pinch), and then euthanized by transcardiac perfusion with physiological saline followed by 4% paraformaldehyde (PFA) in phosphate buffer. Brains were quickly obtained and post-fixed in 4% PFA for 24–48 h. After fixation, the brains were placed in 15%, 22.5% and 30% sucrose solutions. Midbrain regions containing SN and corpus striatum were dissected from the whole brain and were frozen in optimal cutting temperature encapsulation medium (Sakura Finetek, 4583; Torrance, CA). Coronal sections (25-μm thick) were prepared using a cryostat (Leica, CM1950; Wetzlar, Germany). The SN was delineated from 2.9 to 4.1 mm posterior to the bregma [37]. The corpus striatum was delineated from 1.7 mm anterior to 1.5 mm posterior to the bregma. These sections were then blocked with 5% BSA for 1 h and incubated with primary antibody (anti-TH or anti-IBA1) overnight at 4 °C (12–16 h). Subsequently, sections were incubated with appropriate secondary antibodies and mounted on gelatin-coated slides covered in fade-resistant mounting medium with DAPI. Microscopic confocal imaging was performed using a Leica TCP SP8 (Wetzlar, Germany) laser scanning confocal microscope.

### Reactive oxygen species (ROS) assessment

Dihydroethidium (DHE, Sigma, 37291) staining was performed to evaluate the generation of ROS, following the manufacturer's protocol [38]. Briefly, brain slices containing the SN region were incubated with DHE (100 μmol/L) in the dark for 90 min at 37 °C and subsequently observed under a Leica TCS SP8 confocal laser scanning microscope (Leica Microsystems; Wetzlar, Germany).

The ROS levels were also detected using 8-hydroxy-2′-deoxyguanosine (8-OHdG, Abcam, 48508; 1:100). Brain sections were incubated with 8-OHdG for 12–16 h at 4 °C [39], followed by incubation with biotinylated secondary antibody for 60 min at room temperature. Labeling was visualized with 3,3-diaminobenzidine. Quantitative analysis was performed using the Fiji software (http://fiji.sc; software version 2.0.0-rc-68/1.52e) including plugins such as analyze particles, intensity measurements, ROI Manager, morphoLibJ, shape descriptors and cellProfiler.

### Mitochondrial electron microscopy

Brain samples were collected and fixed as that for immunofluorescence [36]. Approximately 1-mm thick sections containing the SN region were prepared and embedded in Epon resin (Sigma, 31185). Ultra-thin sections (50–70 nm) were cut using an ultramicrotome, stained with 2% acidified uranyl acetate (Electron Microscopy Sciences; Hatfield, PA), and then subjected to Sato’s triple lead staining. The sections were subsequently examined using an FEI Tecnai T12 electron microscope (FEI Company; Hillsboro, OR) [37].

### Cell culture

SH-SY5Y cells were cultured in Dulbecco's Modified Eagle Medium (DMEM; Gibco BRL, 11965126; Grand Island, NY) supplemented with 10% fetal bovine serum (Gibco BRL, 10099158), 50 U/mL penicillin, and 50 μg/mL streptomycin sulfate (Gibco BRL, 15140122). Cells were incubated at 37 °C in a humidified atmosphere with 5% CO_2_. The Sirt3 K288R SH-SY5Y cell line and its WT counterpart were obtained from Hanbio company (Shanghai, China) and maintained under the same culture conditions as the SH-SY5Y cells.

### Cell viability assay

To evaluate viability, cells were cultured at a density of 5000 cells in 96-well plates containing glucose medium. Next, 10 μL of Cell Counting Kit-8 (CCK-8, Beyotime, C0043; Shanghai, China) solution was added to each well and incubated for 1 h [40]. Absorbance was then measured at 450 nm using a microplate reader (Biotek Instruments; Winooski, VT).

### Mitochondria isolation

Mitochondria were isolated from the ventral midbrain tissues or cultured cells according to a previously reported protocol [41]. Specifically, samples were homogenized using a glass-Teflon motorized homogenizer for 60 cycles in an ice-cold isolation buffer (MS buffer) containing 210 mmol/L mannitol, 70 mmol/L sucrose, 5 mmol/L Tris–HCl (pH 7.5), 1 mmol/L EGTA, 0.5 mg/mL BSA, and protease inhibitor. The homogenates were first centrifuged at 1000× *g* for 10 min, and the supernatants were subsequently centrifuged at 3500× *g* for 10 min. The resulting pellets were re-suspended in MS buffer, centrifuged again at 1000× *g* for 5 min, and the supernatant was centrifuged at 3500× *g* for 5 min. The final pellets, representing purified mitochondria, were used in subsequent experiments.

### Immunoprecipitations

For immunoprecipitation, isolated mitochondria were lysed in NP40 lysis buffer (Beyotime, P0013F) and centrifuged at 14,000× *g* for 10 min. Total protein concentrations in the supernatant were quantified using a BCA Kit (Thermo, A55860; Waltham, MA), and 1 mg of total protein was mixed with 8 μg of antibody (anti-Sirt3 or anti-Pan-AcK) and incubated at 4 °C overnight (12–16 h). Protein G beads were then added and incubated at room temperature for 20–30 min to capture the antibody-antigen complex. Beads were washed three to five times with IP washing buffer, and proteins were eluted by boiling the beads in 1× loading buffer for 5 min. The eluted proteins were visualized by immunoblotting using the respective antibodies.

### Sirt3 activity measurement

Sirt3 activity was assessed using the Sirt3 Activity Assay Kit (Abcam, ab156067) following the manufacturer’s protocol. Fluorescence intensity was measured at 30 min using a microtiter plate fluorometer, with excitation at 340–360 nm and emission at 440–460 nm.

### Oxygen consumption rate (OCR)

To measure intracellular OCR, 10,000 cells were plated in an XF96-well microplate (see Fig. 4j legend for cell type details) and analyzed at 37 °C using the Seahorse XF Cell Mito Stress Test Kit (Seahorse, Bioscience/Agilent Technologies, 103015-100; North Billerica, MA) following the manufacturer's instructions [42]. Briefly, 2 mmol/L oligomycin, 0.25 mmol/L trifluoromethoxy carbonylcyanide phenylhydrazone (FCCP), and 0.5 mmol/L rotenone/antimycin were sequentially added to measure basal respiration, ATP production, maximal respiration and minimal respiration, respectively, using an XF96 Analyzer (Seahorse).

For extracellular OCR measurement, 10,000 cells were seeded in 96-well plates. Each well was added with 10 μL of extracellular O_2_ consumption reagent (Abcam, 197243) and 5 μL of the test compound, followed by sealing with 100 μL of pre-warmed mineral oil. The O_2_ consumption signal was measured every 1.5 min for 90 min at Ex/Em = 380/650 nm [43].

### CI activity assay, ATP measurement and JC10 staining

The activity of mitochondrial CI was evaluated in both cell cultures and ventral midbrain tissue samples using the CI Enzyme Activity Assay Kit (Abcam, 109721) [44]. ATP levels were quantified in cells and ventral midbrain tissues using the Enhanced ATP Assay Kit (Beyotime, S0027) [45]. Mitochondrial membrane potential (MMP) (ΔΨ) was assessed in cells by JC-10 staining (Yeasen, 40752ES60) [46]. All assays were conducted strictly according to the manufacturers' instructions.

### RNA interference

Small interfering RNA (siRNA) oligonucleotides targeting *SENP1* were synthesized by GenePharma (Shanghai, China). Transfection of siRNA into cells was performed using Lipofectamine™ RNAiMAX Transfection Reagent (Invitrogen-Thermo Fisher Scientific; Carlsbad, CA). Briefly, siRNA duplexes and Lipofectamine RNAiMAX reagent were separately diluted in Opti-MEM™ I Reduced Serum Medium (Gibco BRL; Grand Island, NY). The two diluted solutions were then mixed and incubated to allow for complex formation. This complex was added to the cells to achieve a final siRNA concentration of 20 nmol/L. The cells were incubated for 4–6 h before the medium was replaced with complete culture medium, and subsequently cultured for a total of 60 h prior to cell collection. The efficiency of *SENP1* knockdown was confirmed by Western blot analysis. The specific siRNA sequences used for gene silencing are as follows:

- SENP1_ siRNA 1: 5′-CUCGAUGUCUUAGUUCCAGUA-3′;
- SENP1_ siRNA 2: 5′-UGACCAUUACACGCAAAGAUA-3′;
- SENP1_ siRNA 3: 5′-CCGAAAGACCUCAAGUGGAUU-3′;
- SENP1_ siRNA 4: 5′-GCGCCAGAUUGAAGAACAGAA-3′

### Enzyme‑linked immunosorbent assay (ELISA)

After incubation, the culture medium was centrifuged to remove cell debris, and cytokine levels were assessed using ELISA kits (IL-6: Absin, abs520004; Shanghai, China; TNF-α: Absin, abs520010) following the manufacturer’s guidelines. Cytokine concentrations were quantified based on standard curves, with each sample analyzed in triplicates.

### Statistical analysis

All statistical analyses were performed using GraphPad Prism (version 8) in a blinded manner. Data normality was assessed using the Shapiro–Wilk test. For normally distributed data, Student's* t*-test was used for two-group comparisons. One-way ANOVA or two-way ANOVA was used for multiple group comparisons involving one or two factors, respectively. For non-normally distributed data, Mann–Whitney U test was applied for two-group comparisons, Kruskal–Wallis test for multiple-group comparisons with a single factor, and Aligned Rank Transform ANOVA for two-factor designs. *Post-hoc* analyses included Tukey's test following one-way ANOVA, Sidak's test for specific comparisons after two-way ANOVA, Dunn's test after Kruskal–Wallis, and Wilcoxon rank-sum test for Aligned Rank Transform ANOVA. *P* < 0.05 (two-sided) was considered statistically significant for all analyses.

## Results

### MPTP exposure increases the levels of acetylated proteins in mitochondria

**Fig. 1:**
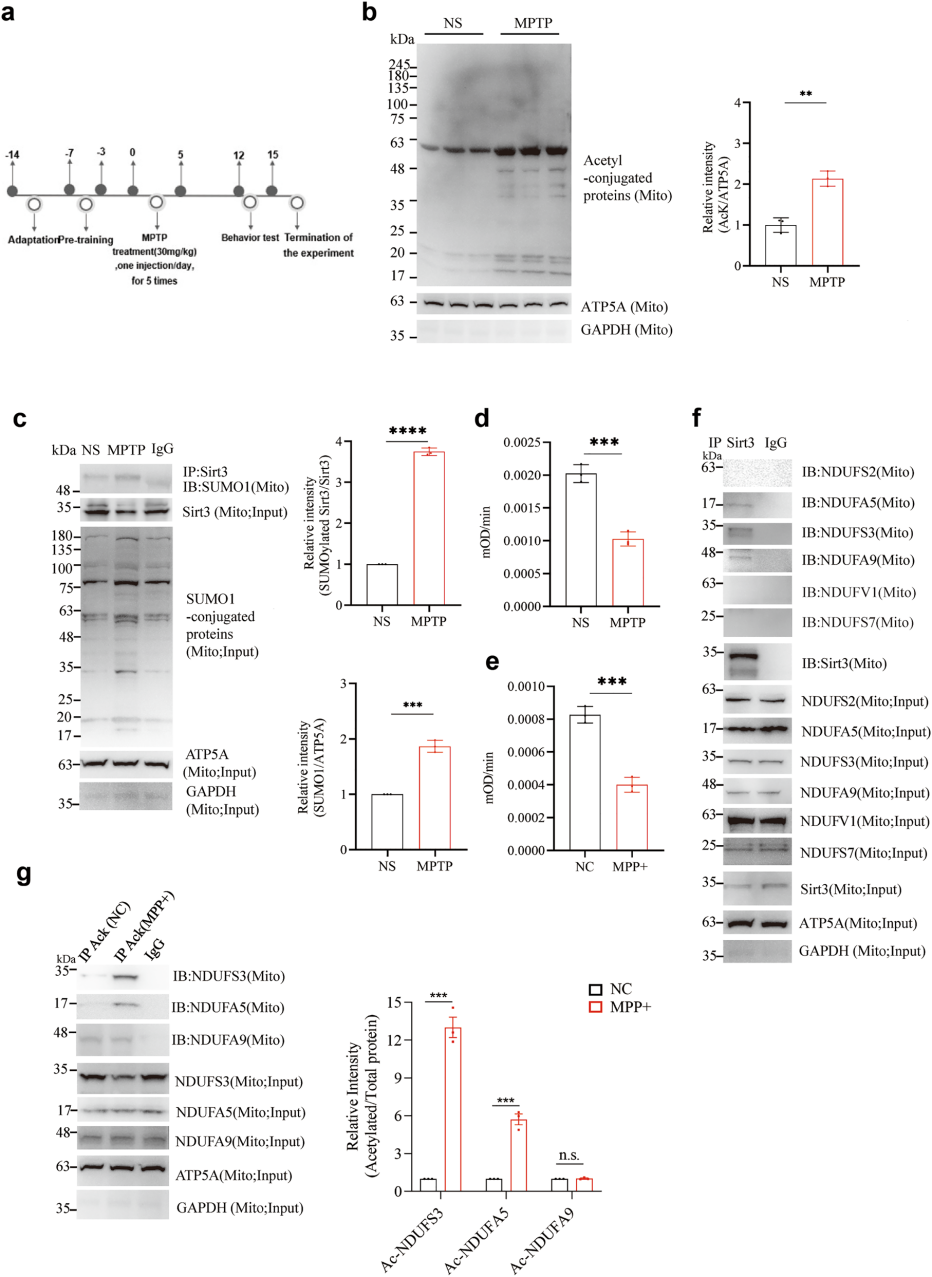
MPTP exposure diminished activities of Sirt3, NDUFA5, NDUFS3, and complex I (CI). **a** Experimental procedure. MPTP or saline was administered subcutaneously in 3-month-old male mice at the same time each day for 5 days. The mice were tested for motor behavior on days 12–14 and sacrificed on day 15 for subsequent experiments. **b** Mitochondria were isolated from the ventral midbrain of 3-month-old WT mice injected with MPTP or saline. Acetyl-conjugated proteins were blotted with anti-pan-AcK antibody and quantified from 3 biological replicates. **c** Mitochondrial lysates from the ventral midbrain region of 3-month-old WT mice injected with MPTP or saline were immunoprecipitated with IgG (rabbit) or anti-Sirt3. Then the precipitated proteins were blotted with anti-SUMO1. The total level of Sirt3 in the mitochondria was also detected with anti-Sirt3 antibody. Relative intensities of SUMOylated Sirt3 to total Sirt3 and SUMO1-conjugated proteins to ATP5A were quantified from 3 biological replicates. Data expressed as mean ± SD, *n* = 3 mice. **d** Activity of mitochondrial CI in the ventral midbrain tissue from WT mice treated with MPTP or saline. Data expressed as mean ± SD, *n* = 3 mice. **e** Activity of mitochondrial CI in SH-SY5Y cells treated with MPP^+^ (800 μmol/L) or culture medium. Data expressed as mean ± SD, *n* = 3. **f** Mitochondrial lysates from SH-SY5Y cells were immunoprecipitated with IgG (rabbit) or anti-Sirt3. The precipitated proteins were blotted with antibody for Sirt3, NDUFS3, NDUFA5, NDUFA9, NDUFV1, NDUFS2 or NDUFS7. The total levels of Sirt3, NDUFS3, NDUFA5, NDUFA9, NDUFV1, NDUFS2 and NDUFS7 in the mitochondria were also detected with the above-mentioned antibodies. **g** Mitochondrial lysates extracted from SH-SY5Y cells treated with MPP^+^ or culture medium were immunoprecipitated with IgG (rabbit) or anti-pan-AcK. The precipitated proteins were blotted with antibody for NDUFS3, NDUFA5 or NDUFA9. The total levels of NDUFS3, NFUFA5 and NDUFA9 in the mitochondria were also detected with the above-mentioned antibodies. Relative intensities of acetylated to total proteins for 3 biological replicates are shown. Mean ± SD. Unpaired *t*-test; *P < 0.05, **P < 0.01, ***P < 0.001, ****P < 0.0001

Mitochondrial enzyme activity is largely regulated by acetylation modification [17]. To investigate the impact of MPTP on mitochondrial protein acetylation, MPTP (30 mg/kg, a pathogenic dose established to induce significant dopaminergic neuronal death and microglial activation [28, 47–50]) or saline was administered to 3-month-old wild-type male mice for 5 consecutive days (Fig. 1a). On days 12–14, motor function was evaluated and on day 15, the mice were sacrificed for subsequent experiments (Fig. 1a). Mitochondria were isolated from the ventral midbrain and protein acetylation was detected through immunoblotting using an anti-pan-AcK antibody. Our findings revealed a significant increase in the levels of acetylated proteins after MPTP injection (*P* = 0.0016; Fig. 1b), suggesting that MPTP exposure altered the acetylation status of mitochondrial proteins, potentially affecting their functions.

### MPTP exposure enhances SUMOylation of Sirt3

Given the observed increase in mitochondrial protein acetylation induced by MPTP, we next focused on Sirt3, a major mitochondrial NAD^+^-dependent deacetylase. Sirt3 plays a critical role in regulating mitochondrial protein acetylation and maintaining mitochondrial function. Notably, the enzymatic activity of Sirt3 is regulated by SUMOylation, a post-translational modification in which SUMO1 attaches to specific lysine residues on Sirt3. Previous studies have demonstrated that SUMOylation of Sirt3 inhibits its enzymatic activity, thereby impairing its ability to regulate mitochondrial acetylation [22]. To examine the influence of MPTP on the enzyme activity of Sirt3, we used the anti-Sirt3 antibody to immunoprecipitate Sirt3 from the mitochondrial lysates isolated from the ventral midbrain. The level of SUMOylated Sirt3 was measured by SDS-PAGE and immunoblotting for SUMO1. The level of SUMOylated Sirt3 was significantly increased in the MPTP-treated group compared to the saline-treated group (*P* < 0.0001; Fig. 1c). These results suggest that the increase of acetylation level observed following MPTP exposure is closely associated with the MPTP-induced upregulation of Sirt3 SUMOylation.

### MPTP exposure decreases the activity of mitochondrial CI and increases the acetylation levels of NDUFA5 and NDUFS3

Given the observed increase in SUMOylated Sirt3 induced by MPTP, we next investigated whether this change contributes to the impairment of mitochondrial CI function. To test this, we designed experiments to assess mitochondrial CI activity in the ventral midbrain samples from MPTP-treated mice and in SH-SY5Y cells treated with MPP^+^. We observed a significant decline in mitochondrial CI activity in MPTP-treated mice compared to saline-treated controls (*P* = 0.0006; Fig. 1d) and in MPP^+^-treated SH-SY5Y cells compared to medium-treated cells (*P* = 0.0004; Fig. 1e). These results support our hypothesis that MPTP/MPP^+^ impairs mitochondrial CI activity, potentially through mechanisms involving Sirt3 SUMOylation.

The mitochondrial CI consists of 45 subunits [13]. Prior studies have shown Sirt3 binding with subunits NDUFS2, NDUFS3, NDUFS7, NDUFA5, NDUFV1, and NDUFA9 [51]. Therefore, we subsequently investigated whether MPTP impairs CI function by modulating the activity of its subunits. In SH-SY5Y cells, Sirt3 was found to specifically bind to NDUFS3, NDUFA9, and NDUFA5 in extracted mitochondrial fraction (Fig. 1f). Furthermore, MPP^+^ treatment induced significant elevations of the acetylation levels of NDUFS3 (*P* = 0.0001) and NDUFA5 (*P* = 0.0004), but not of NDUFA9 (*P* = 0.6668) (Fig. 1g). These observations not only confirmed the documented decline in mitochondrial CI activity induced by MPTP exposure, but further revealed that this decline was linked to increased SUMOylation of Sirt3 by MPTP, leading to increased acetylation levels of NDUFA5 and NDUFS3 and impairment of their activity.

### MPTP/MPP^+^ suppresses AMPK activity and inhibits SENP1 entry into mitochondria

**Fig. 2:**
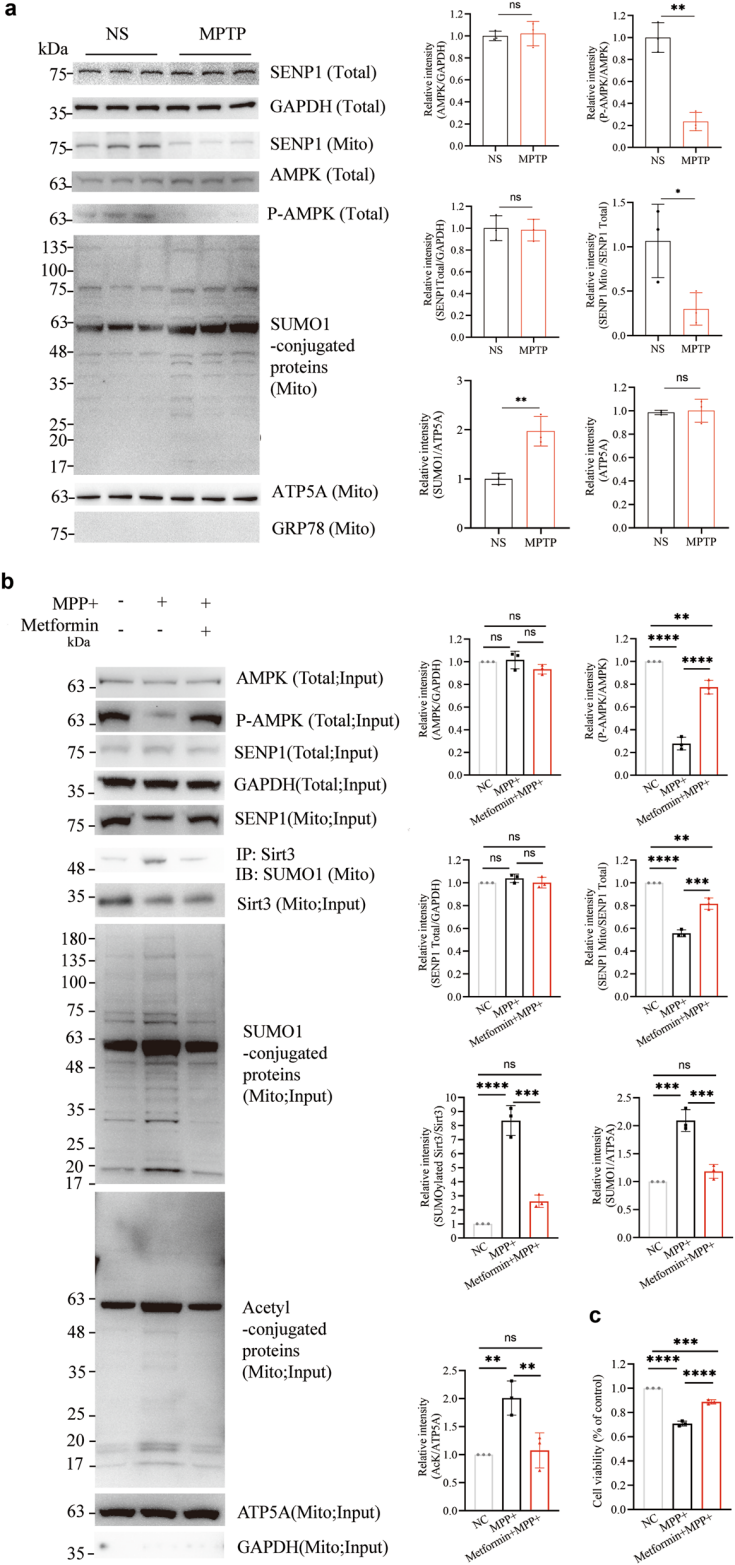
MPTP/MPP^+^ attenuates AMPK activity and diminishes mitochondrial translocation of SENP1. **a** Relative intensities of AMPK (total lysates), P-AMPK/AMPK (total lysates), SENP1 (total lysates), SENP1 (mitochondrial lysates) and SUMO1-conjugated proteins (mitochondrial lysates) in the ventral midbrain of 3-month-old WT mice injected with MPTP or saline. Data were from 3 biological replicates, and are expressed as mean ± SD, *n* = 3 mice. **b** Left, western blotting for SENP1 (total lysates), SENP1 (mitochondrial lysates), AMPK (total lysates) and P-AMPK (total lysates) from SH-SY5Y cells treated with culture medium, MPP^+^, or MPP^+^ and metformin (2 mmol/L) for 24 h, as well as blotting for SUMO1 in anti-Sirt3 immunoprecipitant from SH-SY5Y cell mitochondrial lysates. The total levels of Sirt3, SUMO1-conjugated proteins, and acetyl-conjugated proteins in mitochondrial lysates were also detected. Right, relative intensities of AMPK (total lysates), P-AMPK/AMPK (total lysates), SENP1 (total lysates), SENP1 (mitochondrial lysates), SUMOylated Sirt3 to total Sirt3 (mitochondrial lysates), SUMO1-conjugated proteins (mitochondrial lysates) and acetyl-conjugated proteins (mitochondrial lysates) from 3 biological replicates. Data expressed as mean ± SD, *n* = 3. **c** SH-SY5Y cells were treated with culture medium, MPP^+^, or MPP^+^  + metformin for 24 h. CCK8 assay was performed to measure cell viability. Data expressed as mean ± SD, *n* = 3. One-way ANOVA with Tukey's *post-hoc* test; *P < 0.05, **P < 0.01, ***P < 0.001, ****P < 0.0001

Next, we explored the precise mechanism by which MPTP increases the level of SUMOylated Sirt3. SUMOylation of Sirt3 is dynamically regulated by SENP1, a SUMO-specific protease that mediates de-SUMOylation. This process requires the translocation of SENP1 to mitochondria [22]. We observed a significant reduction (*P* = 0.0428) of translocation of SENP1 into the mitochondria (Fig. 2a) and a substantial upregulation (*P* = 0.0065) of mitochondrial SUMOylated proteins (Fig. 2a), while the total level of SENP1 remained unchanged (*P* = 0.8502; Fig. 2a) in the ventral midbrain in the MPTP-treated group compared with the saline group.

Previous research has demonstrated that the mitochondrial translocation of SENP1 is facilitated by p-AMPK [43]. We then speculated whether MPTP/MPP^+^ treatment causes dysregulation of p-AMPK. Our results showed a significant reduction in p-AMPK level (*P* = 0.0011) without altering the total AMPK level (*P* = 0.7795; Fig. 2a) in the ventral midbrain of MPTP-treated animals compared to the saline-treated animals.

Next, we extended our analysis to an in vitro system using SH-SY5Y cells treated with MPP^+^. Similarly, we observed a significant reduction in p-AMPK level (*P* < 0.0001; Fig. 2b) without affecting the total AMPK level (*P* = 0.9751; Fig. 2b) compared to untreated controls. To determine the functional consequences of reduced p-AMPK, we assessed SENP1 mitochondrial translocation, which we found was significantly decreased in the MPP^+^-treated cells (*P* < 0.0001; Fig. 2b) compared to untreated controls. This was accompanied by significant increases of SUMOylated Sirt3 level (*P* < 0.0001; Fig. 2b) and mitochondrial SUMOylated proteins (*P* = 0.0001; Fig. 2b) compared to untreated controls. Furthermore, these changes were associated with elevated levels of acetylated proteins in mitochondrial lysates (*P* = 0.0065; Fig. 2b) compared to untreated controls, suggesting downstream disruption of mitochondrial protein deacetylation. Finally, to evaluate the overall cellular impact of these mitochondrial dysfunctions, we measured cell viability and observed a significant decline in the viability of MPP^+^-treated cells compared to untreated controls (*P* < 0.0001; Fig. 2c). Collectively, these results support our hypothesis that MPTP/MPP^+^ disrupts p-AMPK, impairing SENP1 mitochondrial translocation and initiating a cascade of mitochondrial dysfunction, ultimately leading to reduced cell viability.

To further confirm the role of the AMPK–SENP1–Sirt3 axis in mitochondria and assess whether its activation could mitigate MPP^+^-induced toxicity, we treated cells with metformin, a well-established AMPK agonist [43]. The metformin-mediated AMPK activation effectively reversed the above-mentioned MPP^+^-induced dysregulation of the SENP1-Sirt3 pathway and cell toxicity in SH-SY5Y cells (Fig. 2b, c).

### Reduction of SUMOylated Sirt3 alleviates the MPTP-induced mitochondrial impairment

To further investigate the role of increased SUMOylation of Sirt3 in mitochondrial impairment caused by MPTP, we utilized cells and mice expressing a Sirt3 mutant (Sirt3 K288R in humans and Sirt3 K223R in mice), where the critical lysine residue required for SUMOylation is replaced with arginine, thereby preventing SUMOylation at this site. This mutation restores Sirt3 activity, enabling us to assess whether the absence of SUMOylation could mitigate mitochondrial dysfunction and related phenotypes caused by MPTP [22].

**Fig. 3:**
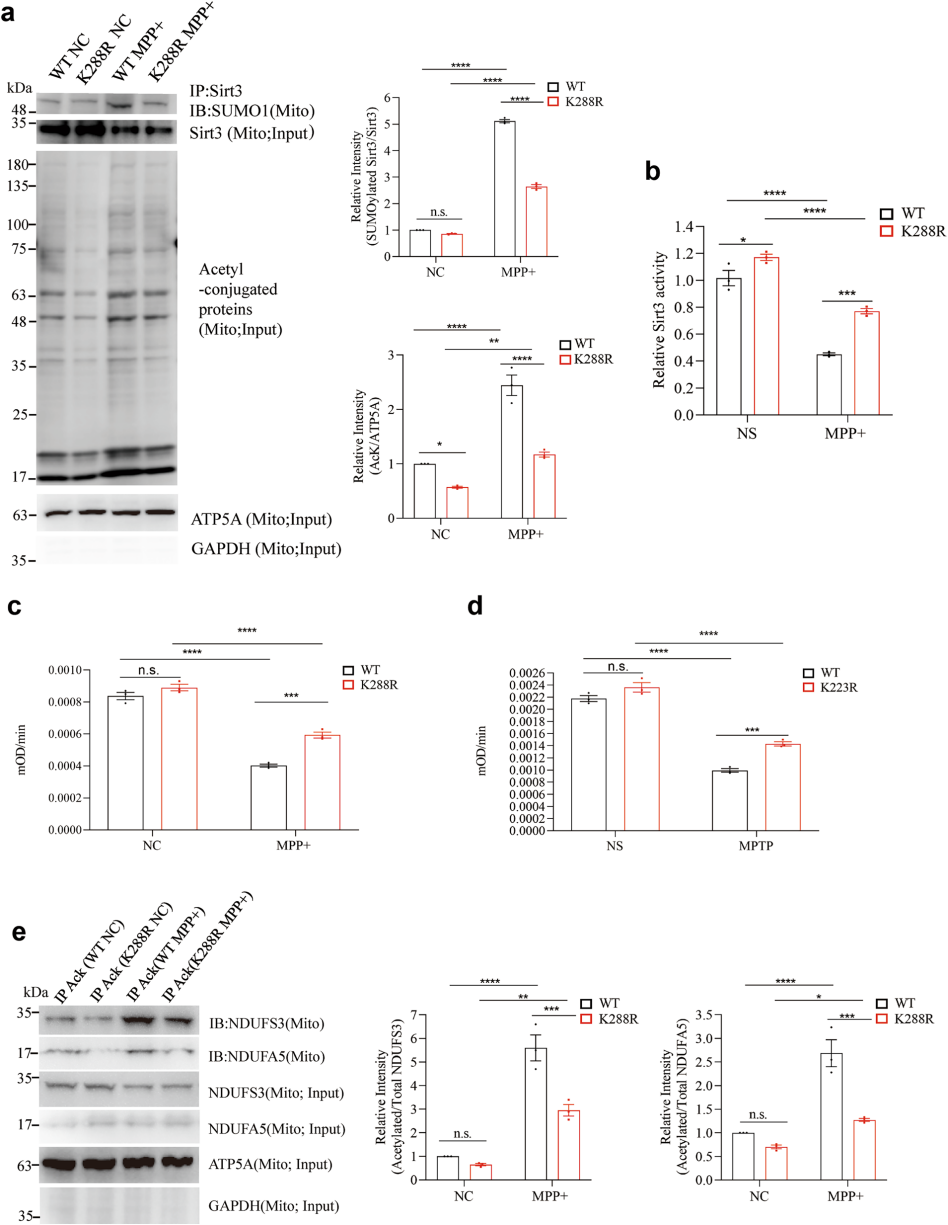
Diminishing SUMOylated Sirt3 level may confer protection against MPTP-induced impairments in activity of NDUFA5, NDUFS3 and complex I. **a** Left, mitochondrial lysates from Sirt3 WT and Sirt3 K288R SH-SY5Y cells treated with MPP^+^ or culture medium were immunoprecipitated with anti-Sirt3. The precipitated proteins were blotted with anti-SUMO1. The total levels of Sirt3 and acetyl-conjugated proteins were also detected. Right, relative intensities of SUMOylated Sirt3 to total Sirt3 and acetyl-conjugated proteins to ATP5A from 3 biological replicates. Data expressed as mean ± SD, *n* = 3. **b**, **c** Activity of Sirt3 (**b**) and complex I (**c**) in Sirt3 WT and Sirt3 K288R SH-SY5Y cells treated with MPP^+^ or culture medium. Mean ± SD, *n* = 3. **d** Activity of complex I in the ventral midbrain of Sirt3 WT and Sirt3 K223R mice treated with MPTP or saline. Mean ± SD, *n* = 3 mice. **e** Left panel, upper two lanes, blotting for NDUFS3 and NDUFA5 in anti-pan-AcK immunoprecipitant from mitochondrial lysates extracted from Sirt3 WT and Sirt3 K288R SH-SY5Y cells treated with MPP^+^ or culture medium. Lower lanes, Western blotting for NDUFS3 and NDUFA5 in mitochondrial lysates. Right, relative intensities of acetylated to total NDUFS3 and NDUFA5 from 3 biological replicates. Mean ± SD, n = 3. All data analyzed with two‐way ANOVA with Sidak's *post-hoc* test; *P < 0.05, **P < 0.01, ***P < 0.001, ****P < 0.0001

We found that MPP^+^ treatment significantly increased the level of SUMOylated Sirt3 in both WT and Sirt3 K288R cells, with a less pronounced increase in the Sirt3 K288R cells compared with the Sirt3 WT cells. Two-way ANOVA analysis showed a significant interaction between cell type and treatment (*P* < 0.0001; Fig. 3a), indicating that the Sirt3 K288R mutation partially mitigated the MPP^+^-induced upregulation of Sirt3 SUMOylation. Additionally, MPP^+^ treatment significantly increased protein acetylation levels in both cell types, with a less pronounced increase in the Sirt3 K288R cells compared to Sirt3 WT cells (*P* = 0.0025; Fig. 3a). Correspondingly, Sirt3 activity was significantly reduced following MPP^+^ exposure in both cell types, but the reduction was less pronounced in the Sirt3 K288R cells (*P* = 0.0355; Fig. 3b).

Further, MPP^+^ exposure significantly impaired the CI activity in both Sirt3 WT and Sirt3 K288R cells, but two-way ANOVA analysis revealed a significant interaction effect between genotype and treatment, indicating that the decline in CI activity was less pronounced in the Sirt3 K288R cells compared to Sirt3 WT cells (*P* = 0.0059; Fig. 3c). Similarly, in vivo analysis confirmed these findings, showing that MPTP administration significantly reduced CI activity in the ventral midbrain of both Sirt3 WT and Sirt3 K223R mice, with a less pronounced reduction in Sirt3 K223R mice (*P* = 0.0404; Fig. 3d).

We further investigated the effects of MPP^+^ on the acetylation levels of CI subunits. MPP^+^ exposure significantly increased acetylation of NDUFS3 and NDUFA5 in Sirt3 WT and Sirt3 K288R cells, but this increase was less pronounced in Sirt3 K288R cells. Statistical analysis confirmed significant interaction effects between cell type and MPP^+^ treatment for Ac-NDUFS3 (*P* = 0.0052) and Ac-NDUFA5 (*P* = 0.0049; Fig. 3e).

**Fig. 4:**
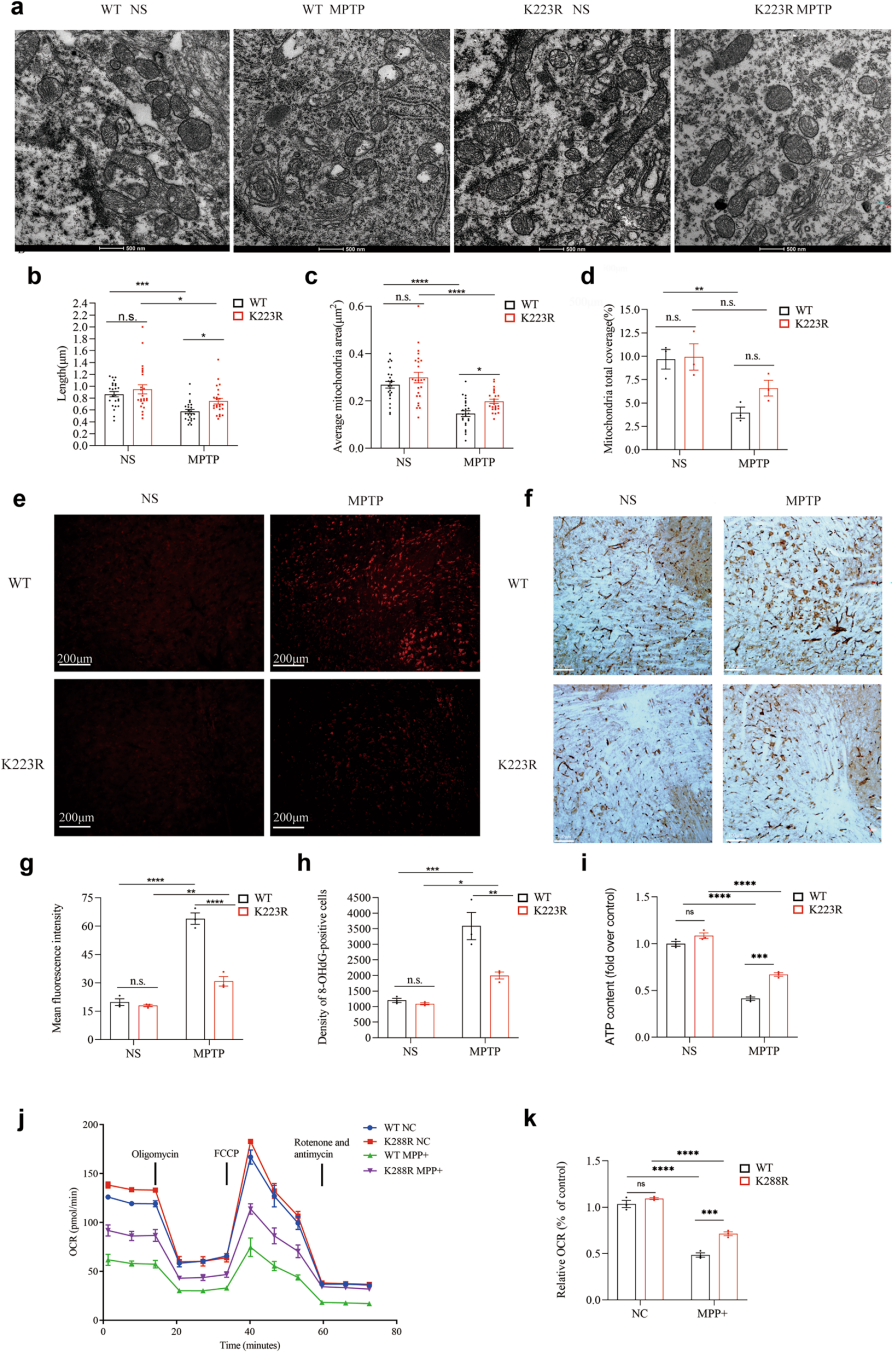
Diminishing SUMOylated Sirt3 level confers protection against MPTP-induced impairments of mitochondrial function. **a**–**d** Morphology of mitochondria in the substantia nigra of Sirt3 WT and Sirt3 K223R mice treated with MPTP or saline (**a**), as well as mitochondrial length (**b**), individual mitochondrial area (**c**) and mitochondrial area as a percentage of cytoplasmic area (**d**). Mean ± SD, *n* = 24 from 3 mice per group. **e**–**h** Fluorescence staining (**e**) and quantitative analysis (**g**) of oxidative stress (DHE) in the substantia nigra of Sirt3 WT and Sirt3 K223R mice treated with MPTP or saline. Immunohistochemical staining (**f**) and quantitative analysis (**h**) of oxidative stress (8-hydroxy-2′-deoxyguanosine, 8-OHdG) in the substantia nigra of Sirt3 WT and Sirt3 K223R mice treated with MPTP or saline. Mean ± SD, *n* = 3 mice. **i** ATP production in the ventral midbrain of Sirt3 WT and Sirt3 K223R mice treated with MPTP or saline. Mean ± SD, *n* = 3 mice. **j** Oxygen consumption rate (OCR) of Sirt3 WT and Sirt3 K288R SH-SY5Y stably transfected cells treated with MPP^+^ or culture medium, at baseline, after addition of oligomycin, after addition of FCCP and after addition of both rotenone and antimycin A, respectively. Mean ± SD, *n* = 3. **k** Extracellular oxygen consumption rates (EOCR) of Sirt3 WT and Sirt3 K288R SH-SY5Y stably transfected cells treated with MPP^+^ or culture medium. Mean ± SD, *n* = 3. Two‐way ANOVA with Sidak's *post-hoc* test; *P < 0.05, **P < 0.01, ***P < 0.001, ****P < 0.0001

The above experiments demonstrated that alterations in Sirt3 SUMOylation significantly influence the acetylation levels of NDUFS3 and NDUFA5, which, in turn, affect CI activity. We then assessed the effects of Sirt3 SUMOylation on mitochondrial function. Mitochondrial morphology is a crucial indicator of mitochondrial quality [52]. Compared to the Sirt3 WT mice, the Sirt3 K223R mice exhibited less impaired mitochondrial morphology after MPTP administration, as evidenced by preserved mitochondrial length (*P* = 0.0435; Fig. 4a, b) and individual mitochondrial area (*P* = 0.0345; Fig. 4c). However, the percent of total mitochondrial coverage within neuronal cytoplasm showed no significant differences between the two genotypes (*P* = 0.1975; Fig. 4d).

By using DHE [53] and 8-OHdG [54] as indicators of oxidative stress, we found that the MPTP treatment significantly increased oxidative stress in the SN of both Sirt3 WT and Sirt3 K223R mice; however, the oxidative stress was less pronounced in the Sirt3 K223R mice than in the Sirt3 WT mice (Fig. 4e–h). Two-way ANOVA confirmed significant interaction effects between genotype and MPTP treatment for both DHE (*P* < 0.0001; Fig. 4e, g) and 8-OHdG (*P* = 0.0118; Fig. 4f, h).

ATP production [55] and oxygen consumption [22] are critical indicators of mitochondrial respiration function. MPTP administration significantly reduced ATP production in the ventral midbrain of both genotypes, but the reduction was less pronounced in the Sirt3 K223R mice (*P* = 0.0065; Fig. 4i). Similarly, MPP^+^ exposure reduced ATP production in both Sirt3 WT and Sirt3 K288R cells, with a less pronounced decline in the Sirt3 K288R cells (*P* = 0.0203; Fig. S1a). The MPP^+^ treatment also significantly reduced both cellular and extracellular oxygen consumption in Sirt3 WT and Sirt3 K288R cells. However, the reduction of cellular oxygen consumption was less pronounced in the Sirt3 K288R cells, as evidenced by significant interaction effects at baseline (*P* = 0.0095; Fig. 4j and Fig. S1b), following oligomycin treatment (*P* = 0.0224; Fig. 4j and Fig. S1c), after FCCP treatment (*P* = 0.0159; Fig. 4j and Fig. S1d), and after rotenone and antimycin A treatment (*P* < 0.0001; Fig. 4j and Fig. S1e). Similarly, extracellular oxygen consumption was less reduced in Sirt3 K288R cells compared with Sirt3 WT cells (*P* = 0.0106; Fig. 4k).

Using MMP as a key indicator of mitochondrial integrity [56], we found that MMP was significantly reduced following MPP^+^ exposure in Sirt3 WT and Sirt3 K288R cells, with a significantly less pronounced reduction in the Sirt3 K288R cells (*P* = 0.0180; Fig. S1f).

### SUMOylated Sirt3 decrease alleviates the MPTP-induced dopaminergic neuronal loss and rescues behavioral deficits

The above results suggest that Sirt3 de-SUMOylation could alleviate mitochondrial impairments caused by MPTP/MPP^+^. We next investigated whether such alterations ultimately impact the MPTP-induced neurodegenerative phenotypes and behavioral deficits.

**Fig. 5:**
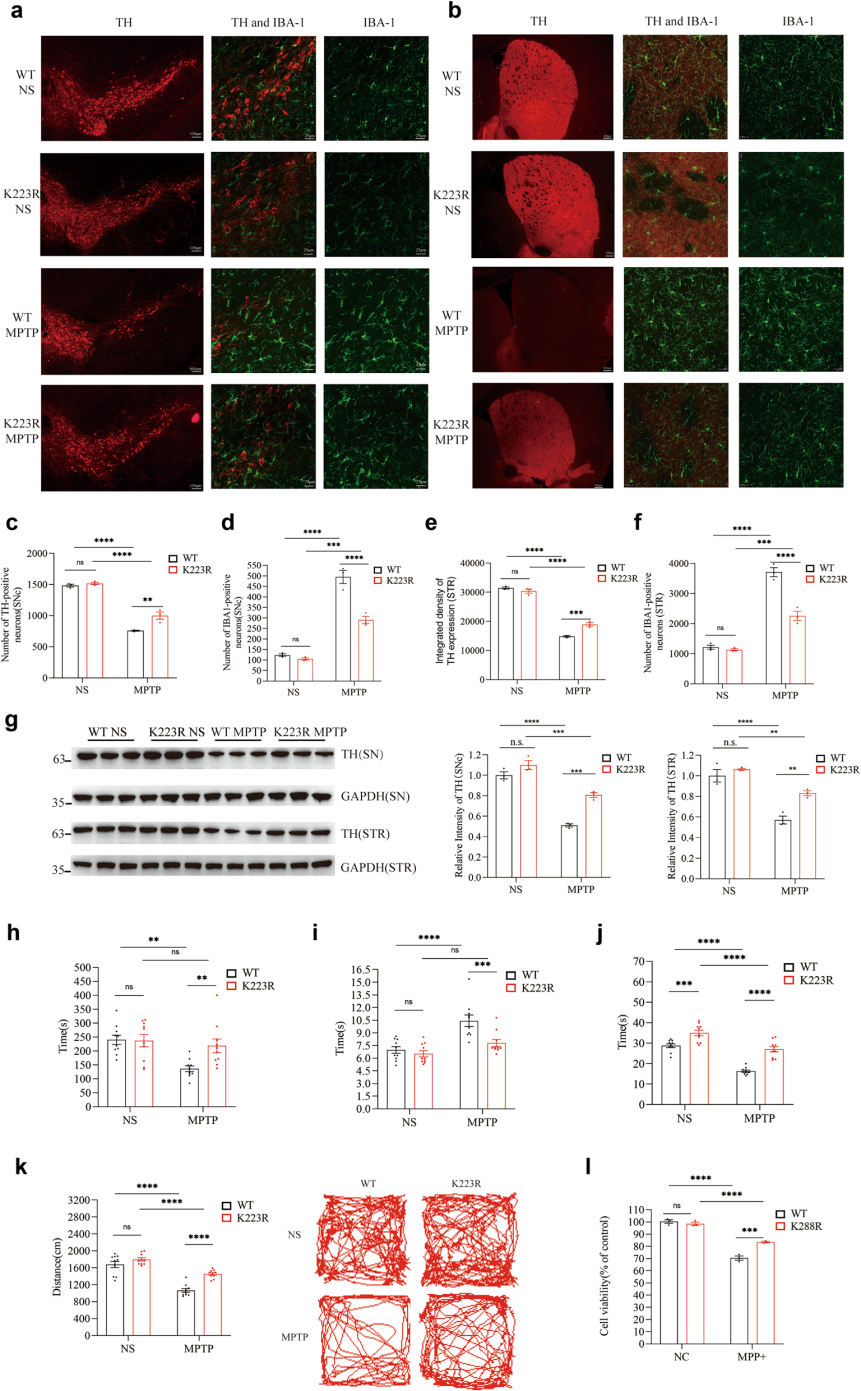
Reducing SUMOylated Sirt3 may protect against MPTP-induced dopaminergic neuronal death and behavioral disorders. **a**–**f** Immunofluorescent staining (**a**) and quantitative analysis of TH-positive cells (**c**) as well as IBA1-positive cells (**d**) in the SN of Sirt3 WT and Sirt3 K223R mice treated with MPTP or saline. Immunofluorescent staining (**b**) and quantitative analysis of TH-immunoreactive cells (**e**) as well as IBA1-immunoreactive cells (**f**) in the striatum of Sirt3 WT and Sirt3 K223R mice treated with MPTP or saline. Mean ± SD, *n* = 3 mice per group, scale bars, 100 μm (left) and 25 μm (right two) in **a**, 200 μm (left) and 25 μm (right two) in **b**. **g** Western blotting of TH in the substantia nigra (SN) and striatum (STR) of Sirt3 WT and Sirt3 K223R mice treated with MPTP or saline. Mean ± SD, *n* = 3. Relative intensities of TH from 3 biological replicates. Mean ± SD, *n* = 3 mice. **h**–**k** Performance of Sirt3 WT and Sirt3 K223R mice injected with MPTP or saline in the rotarod test (**h**), pole test (**i**), wire hanging test (**j**), and the open field test (**k**). Mean ± SD, *n* = 10. **l** Cell viability of Sirt3 WT and Sirt3 K288R SH-SY5Y stably transfected cells treated with MPP^+^ or culture medium for 24 h. Mean ± SD, *n* = 3. Two‐way ANOVA with Sidak's *post-hoc* test; *P < 0.05, **P < 0.01, ***P < 0.001, ****P < 0.0001

We found that MPTP administration significantly reduced dopaminergic neuron numbers and increased microglial activation in the SN of both Sirt3 WT and Sirt3 K223R mice. However, these changes were significantly less pronounced in the Sirt3 K223R mice compared to the Sirt3 WT mice (*P* = 0.0135, Fig. 5a, c; *P* = 0.0009, Fig. 5a, c). Similar trends were observed in the striatum, where MPTP significantly reduced the dopaminergic nerve fibers and increased microglial activation in both Sirt3 WT and Sirt3 K223R mice, with attenuated effects in Sirt3 K223R mice (*P* = 0.0009; Fig. 5b, e; *P* = 0.0003; Fig. 5b, f). To further elucidate the role of the AMPK–SENP1–Sirt3 pathway in neuroinflammatory regulation, we evaluated the effect of SENP1 knockdown on the production of inflammatory cytokines in BV2 microglia. We employed sub-threshold concentrations of MPP^+^ (0.01–0.02 mmol/L), which are insufficient for inducing significant microglial activation or secretion of inflammatory cytokines [57, 58]. While 0.02 mmol/L MPP^+^ treatment failed to induce inflammatory cytokine release in control BV2 cells, SENP1 knockdown cells exhibited significantly increased IL-6 and TNF-α secretion compared with control BV2 cells (Fig. S2), highlighting the essential role of the AMPK–SENP1–Sirt3 pathway in constraining microglial inflammatory responses.

We then performed Western blot analysis to assess the effects of the Sirt3 K223R mutation on TH levels. MPTP treatment significantly reduced TH levels in both the SN and the corpus striatum of Sirt3 WT and Sirt3 K223R mice. However, the reduction was less pronounced in Sirt3 K223R mice in both brain regions (*P* = 0.0152 for SN and *P* = 0.0331 for corpus striatum; Fig. 5g) compared to Sirt3 WT mice.

CCK8 assay showed that MPP^+^ exposure significantly reduced cell viability in both Sirt3 WT and Sirt3 K288R cell lines. However, the reduction was significantly less pronounced in the Sirt3 K288R cells compared to the Sirt3 WT cells (*P* = 0.0005; Fig. 5l). Two-way ANOVA confirmed a significant interaction effect between cell type and MPP^+^ treatment.

Behavioral tests were conducted to evaluate the effect of Sirt3 K223R mutation on MPTP-induced behavioral impairments. The Sirt3 K223R mutation attenuated MPTP-induced behavioral impairments, manifested as better performance in the rotarod test (*P* = 0.0337; Fig. 5h), less pole test time (*P* = 0.0290; Fig. 5i), longer wire-hanging time (*P* = 0.0458; Fig. 5j), and more distance travelled in the open field test (*P* = 0.0106; Fig. 5k), compared with the Sirt3 WT mice. Two-way ANOVA confirmed significant interaction effects between genotype and MPTP treatment across all behavioral tests. These results suggested that Sirt3 de-SUMOylation could mitigate the dopaminergic neuronal loss and motor impairments induced by MPTP.

## Discussion

In this study, we discovered that MPTP/MPP^+^ inhibits AMPK activity to impede the entry of SENP1 into mitochondria, resulting in increased SUMOylation level of Sirt3. This modification leads to decreased deacetylation activity of Sirt3 on NDUFA5 and NDUFS3, two subunits of CI, leading to reduced CI activity, mitochondrial dysfunction, and ultimately the degeneration of dopaminergic neurons.

While the inhibition of CI activity by MPTP has been documented previously, the precise mechanisms underlying this effect remain unclear. We demonstrate that the MPTP-induced CI dysfunction is mediated through the AMPK–SENP1–Sirt3 axis. The AMPK–SENP1–Sirt3 axis plays crucial roles in mitochondrial fatty acid metabolism [22], energy homeostasis [22], and T cell function [43]. In neuropathological contexts, the SENP1–Sirt3 axis is involved in the protection against ischemia/reperfusion injury [59]. In addition, AMPK and Sirt3 contribute independently to neuroprotection in PD models through their effects on mitochondrial function. Specifically, AMPK activation has been found to protect against dopamine depletion and mitochondrial pathologies in *LRRK2-* and parkin-mutant *Drosophila* models [60, 61]. Sirt3 has demonstrated neuroprotective effects in MPTP-induced PD models by deacetylating SOD2 and ATP synthase β [19], and in rotenone-induced PD models by deacetylating SDHA, thereby restoring ATP generation and mitochondrial energy metabolism [62].

Our study builds upon previous research and makes several significant advancements. Firstly, we present mechanistic evidence linking MPTP-induced CI dysfunction to the AMPK–SENP1–Sirt3 axis, addressing a critical gap in our understanding of how MPTP impairs mitochondrial function. Secondly, we identify that this axis affects the acetylation of CI subunits NDUFS3 and NDUFA5, providing molecular mechanisms underlying the effect of MPTP on CI. Previous research has identified potential interactions between Sirt3 and six CI subunits including NDUFA5 and NDUFS3. NDUFS3 is one of the core subunits of CI and plays a role in its biogenesis [63]. The cleavage of NDUFS3 could disrupt mitochondrial metabolism and generate reactive oxygen species, triggering programmed cell death [64, 65]. NDUFS3 mutation is reported to cause Leigh syndrome manifested with significantly decreased CI activity [66, 67]. NDUFA5 acts as a supernumerary subunit and is required for the assembly and stabilization of the electron transferring module in CI [68]. Ablation of NDUFA5 in mice elicits mild chronic encephalopathy with considerably declined CI levels and activity but no oxidative damage or neuronal death [69]. Collectively, our results underscore the significant role of increased acetylation levels of NDUFS3 and NDUFA5 in the decline of CI activity induced by MPTP. Finally, we demonstrate that the axis-related Sirt3 SUMOylation alteration confers significant protection against MPTP-induced toxicity, as evidenced by its ability to preserve mitochondrial function. These findings suggest a potential therapeutic strategy for mitigating neurotoxin-induced mitochondrial damage in PD.

Our study highlights the effect of the AMPK–SENP1–Sirt3 axis on microglia, a key player in neuroinflammation associated with PD. Immunofluorescence analyses revealed that MPTP treatment induced significant microglial activation, which was attenuated upon activation of the SENP1–Sirt3 axis (Fig. 5a–f). In BV2 microglia, silencing SENP1 significantly increased the secretion of pro-inflammatory cytokines, including IL-6 and TNF-α, following MPP^+^ treatment (Fig. S2a, b). These results suggest that the AMPK–SENP1–Sirt3 axis plays a critical role in the regulation of neuroinflammation by modulating microglial activation and cytokine secretion. This dual role in neuronal protection and microglial modulation underscores the multifaceted contribution of this axis to neuroprotection in PD.

## Conclusions

In conclusion, our study provides substantial evidence for the involvement of the AMPK–SENP1–Sirt3 axis in MPTP-induced CI dysfunction and PD-like pathology. We demonstrate that MPTP/MPP^+^ disrupts this pathway, leading to increased acetylation of NDUFS3 and NDUFA5, decreased CI activity, and ultimately PD progression. These findings not only deepen our understanding of the molecular mechanisms underlying PD, but also suggest potential new therapeutic strategies for mitigating neurotoxin-induced mitochondrial damage in PD and possibly other neurodegenerative disorders characterized by mitochondrial dysfunction.

## Supplementary Information

**Additional file 1**: **Figure S1** Reducing SUMOylated Sirt3 may confer protection against MPTP-induced impairments in mitochondrial function. **Figure S2** Silencing SENP1 exacerbates the secretion of pro-inflammatory cytokines IL-6 and TNF-α in MPP^+^-treated microglia.

**Additional file 2**: Uncropped Western blot images.

## Data Availability

The datasets used and/or analysed during the current study are available from the corresponding author on reasonable request.

## Abbreviations

PD: Parkinson's disease
MPTP: 1-methyl-4-phenyl-1,2,3,6-tetrahydropyridine
CI: Complex I
MPP^+^: 1-Methyl-4-phenylpyridinium ion
NAD^+^: Nicotinamide adenine dinucleotide
Sirt3: Sirtuin3
TH: Tyrosine hydroxylase
IBA1: Ionized calcium-binding adapter molecule 1
SN: Substantia nigra
DHE: Dihydroethidium
8-OHdG: 8-Hydroxy-2′-deoxyguanosine
OCR: Oxygen consumption rate
p-AMPK: Phosphorylated AMP-activated protein kinase
MMP: Mitochondrial membrane potential
FCCP: Trifluoromethoxy carbonylcyanide phenylhydrazone
