# Supplementary material for: Deficient AMPK-SENP1-Sirt3 signaling impairs mitochondrial complex I function in Parkinson’s disease model
Source: Transl Neurodegener. 2025 Jul 1;14:34. doi: 10.1186/s40035-025-00489-2 (PMC12211261; doi:10.1186/s40035-025-00489-2)
Supplement: Supplementary file 1 — Additional file 1: Figure S1 Reducing SUMOylated Sirt3 may confer protection against MPTP-induced impairments in mitochondrial function. Figure S2 Silencing SENP1 exacerbates the secretion of pro-inflammatory cytokines IL-6 and TNF-α in MPP+-treated microglia. [file 40035_2025_489_MOESM1_ESM.docx]

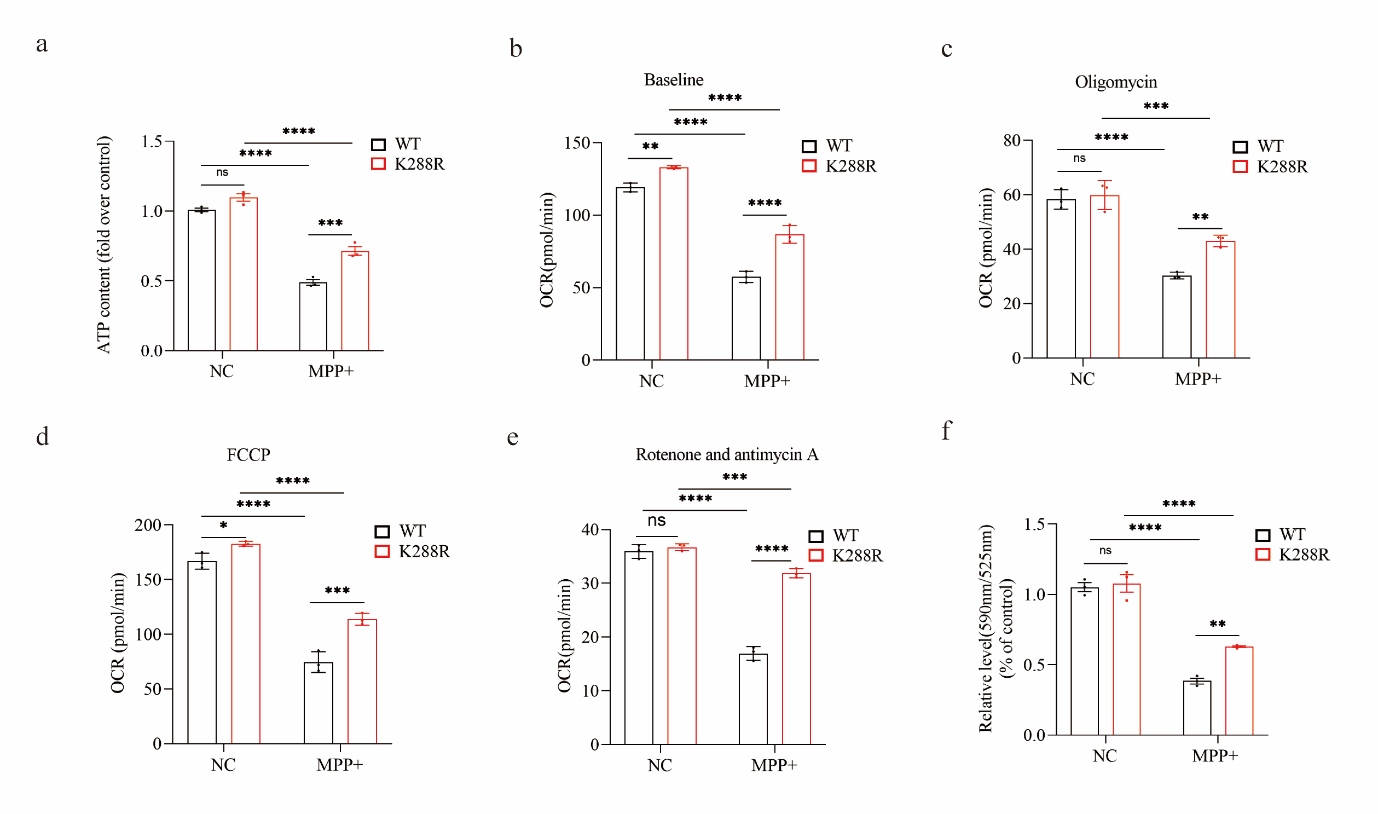


Fig. S1 Reducing SUMOylated Sirt3 may confer protection against MPTP-induced impairments of mitochondrial function.


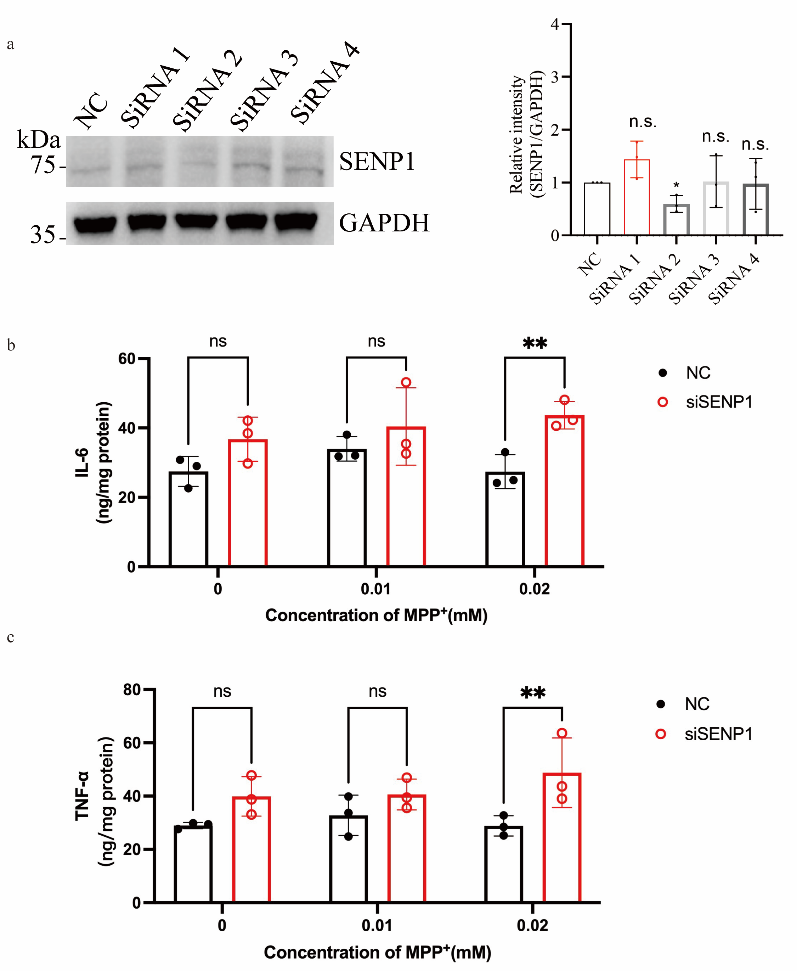


Fig. S2 Silencing SENP1 exacerbates the secretion of pro-inflammatory cytokines IL-6 and TNF-α in MPP^+^-treated microglia.
