# Supplementary material for: Deficient AMPK-SENP1-Sirt3 signaling impairs mitochondrial complex I function in Parkinson’s disease model
Source: Transl Neurodegener. 2025 Jul 1;14:34. doi: 10.1186/s40035-025-00489-2 (PMC12211261; doi:10.1186/s40035-025-00489-2)

Presentation of Uncropped Western Blot Images

Each slide contains two sections: on the left is the figure from the manuscript, while on the right are the corresponding uncropped images. To enhance clarity, if any protein marker in the uncropped images is not visible, an additional merged image showing both the uncropped blot and the protein marker is provided next to it.

Figure 1B

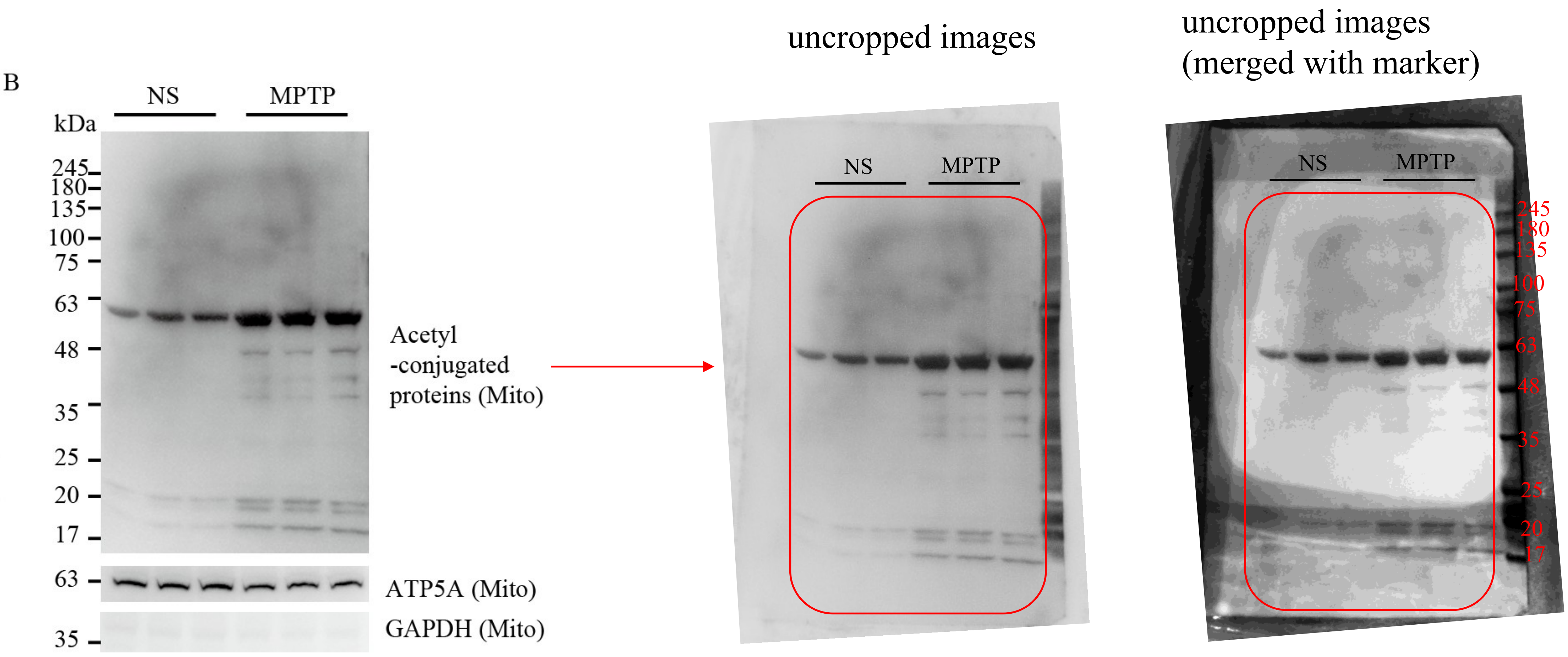

Acetyl-conjugated proteins(Mito)

Figure 1B

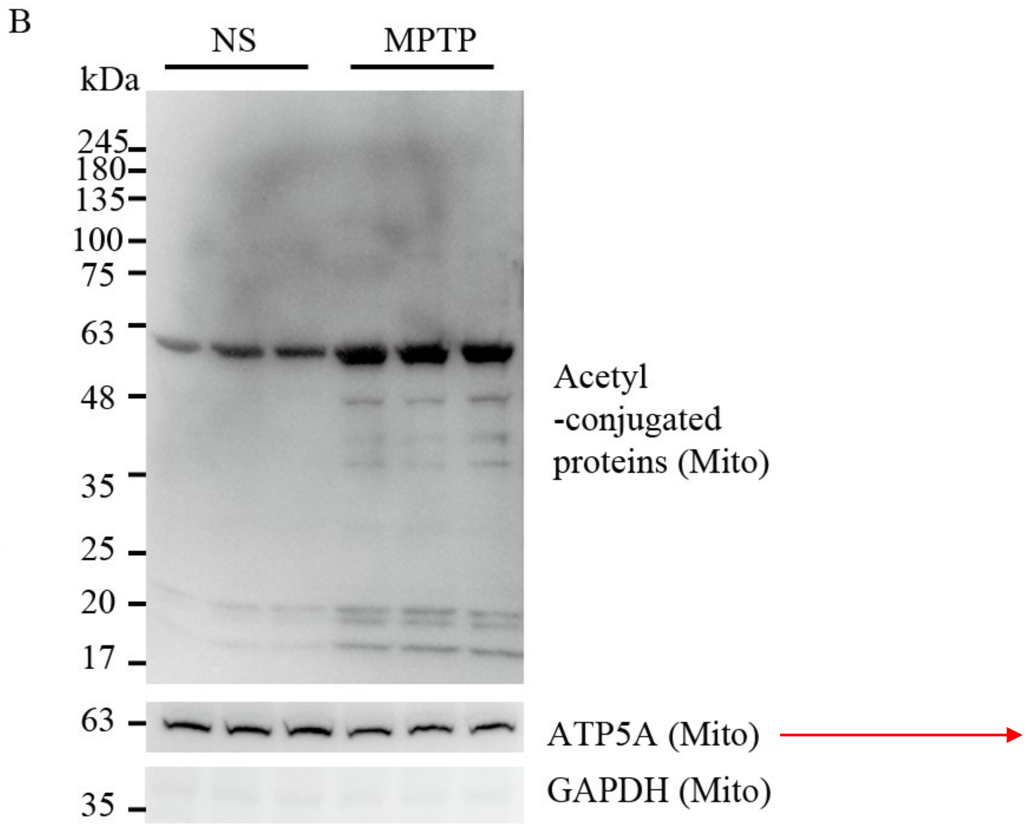

uncropped images

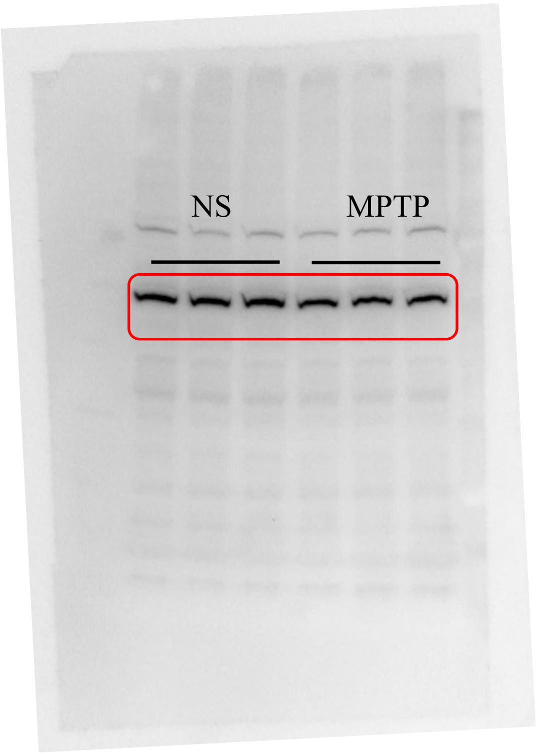

uncropped images
(merged with marker)

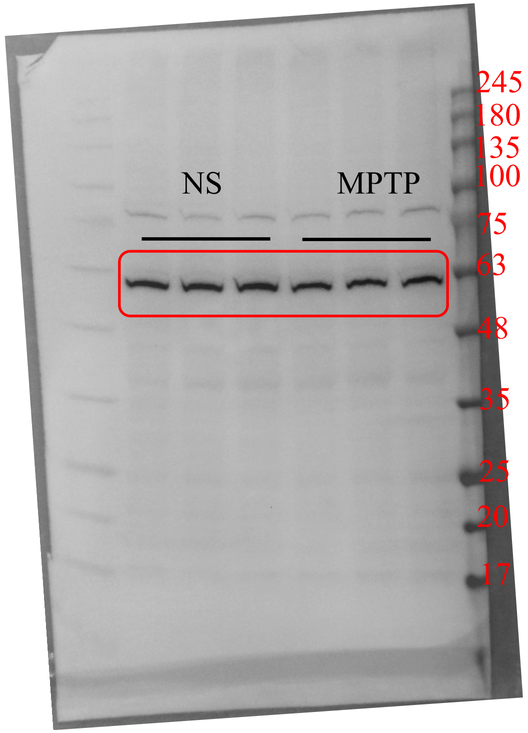

ATP5A(Mito)

Figure 1B

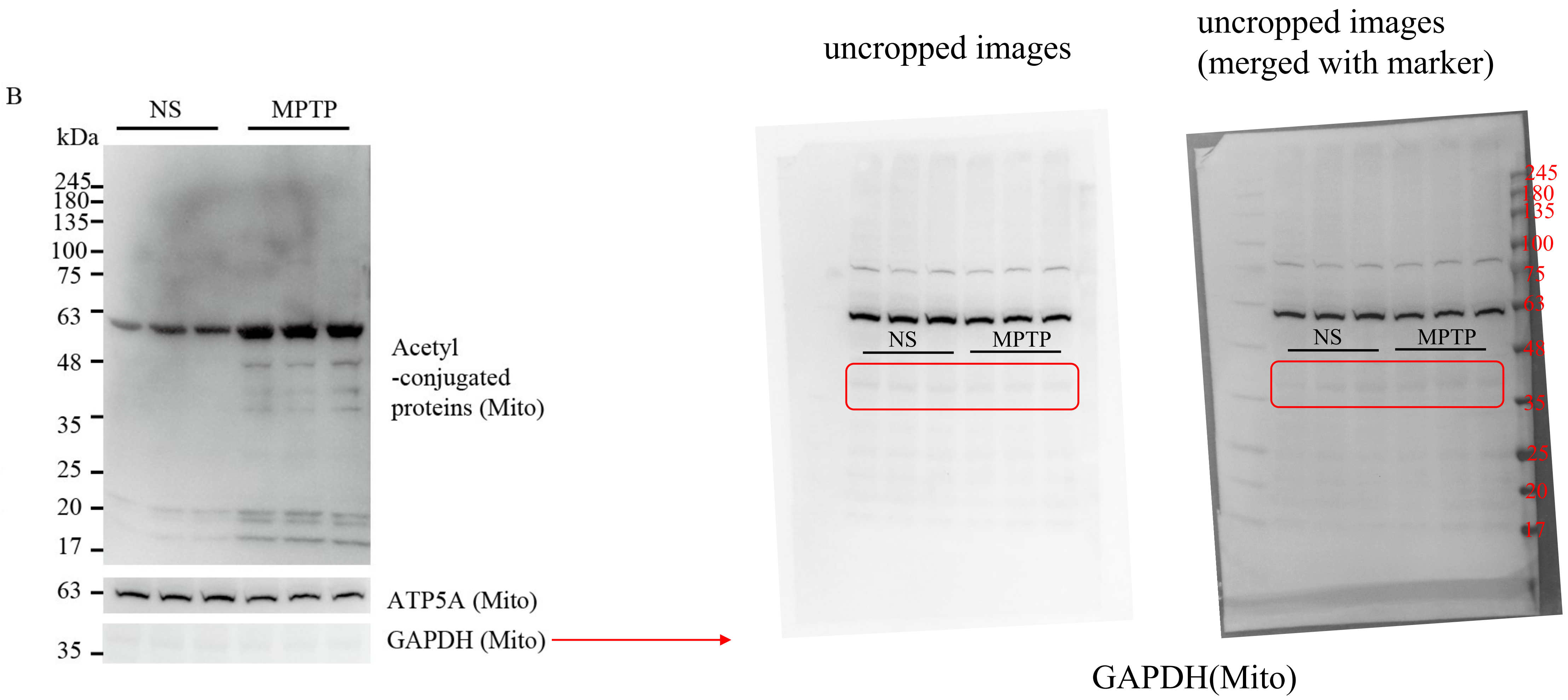
B
NS
MPTP
kDa
245
180
135
100
75
63
48
35
25
20
17
Acetyl
-conjugated
proteins (Mito)
63
ATP5A (Mito)
35
GAPDH (Mito)
uncropped images
NS
MPTP
uncropped images
(merged with marker)
245
180
135
100
75
63
48
35
25
20
17
NS
MPTP
GAPDH(Mito)

Figure 1C

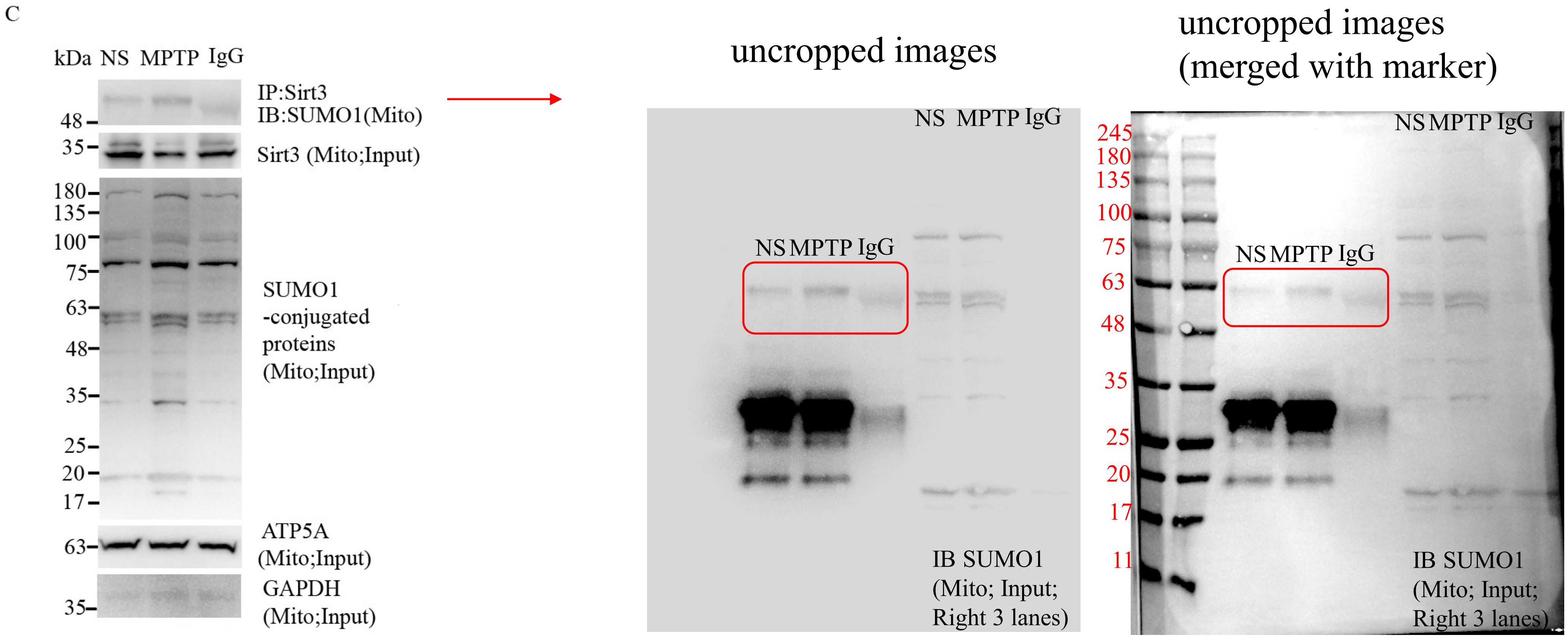

IP Sirt3 IB SUMO1(Mito; Left 3 lanes)

Figure 1C

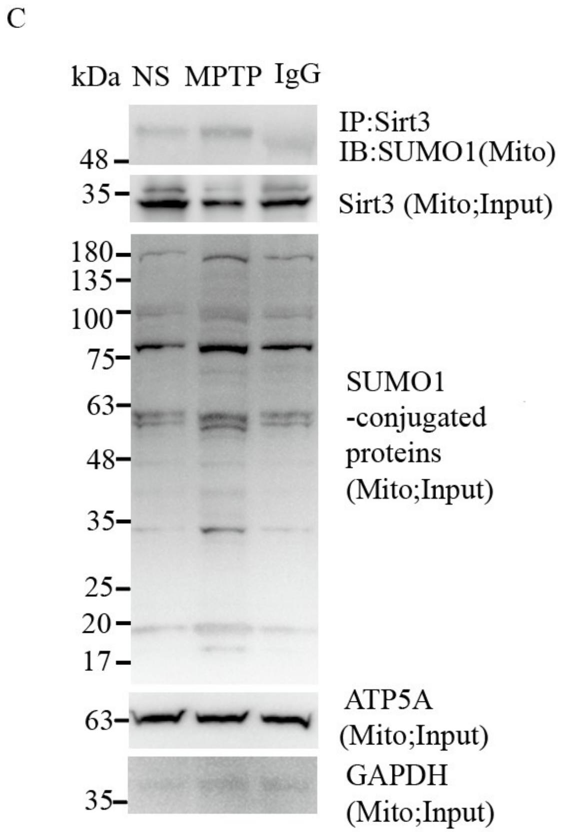

uncropped images

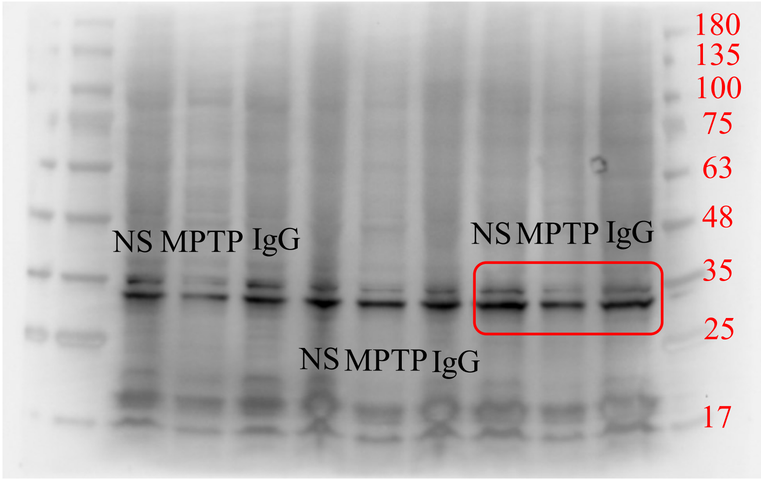

Sirt3 (Mito; Input)

Figure 1C

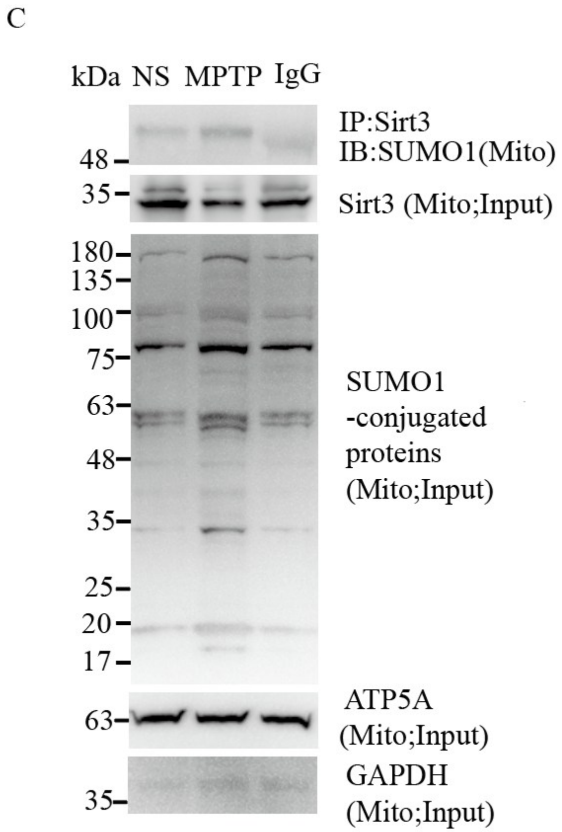

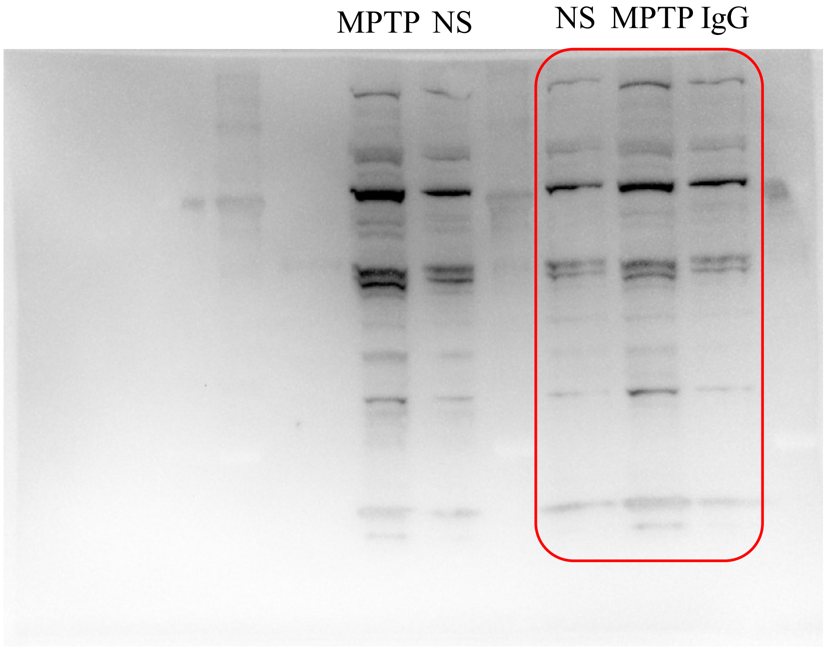

uncropped
images

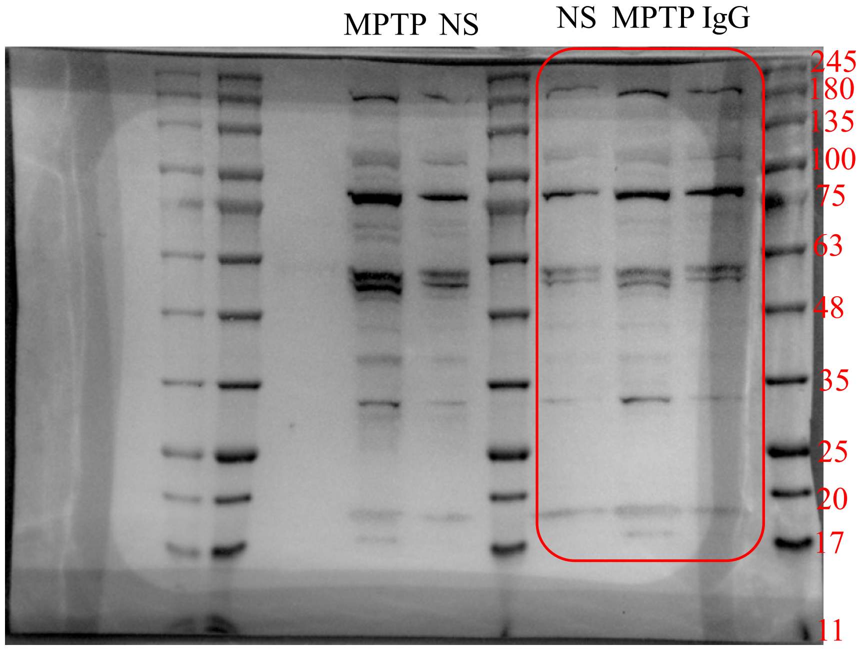

uncropped
images
(merged with marker)

SUMO1(Mito)

Figure 1C

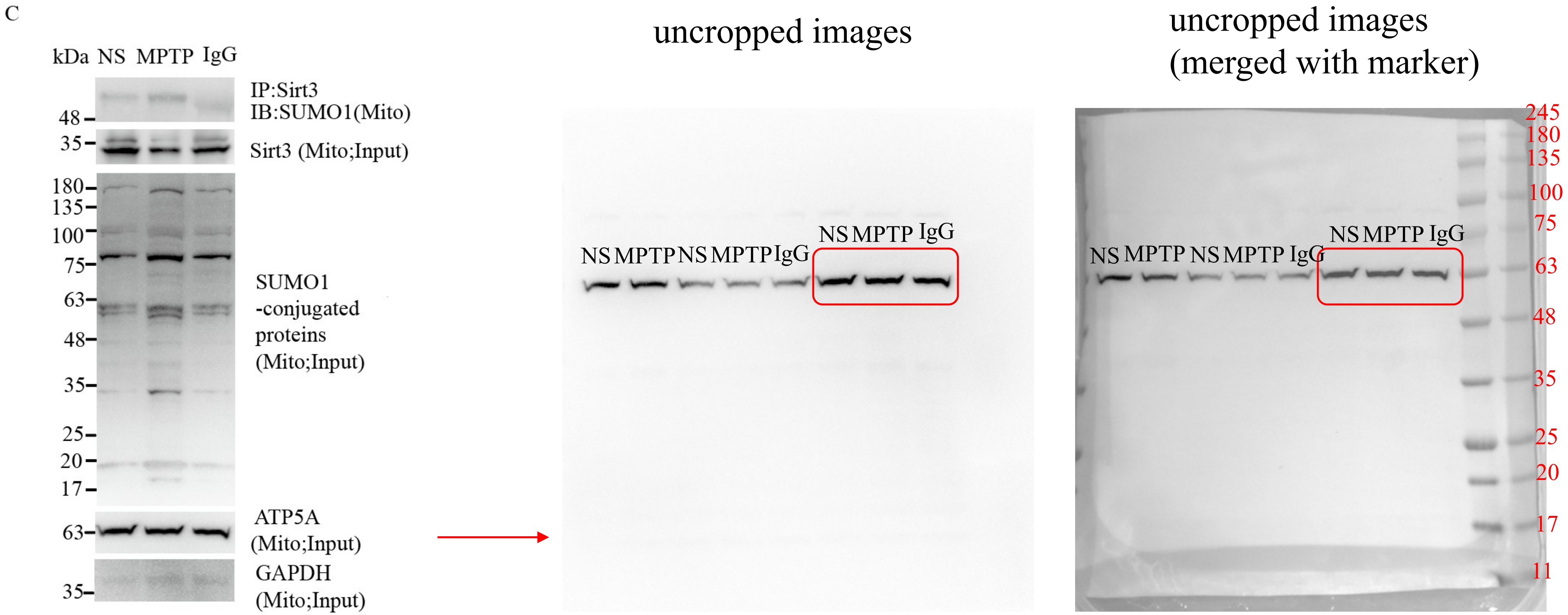

ATP5A (Mito; Input)

Figure 1C

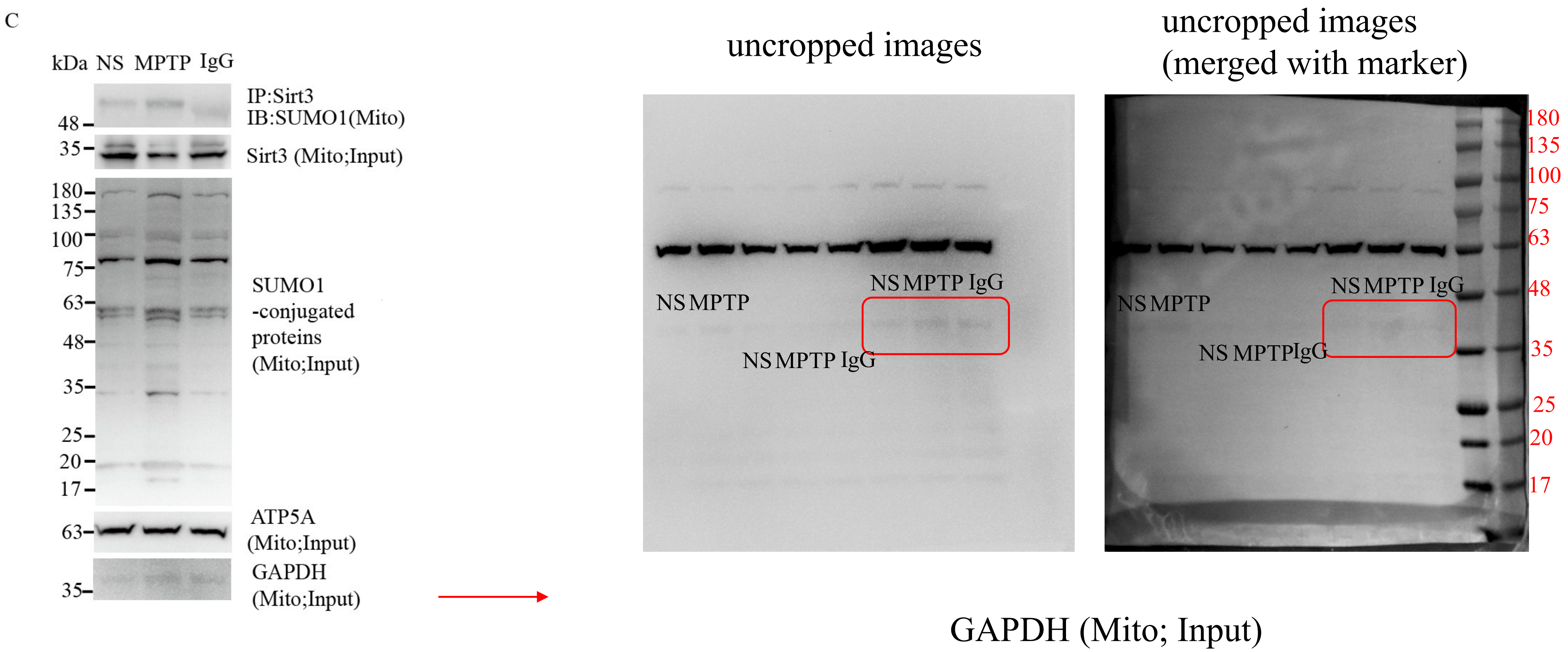
C
kDa NS MPTP IgG
48
35
180
135
100
75
63
48
35
25
20
17
63
35
IP:Sirt3
IB:SUMO1(Mito)
Sirt3 (Mito;Input)
SUMO1
-conjugated
proteins
(Mito;Input)
ATP5A
(Mito;Input)
GAPDH
(Mito;Input)
uncropped images
NS MPTP
NS MPTP IgG
NS MPTP IgG
uncropped images
(merged with marker)
NS MPTP
NS MPTP IgG
NS MPTPIgG
180
135
100
75
63
48
35
25
20
17
GAPDH (Mito; Input)

Figure 1F

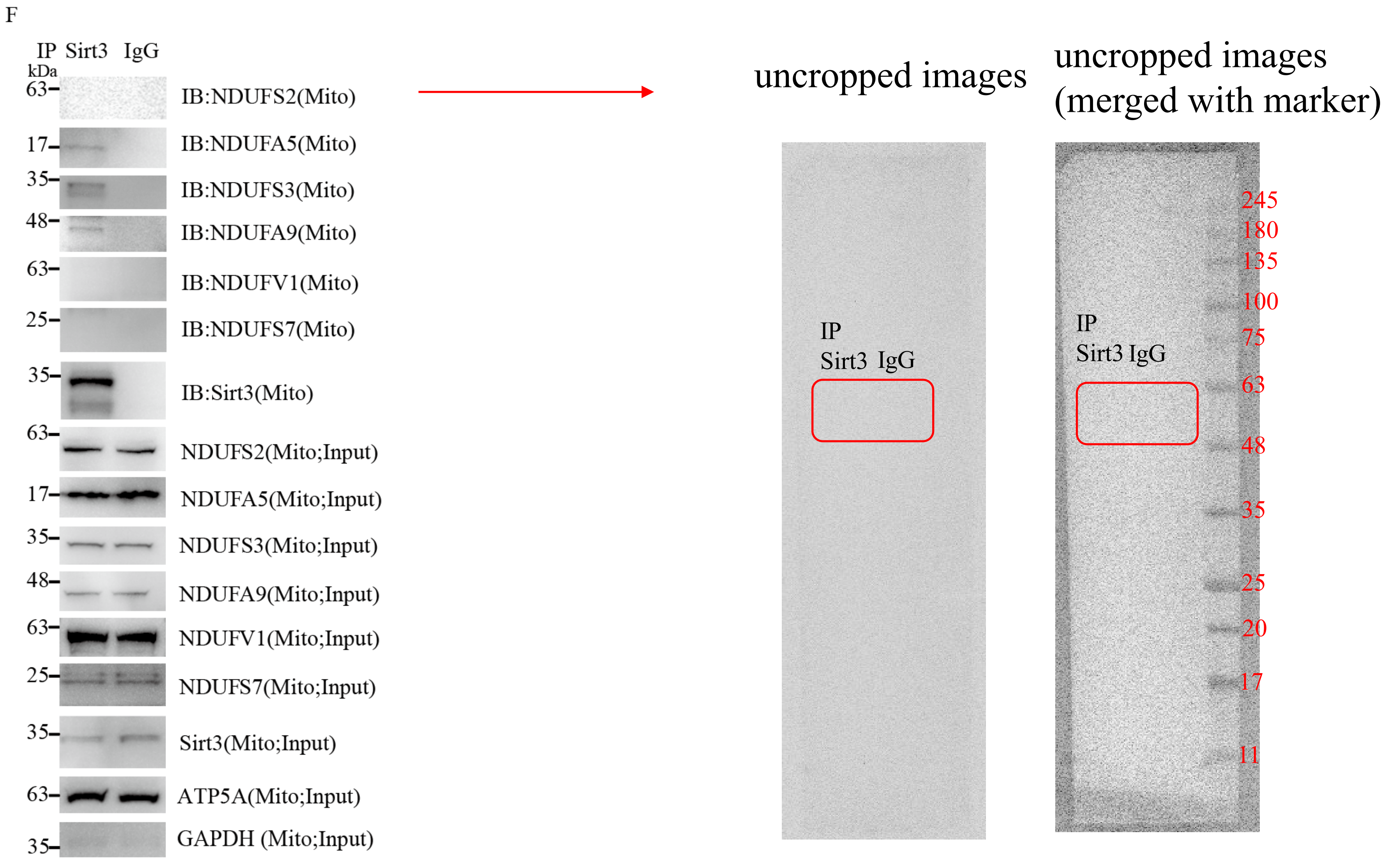

NDUFS2 (Mito)

Figure 1F

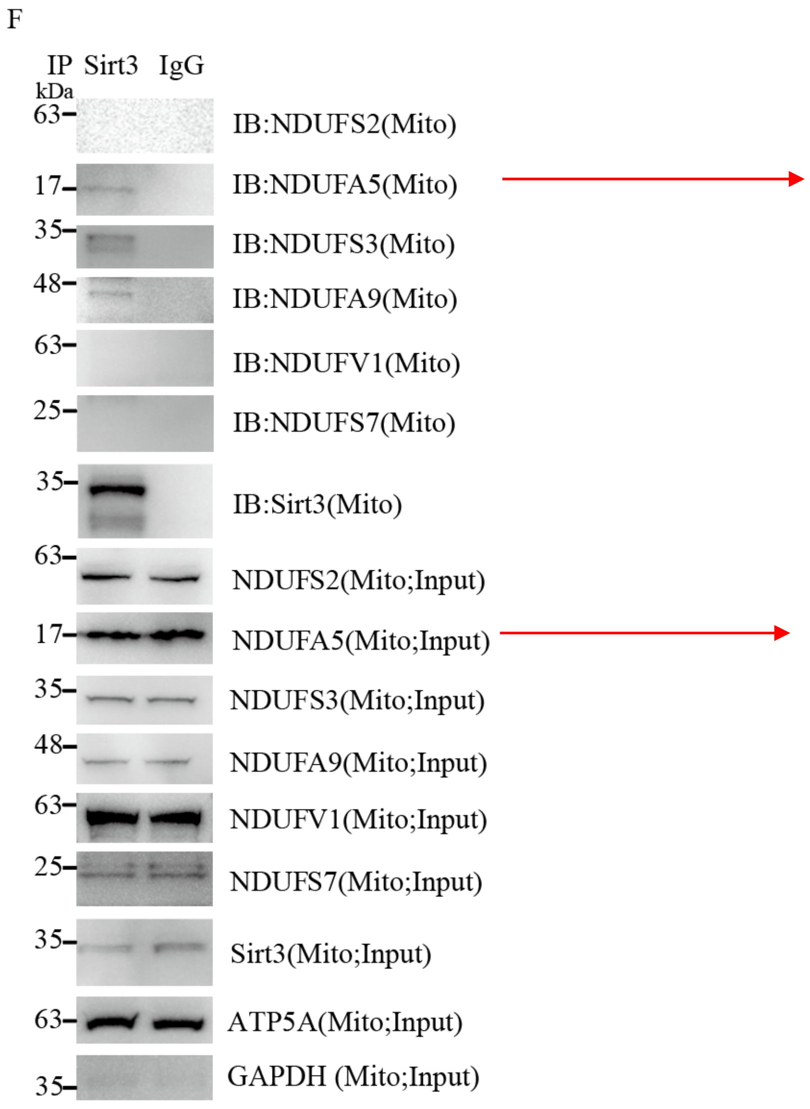

uncropped images

uncropped images (merged with marker)

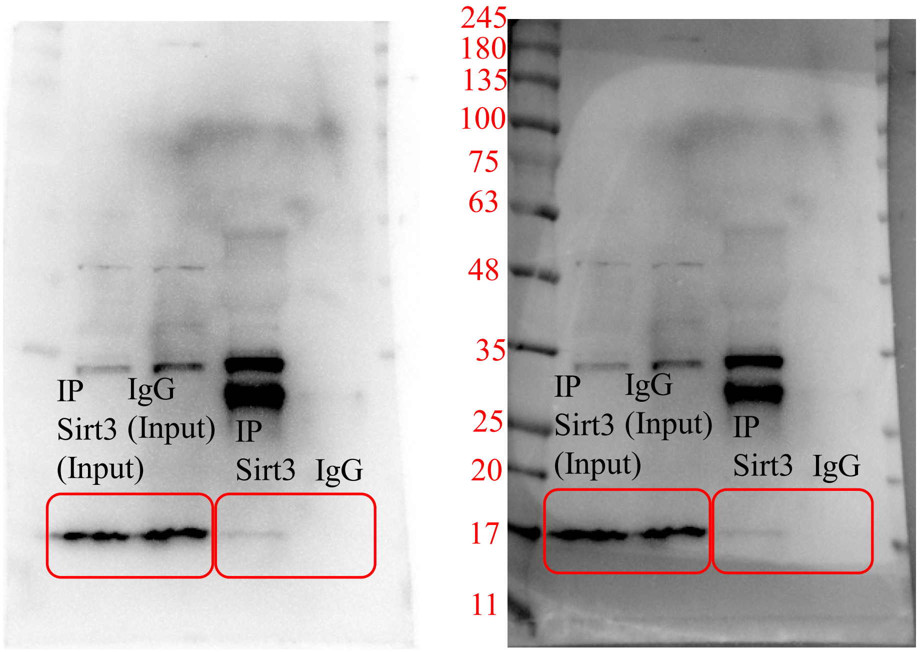

NDUFA5 (Mito; Input; Left 2 lanes) and NDUFA5 (Mito; Right 2 lanes)

Figure 1F

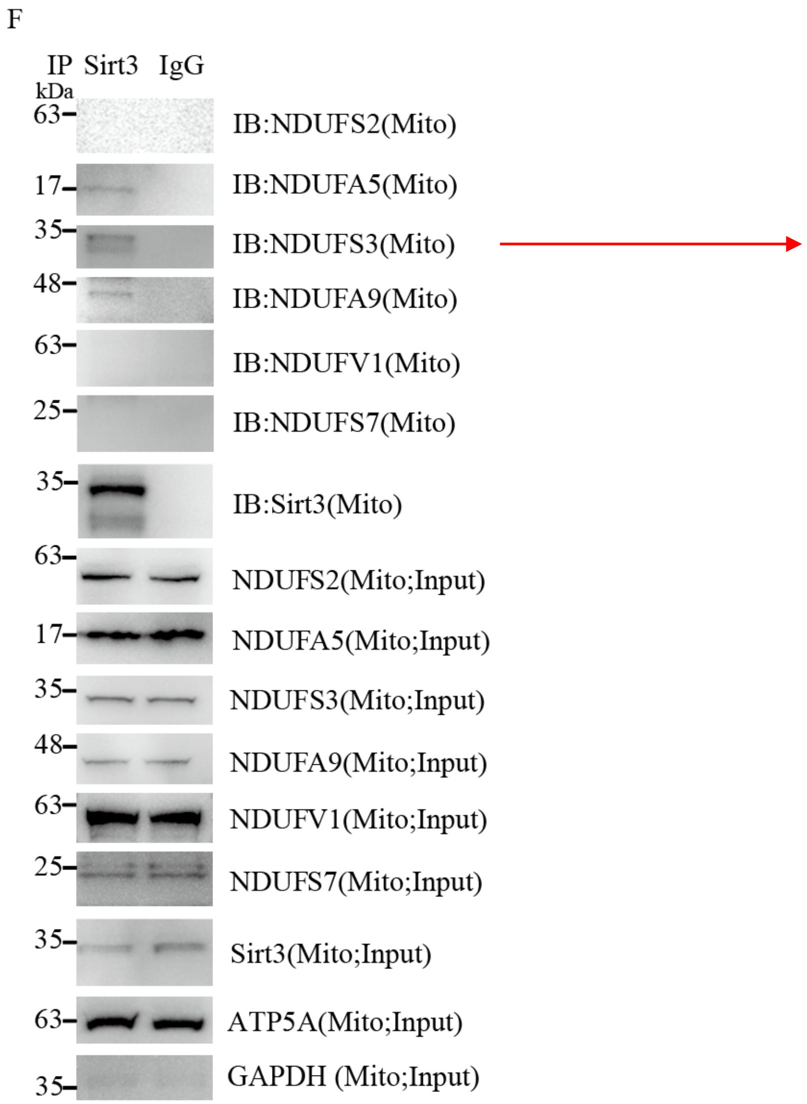

uncropped images

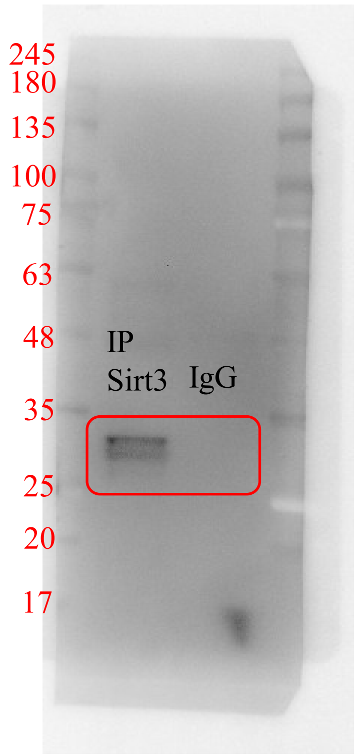

NDUFS3 (Mito)

Figure 1F

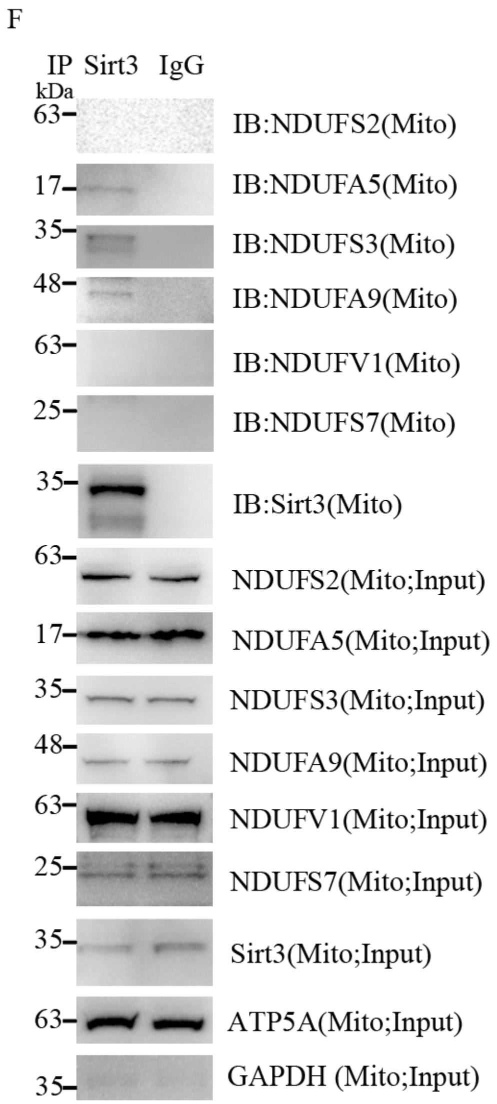

uncropped images

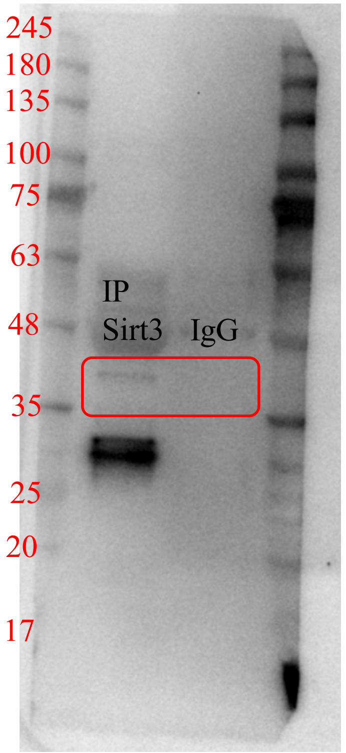

NDUFA9 (Mito)

Figure 1F

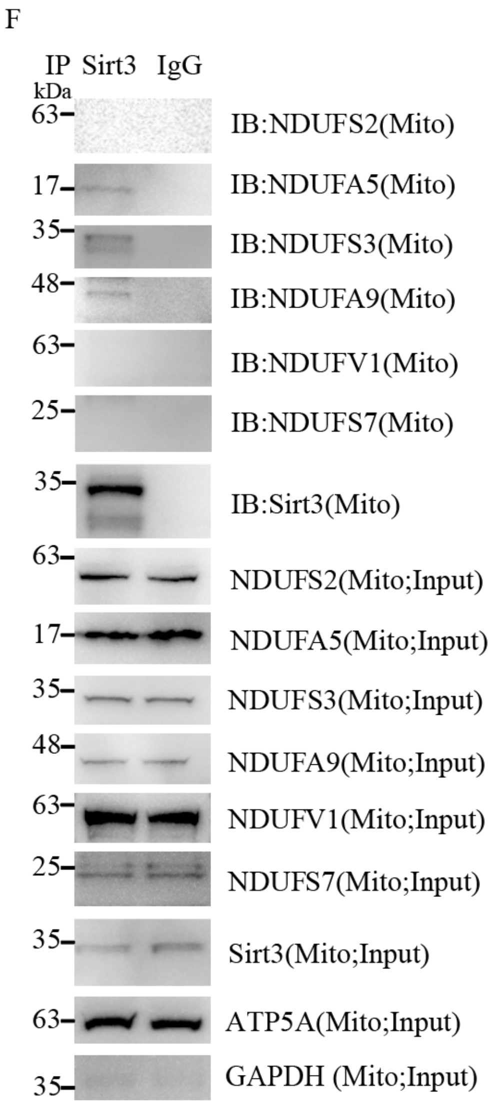

uncropped images

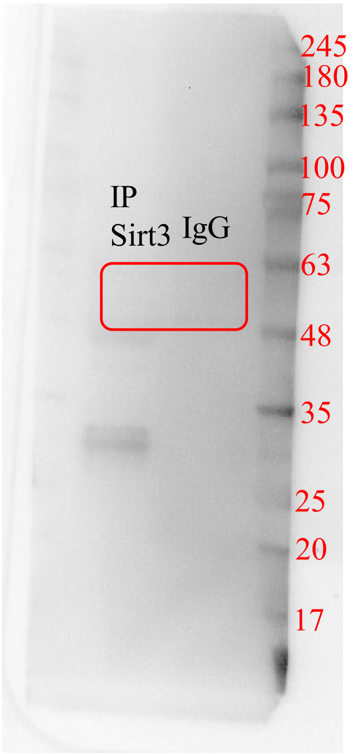

NDUFV1 (Mito)

Figure 1F

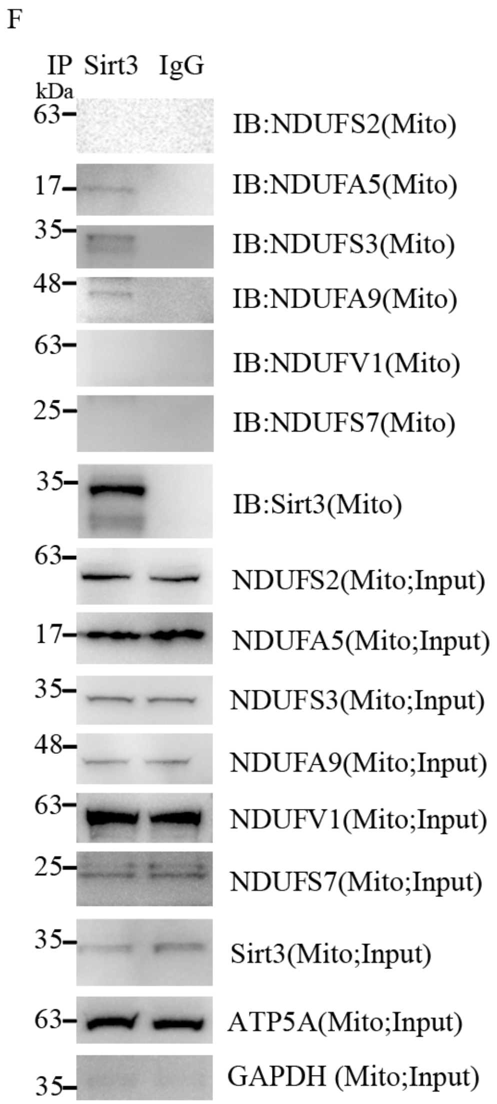

uncropped images

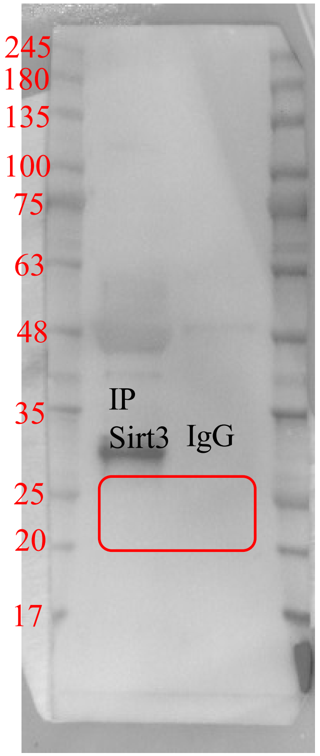

NDUFS7 (Mito)

Figure 1F

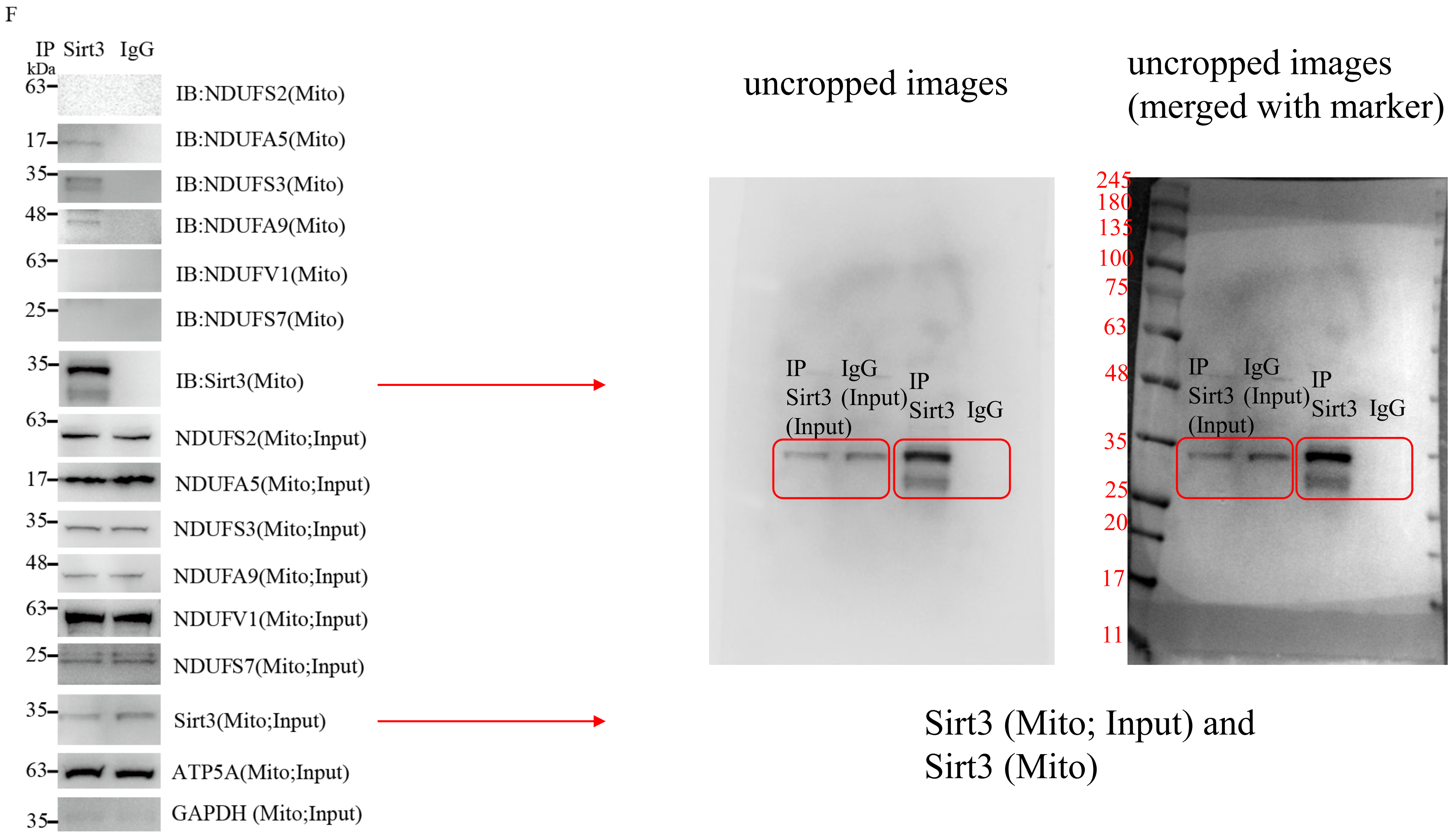
F
IP Sirt3 IgG
kDa
63
IB:NDUFS2(Mito)
17
IB:NDUFA5(Mito)
35
IB:NDUFS3(Mito)
48
IB:NDUFA9(Mito)
63
IB:NDUFV1(Mito)
25
IB:NDUFS7(Mito)
35
IB:Sirt3(Mito)
63
NDUFS2(Mito;Input)
17
NDUFA5(Mito;Input)
35
NDUFS3(Mito;Input)
48
NDUFA9(Mito;Input)
63
NDUFV1(Mito;Input)
25
NDUFS7(Mito;Input)
35
Sirt3(Mito;Input)
63
ATP5A(Mito;Input)
35
GAPDH (Mito;Input)
uncropped images
uncropped images (merged with marker)
IP Sirt3 (Input)
IgG (Input)
IP Sirt3
IgG
245
180
135
100
75
63
48
35
25
20
17
11
Sirt3 (Mito; Input) and Sirt3 (Mito)

Figure 1F

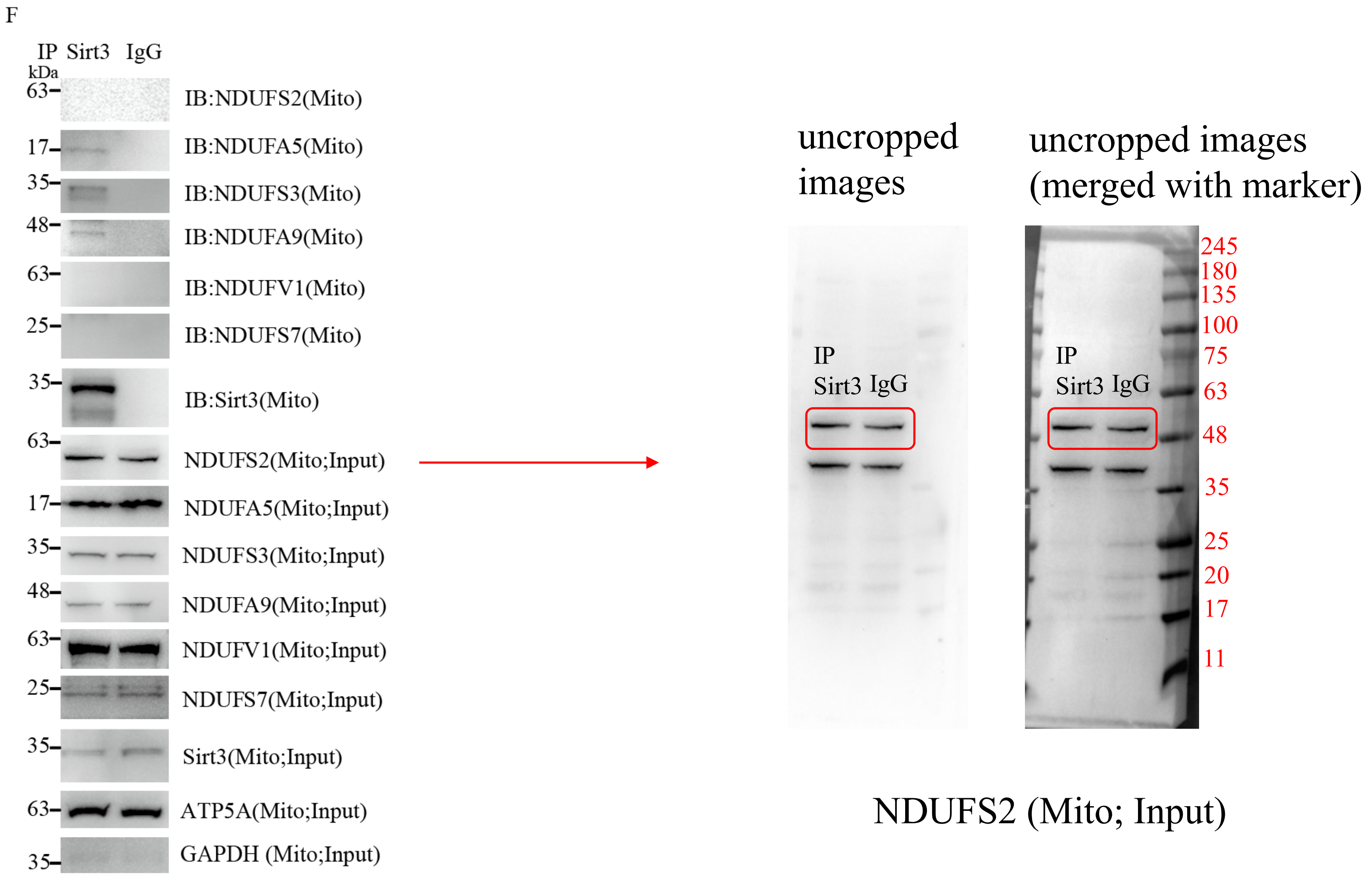
F
IP Sirt3 IgG
kDa
63
IB:NDUFS2(Mito)
17
IB:NDUFA5(Mito)
35
IB:NDUFS3(Mito)
48
IB:NDUFA9(Mito)
63
IB:NDUFV1(Mito)
25
IB:NDUFS7(Mito)
35
IB:Sirt3(Mito)
63
NDUFS2(Mito;Input)
17
NDUFA5(Mito;Input)
35
NDUFS3(Mito;Input)
48
NDUFA9(Mito;Input)
63
NDUFV1(Mito;Input)
25
NDUFS7(Mito;Input)
35
Sirt3(Mito;Input)
63
ATP5A(Mito;Input)
35
GAPDH (Mito;Input)
uncropped images
uncropped images (merged with marker)
IP
Sirt3 IgG
IP
Sirt3 IgG
245
180
135
100
75
63
48
35
25
20
17
11
NDUFS2 (Mito; Input)

Figure 1F

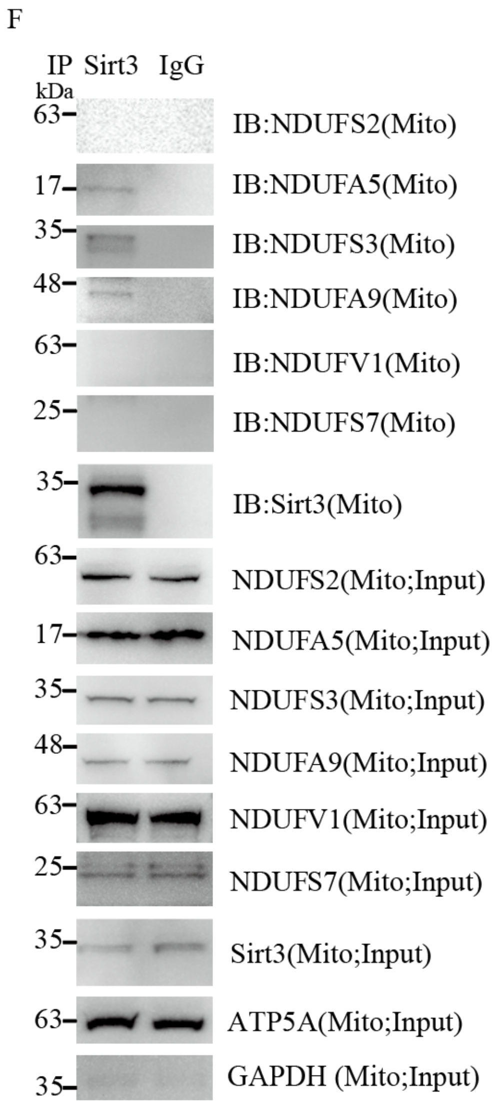

uncropped images

uncropped images (merged with marker)

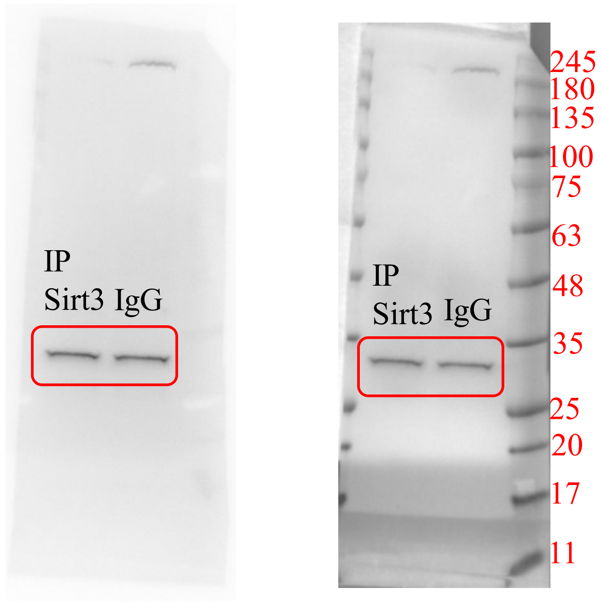

NDUFS3 (Mito; Input)

Figure 1F

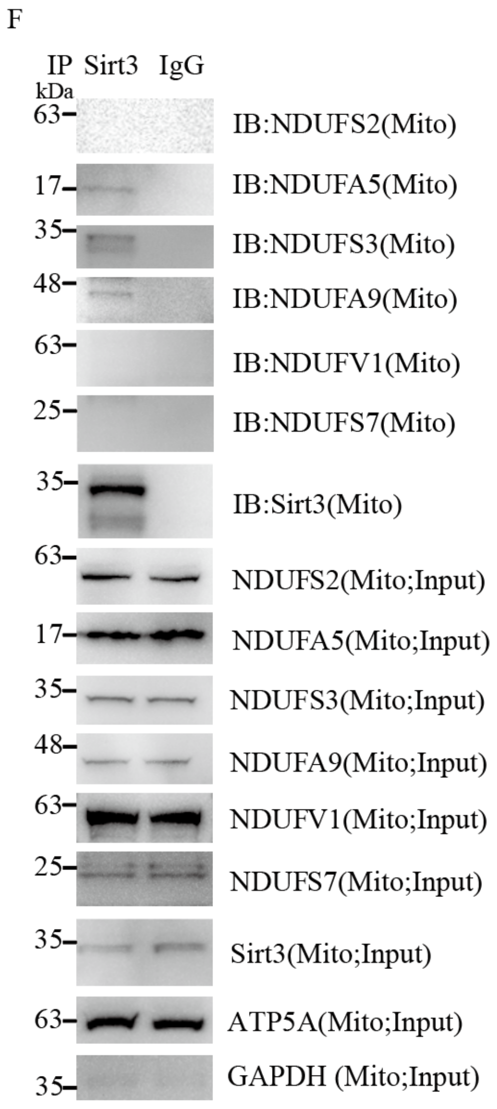

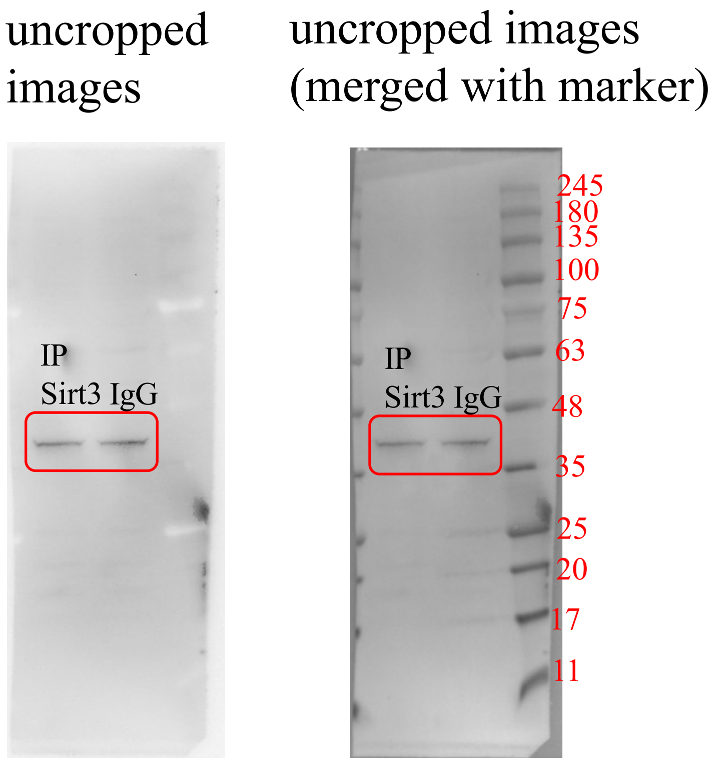

NDUFA9 (Mito; Input)

Figure 1F

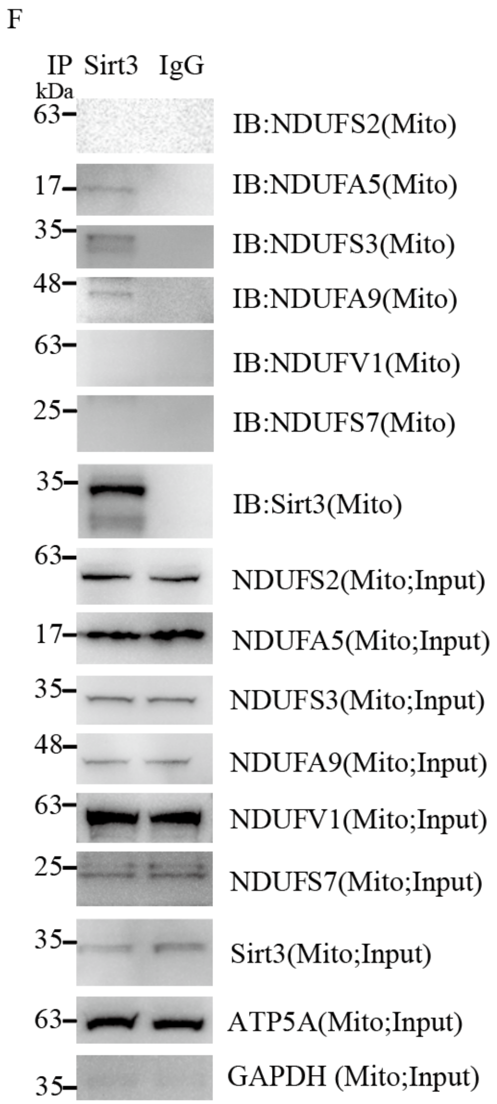

uncropped images

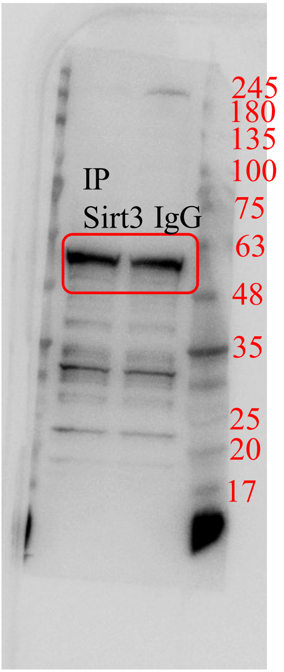

NDUFV1 (Mito; Input)

Figure 1F

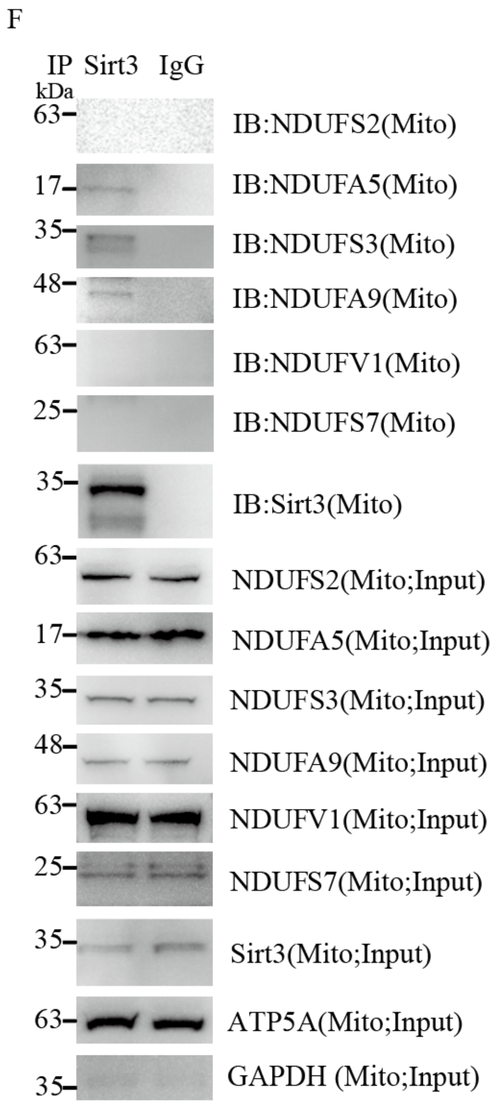

uncropped images

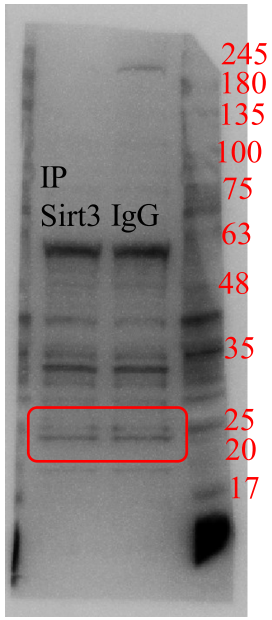

NDUFS7 (Mito; Input)

Figure 1F

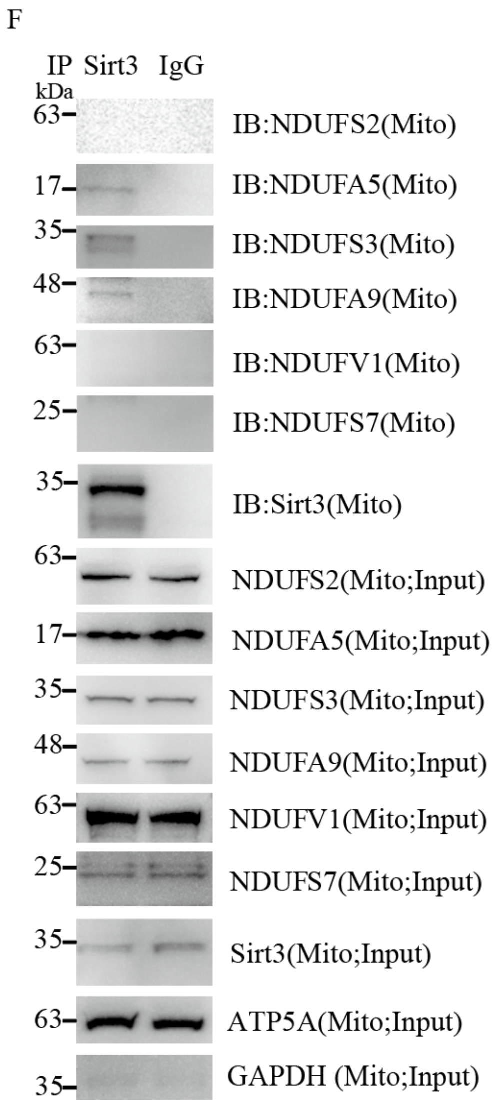

uncropped images

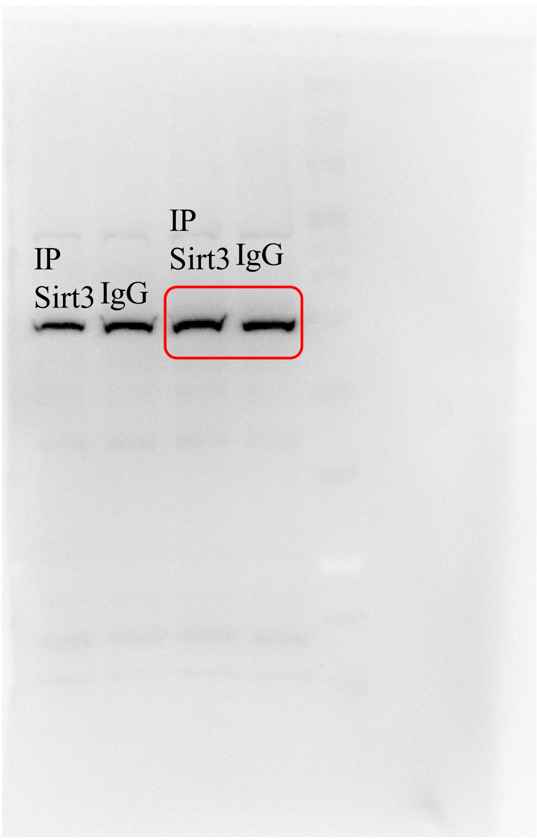

uncropped images (merged with marker)

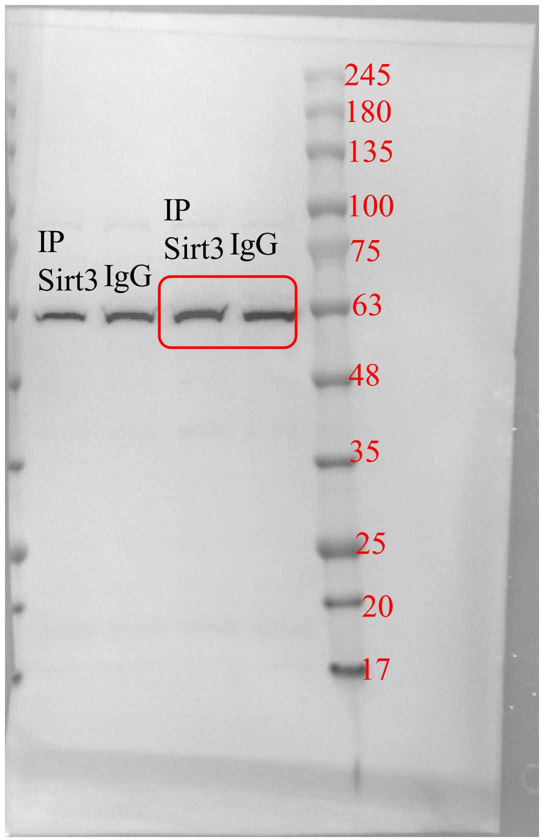

ATP5A (Mito; Input)

Figure 1F

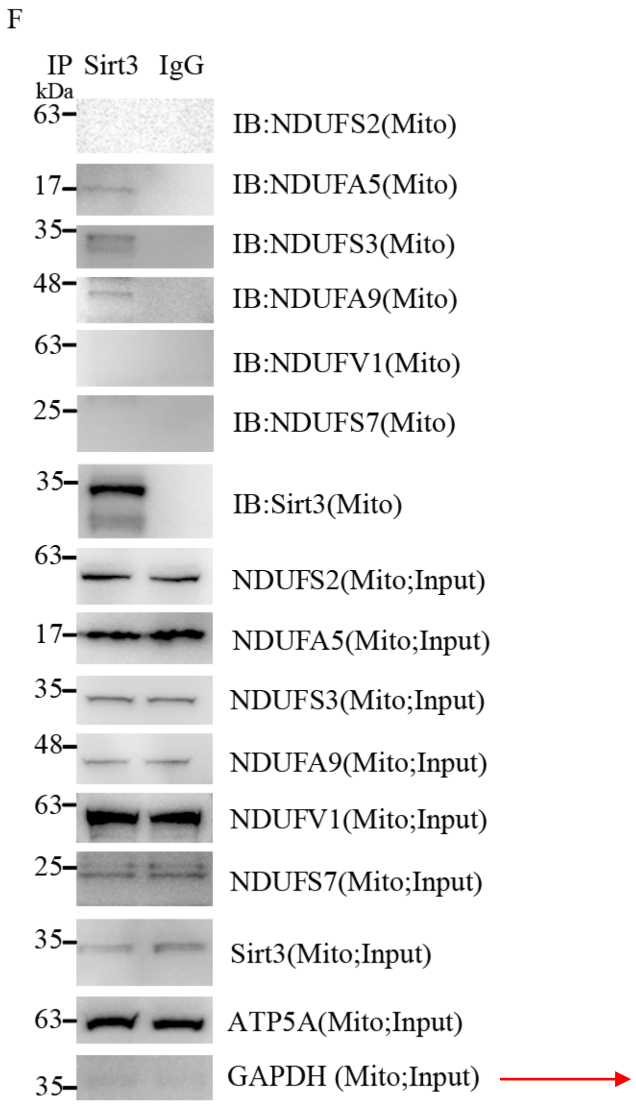

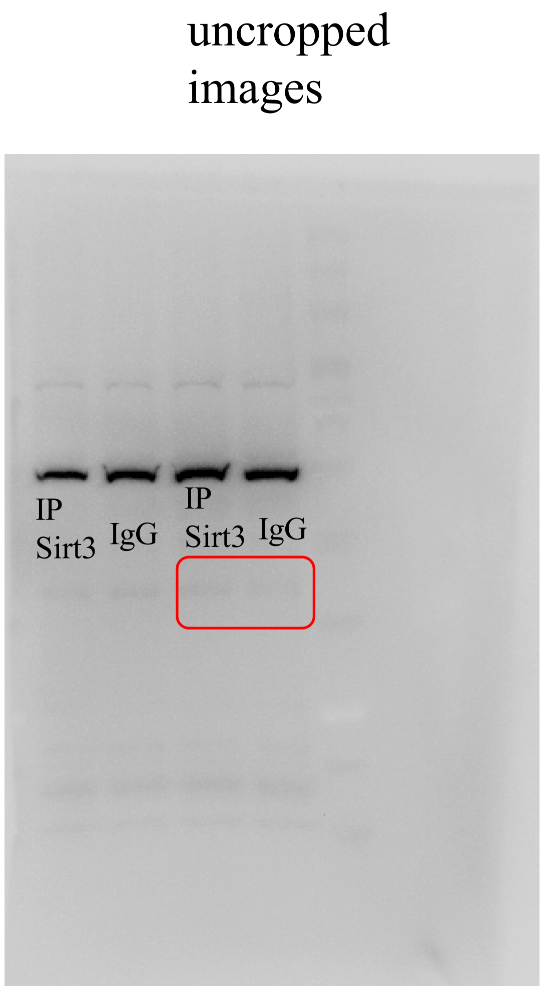

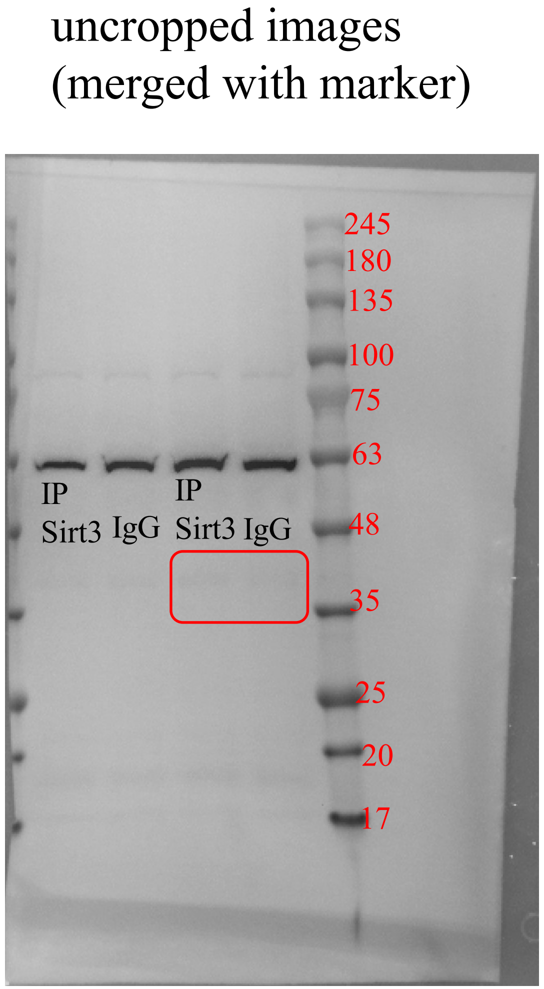

GAPDH (Mito; Input)

Figure 1G

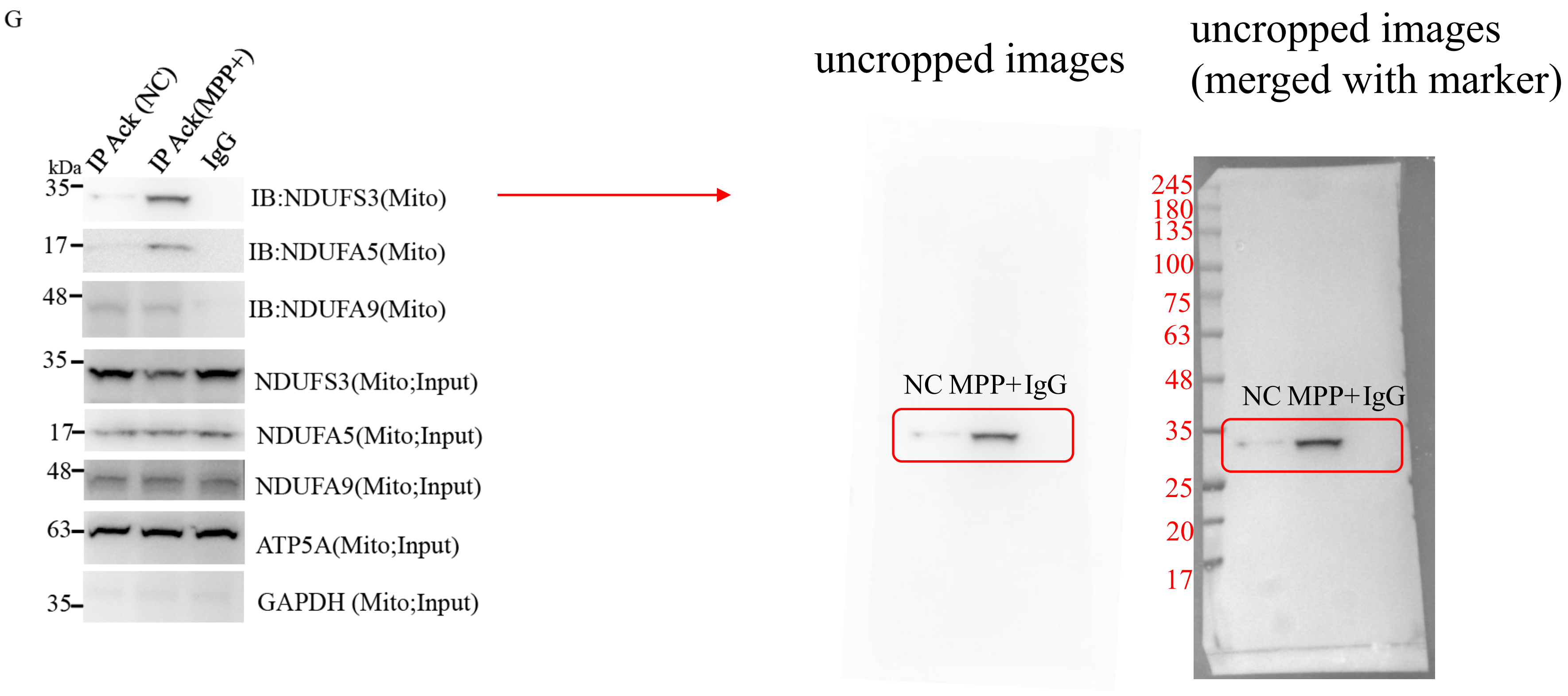

NDUFS3(Mito)

Figure 1G

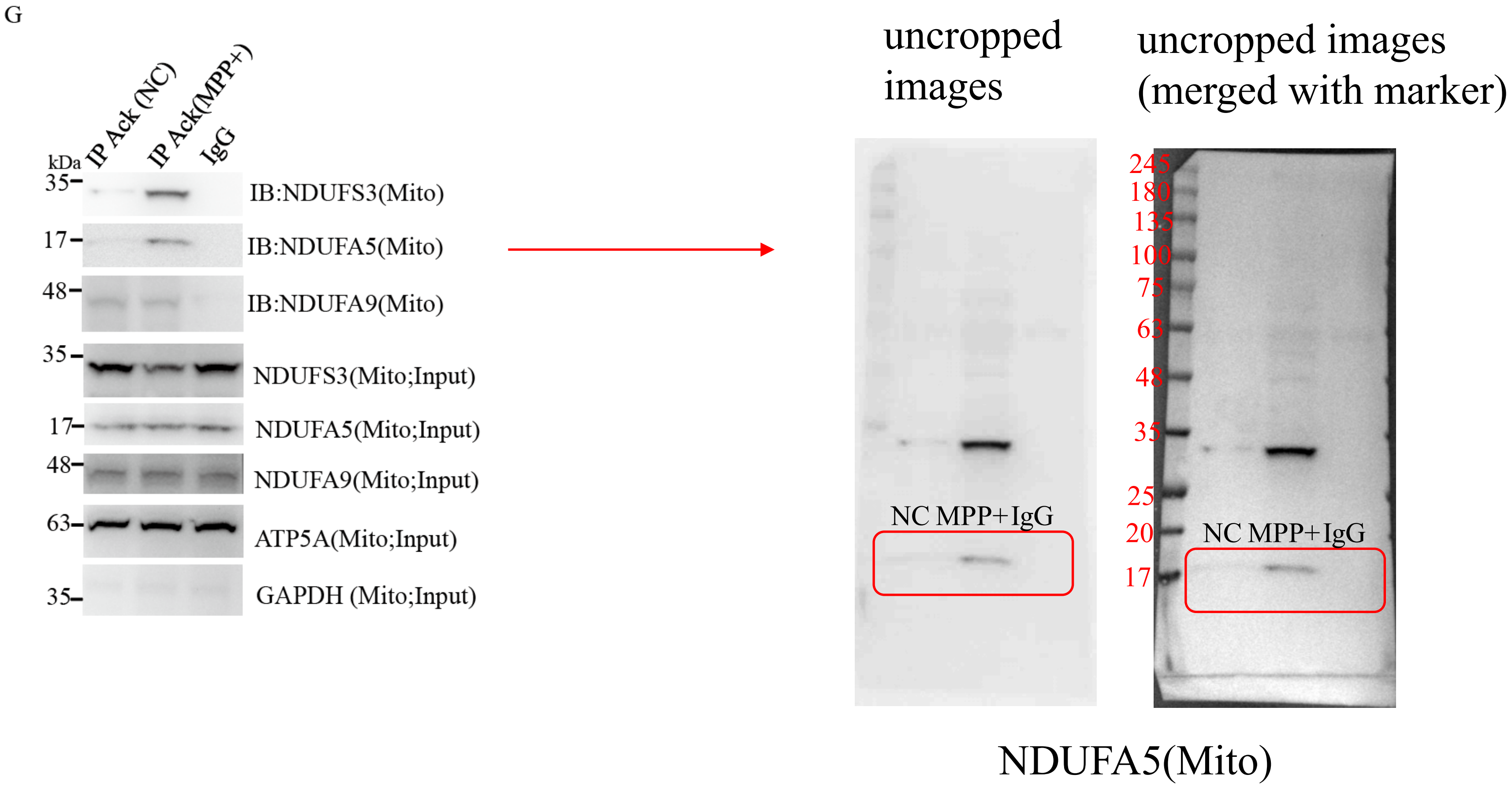
G
kDa
IP Ack (NC)
IP Ack(MPP+)
IgG
35
IB:NDUFS3(Mito)
17
IB:NDUFA5(Mito)
48
IB:NDUFA9(Mito)
35
NDUFS3(Mito;Input)
17
NDUFA5(Mito;Input)
48
NDUFA9(Mito;Input)
63
ATP5A(Mito;Input)
35
GAPDH (Mito;Input)
uncropped images
uncropped images (merged with marker)
245
180
135
100
75
63
48
35
25
20
17
NC MPP+IgG
NC MPP+IgG
NDUFA5(Mito)

Figure 1G

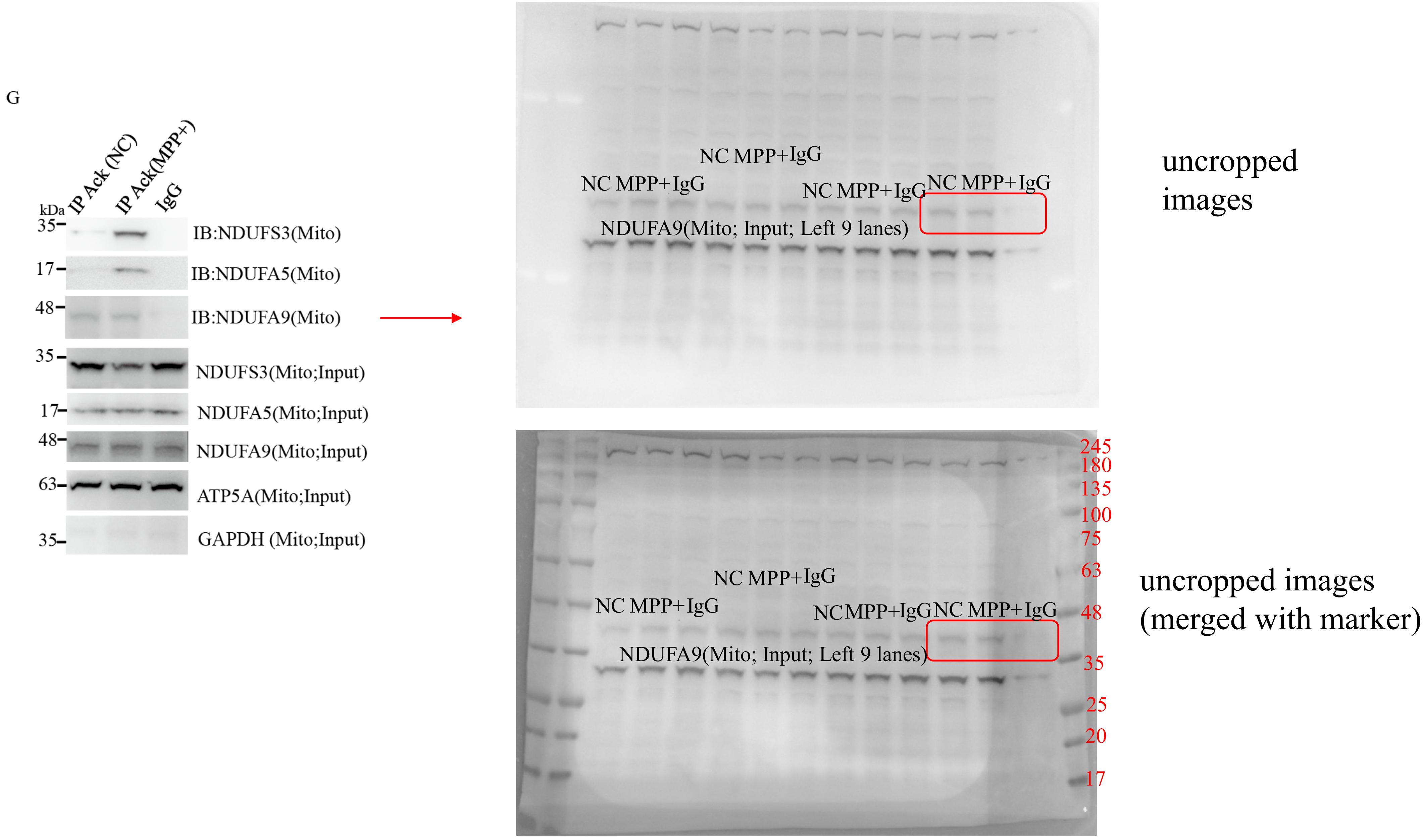

NDUFA9(Mito; Lanes 10th to 12th)

Figure 1G

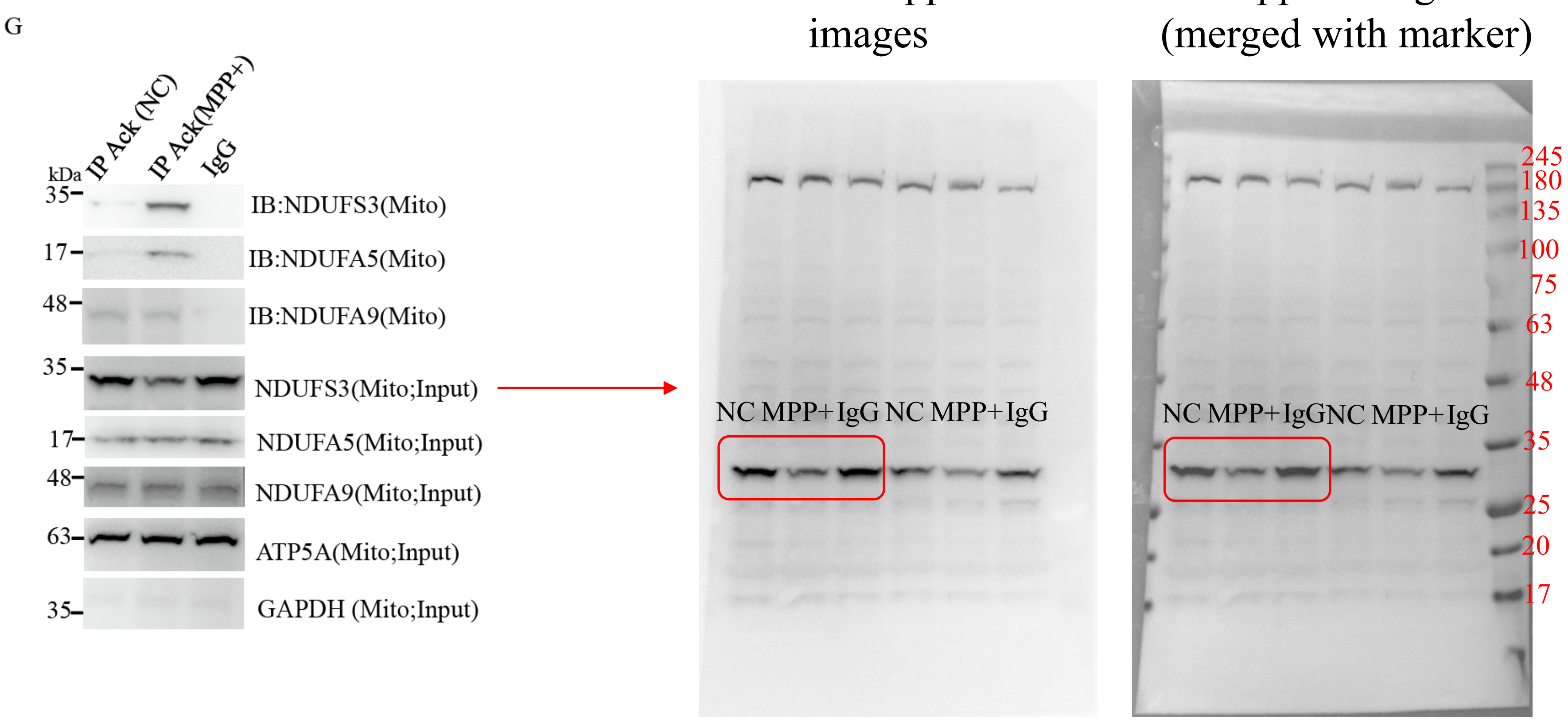

NDUFS3 (Mito; Input)

Figure 1G

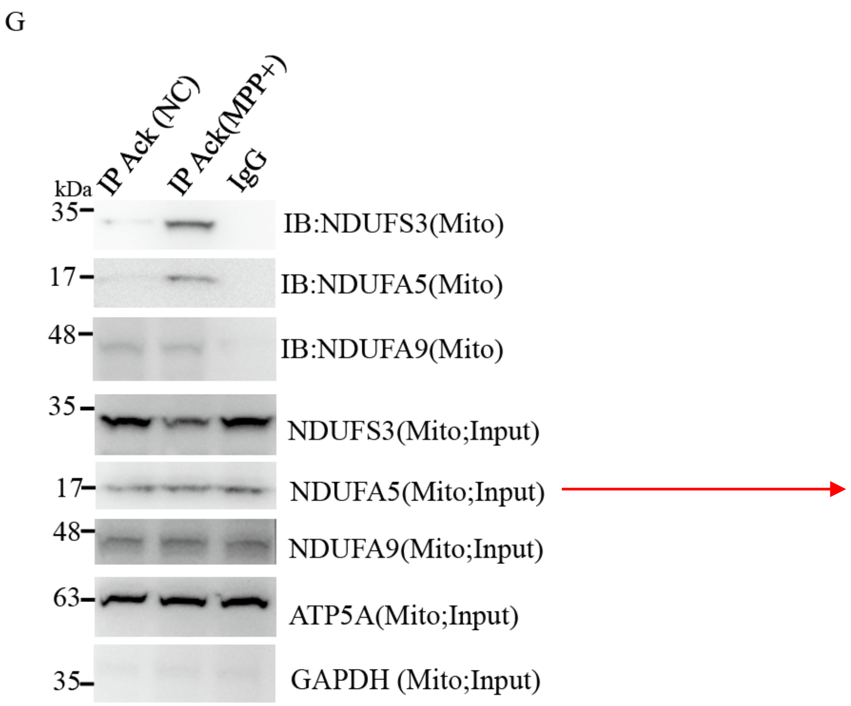

uncropped images

uncropped images (merged with marker)

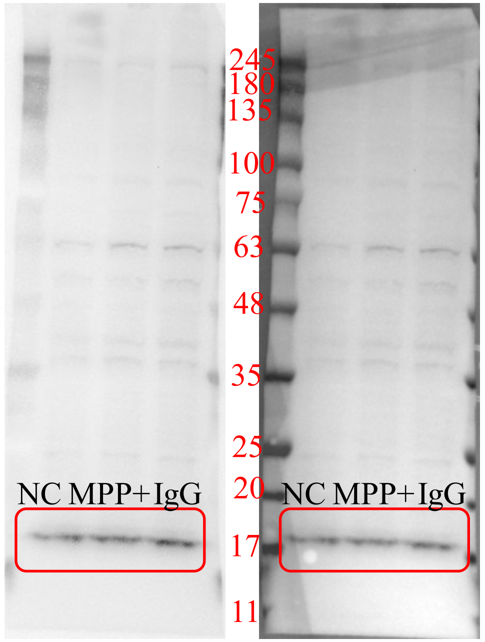

NDUFA5 (Mito; Input)

Figure 1G

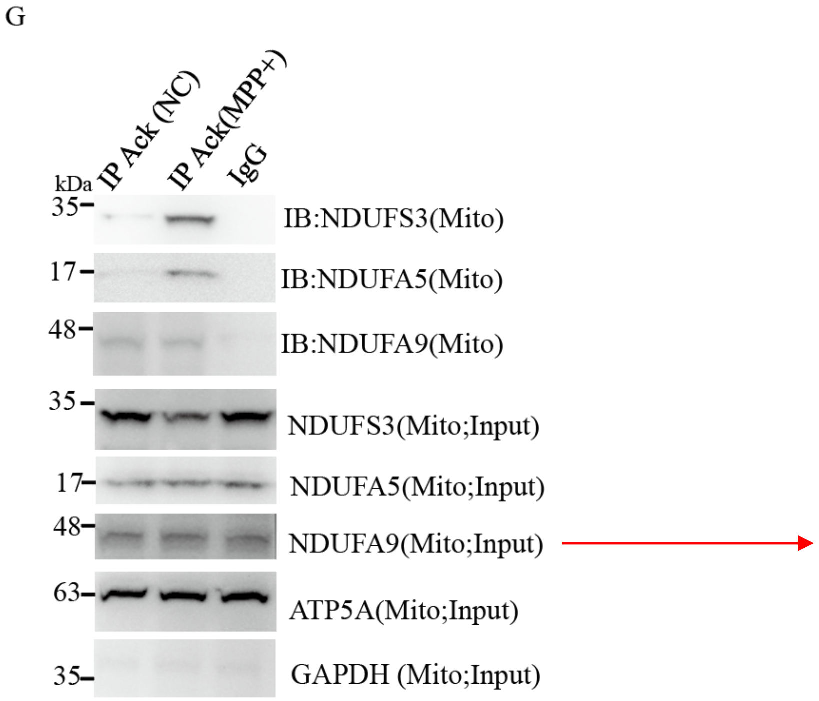

uncropped images

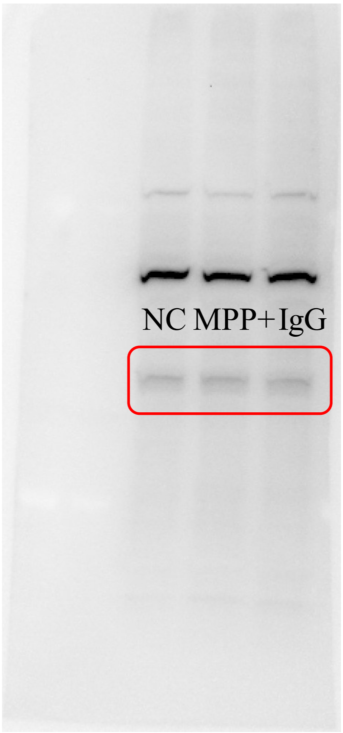

uncropped images
(merged with marker)

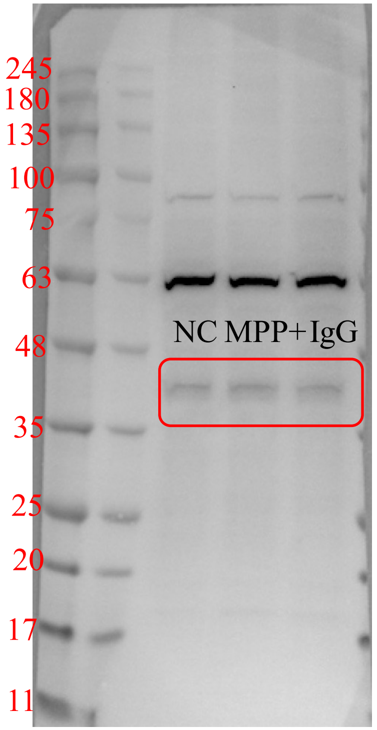

NDUFA9 (Mito; Input)

Figure 1G

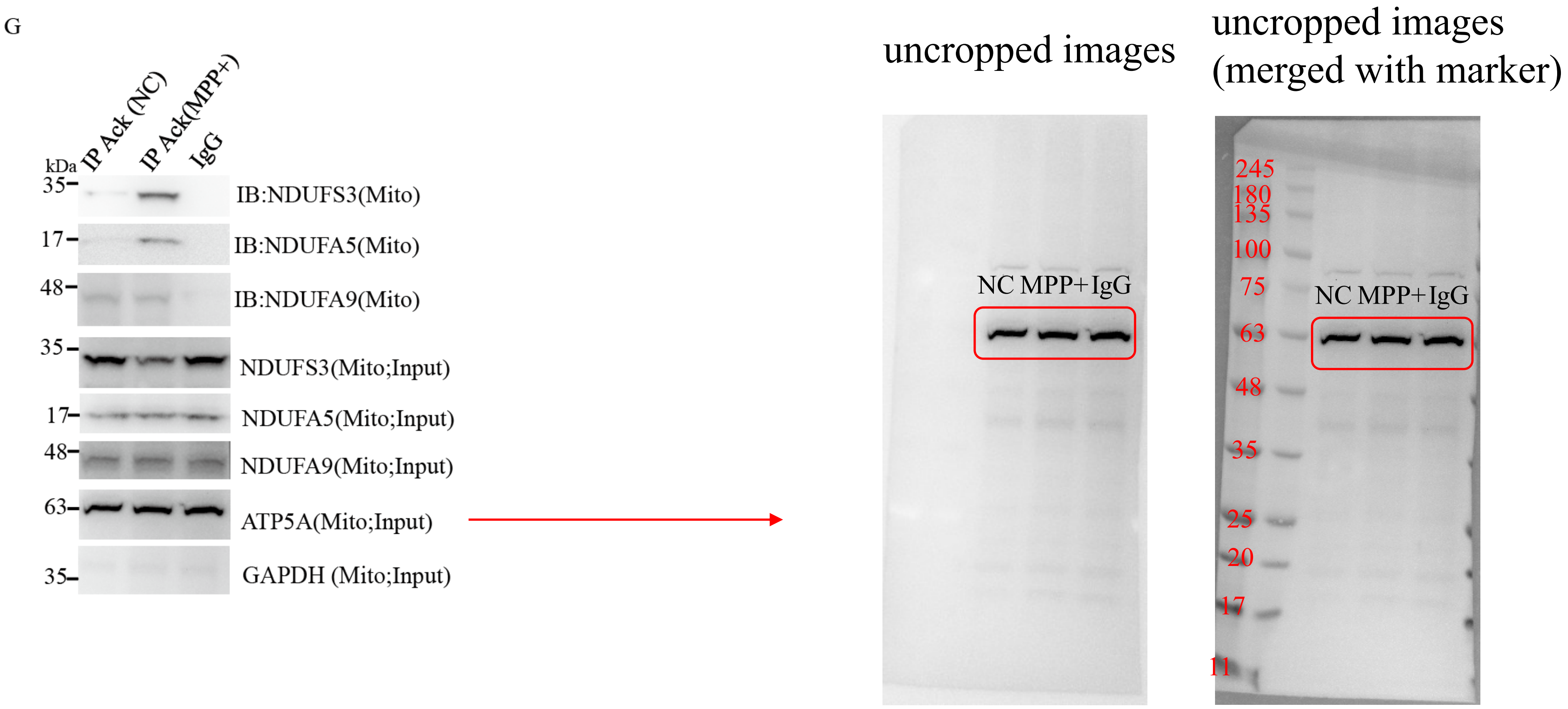

ATP5A(Mito; Input)

Figure 1G

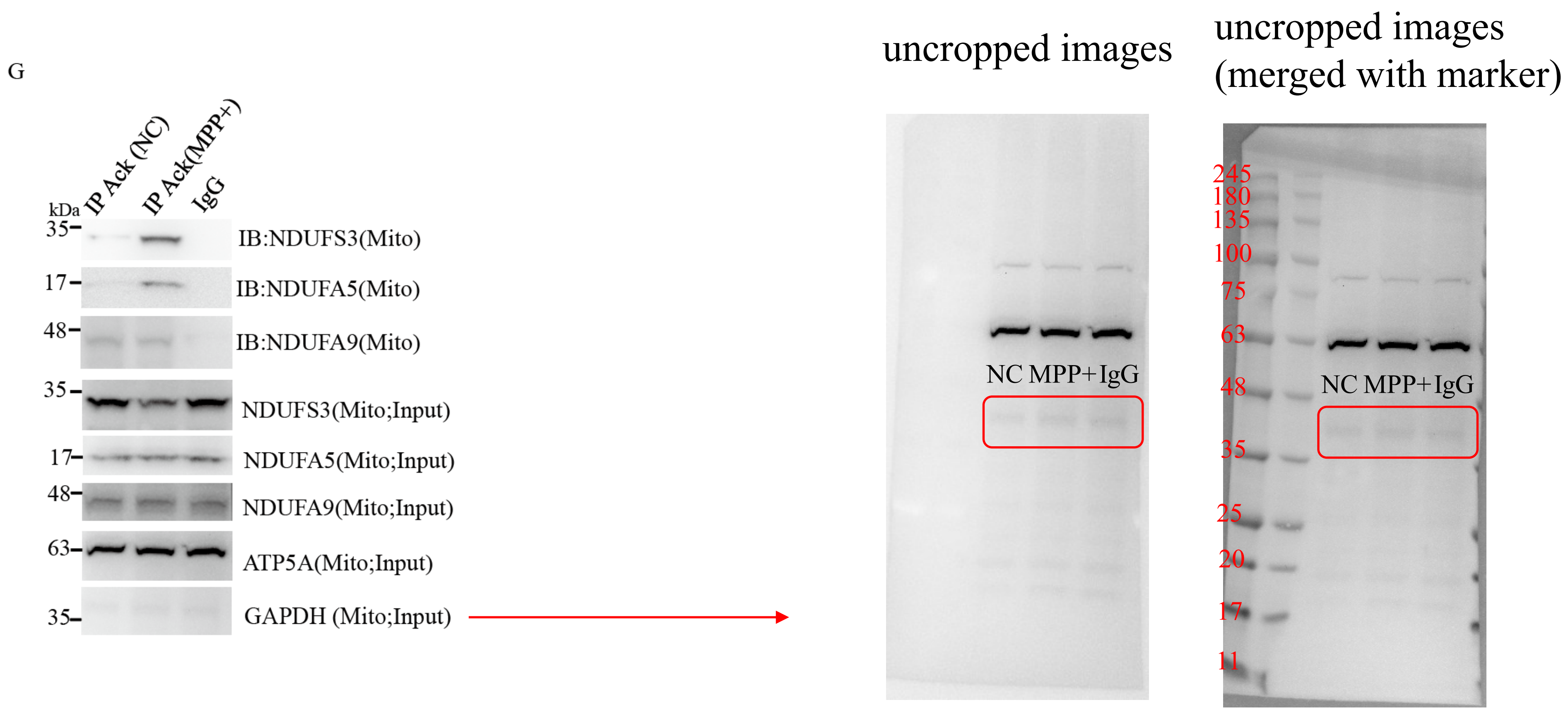

GAPDH (Mito; Input)

Figure 2A

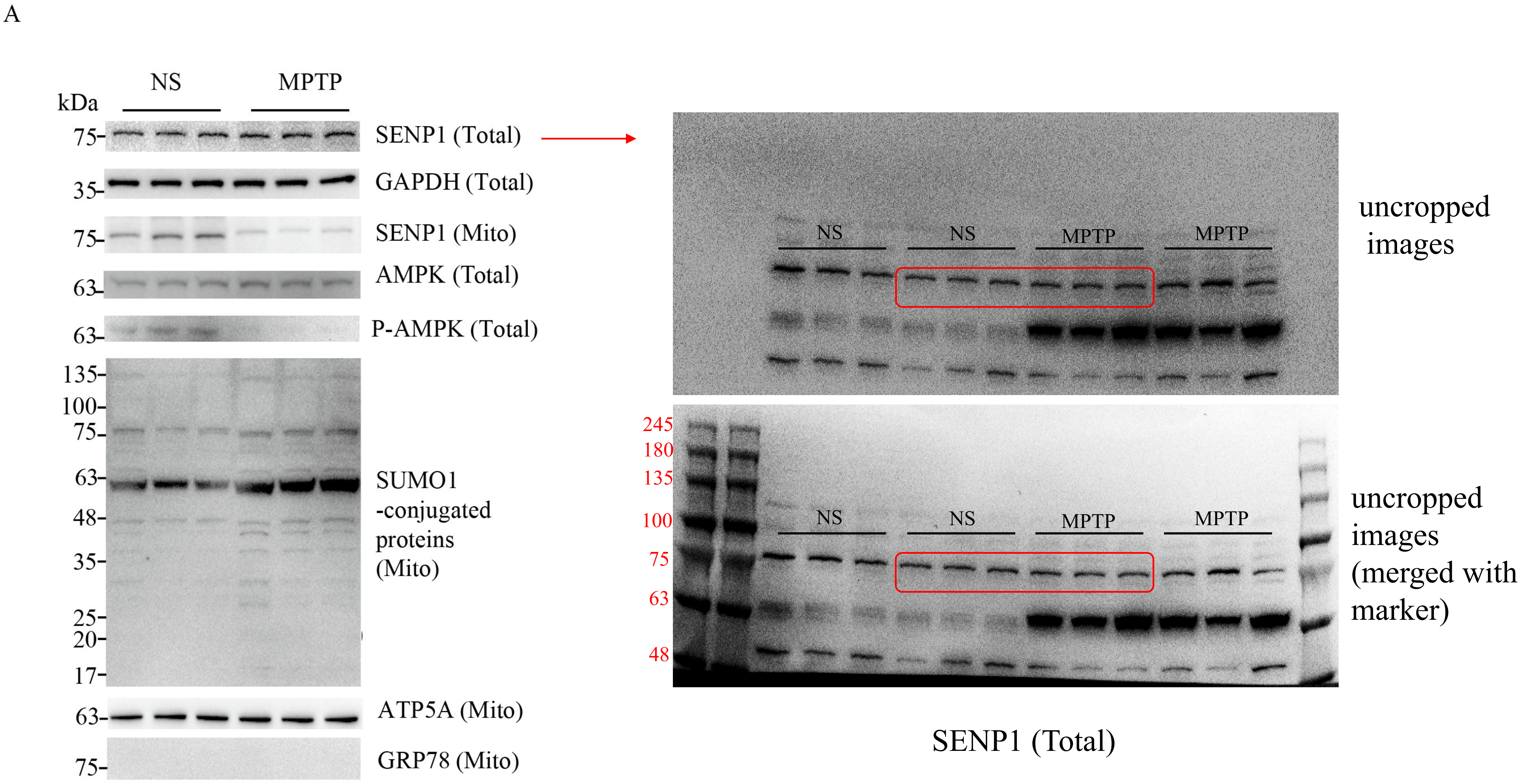
A
NS
MPTP
kDa
75-
SENP1 (Total)
35-
GAPDH (Total)
75-
SENP1 (Mito)
63-
AMPK (Total)
63-
P-AMPK (Total)
135-
100-
75-
63-
48-
35-
25-
20-
17-
SUMO1
-conjugated
proteins
(Mito)
63-
ATP5A (Mito)
75-
GRP78 (Mito)
NS
NS
MPTP
MPTP
uncropped
images
245
180
135
100
75
63
48
NS
NS
MPTP
MPTP
uncropped
images
(merged with
marker)
SENP1 (Total)

Figure 2A

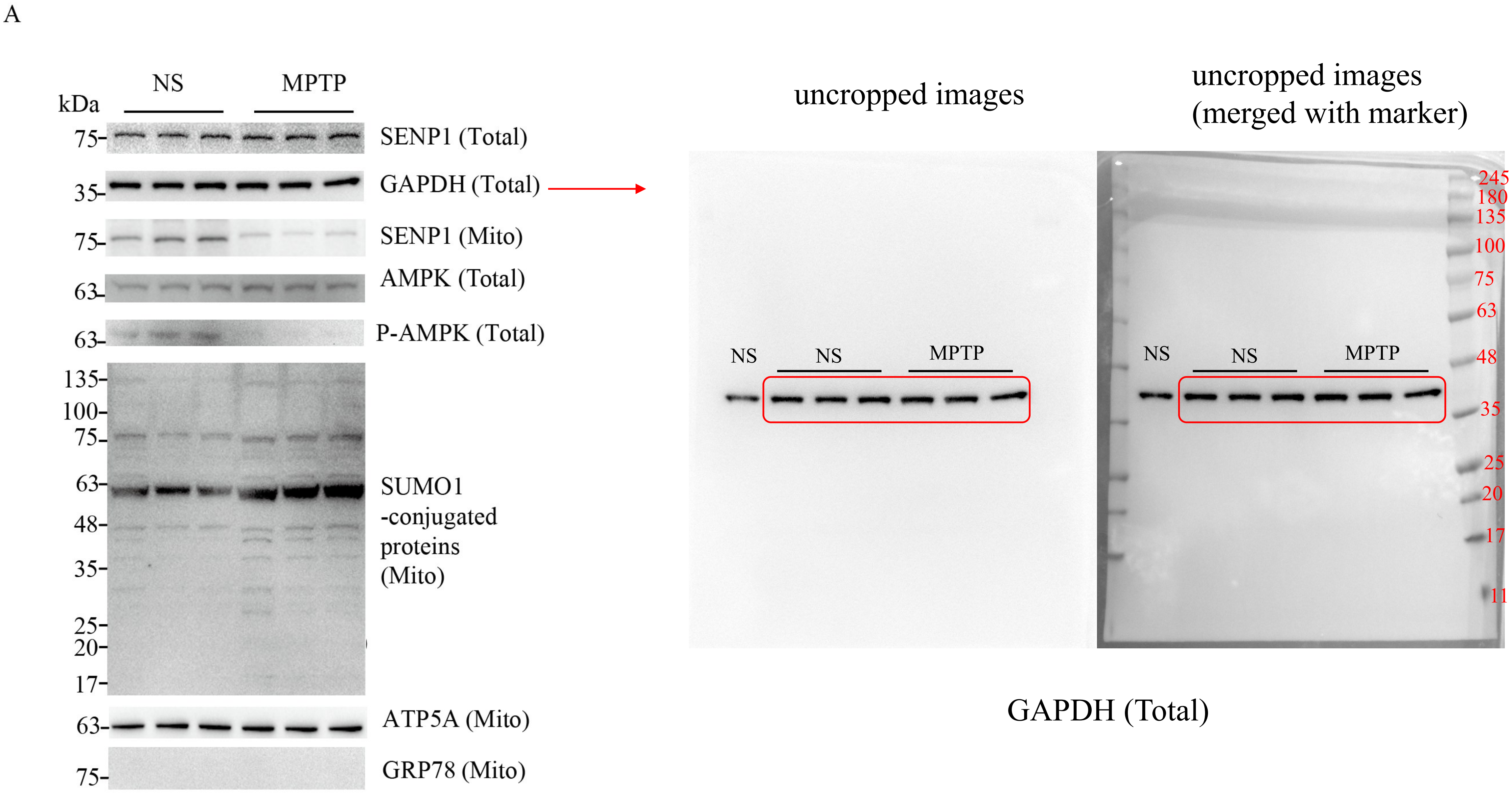

GAPDH (Total)

Figure 2A

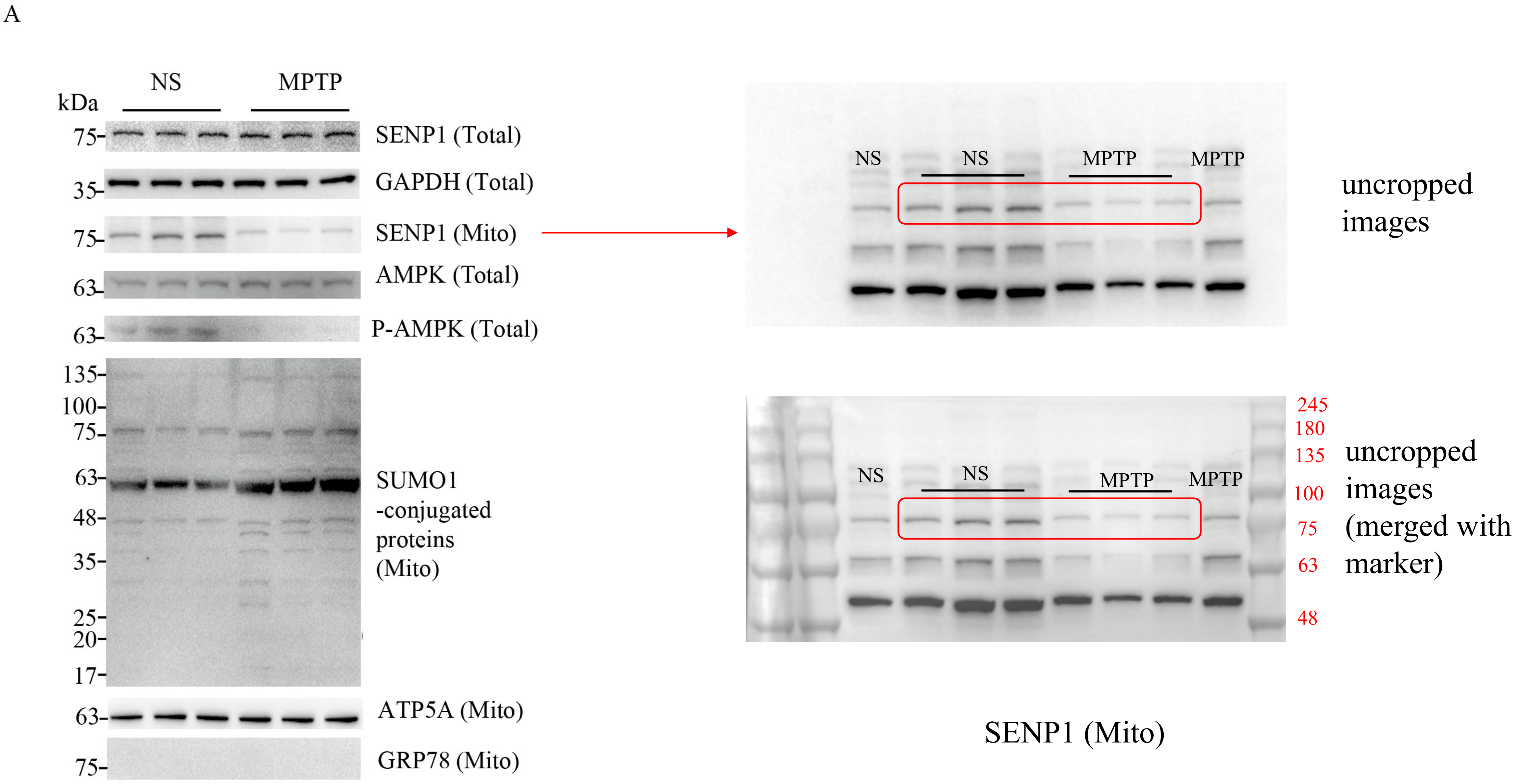

A
kDa
NS
MPTP
75-
SENP1 (Total)
35-
GAPDH (Total)
75-
SENP1 (Mito)
63-
AMPK (Total)
63-
P-AMPK (Total)
135-
100-
75-
63-
48-
35-
25-
20-
17-
SUMO1
-conjugated
proteins
(Mito)
63-
ATP5A (Mito)
75-
GRP78 (Mito)
NS
NS
MPTP
MPTP
uncropped
images
245
180
135
100
75
63
48
uncropped
images
(merged with
marker)
SENP1 (Mito)

Figure 2A

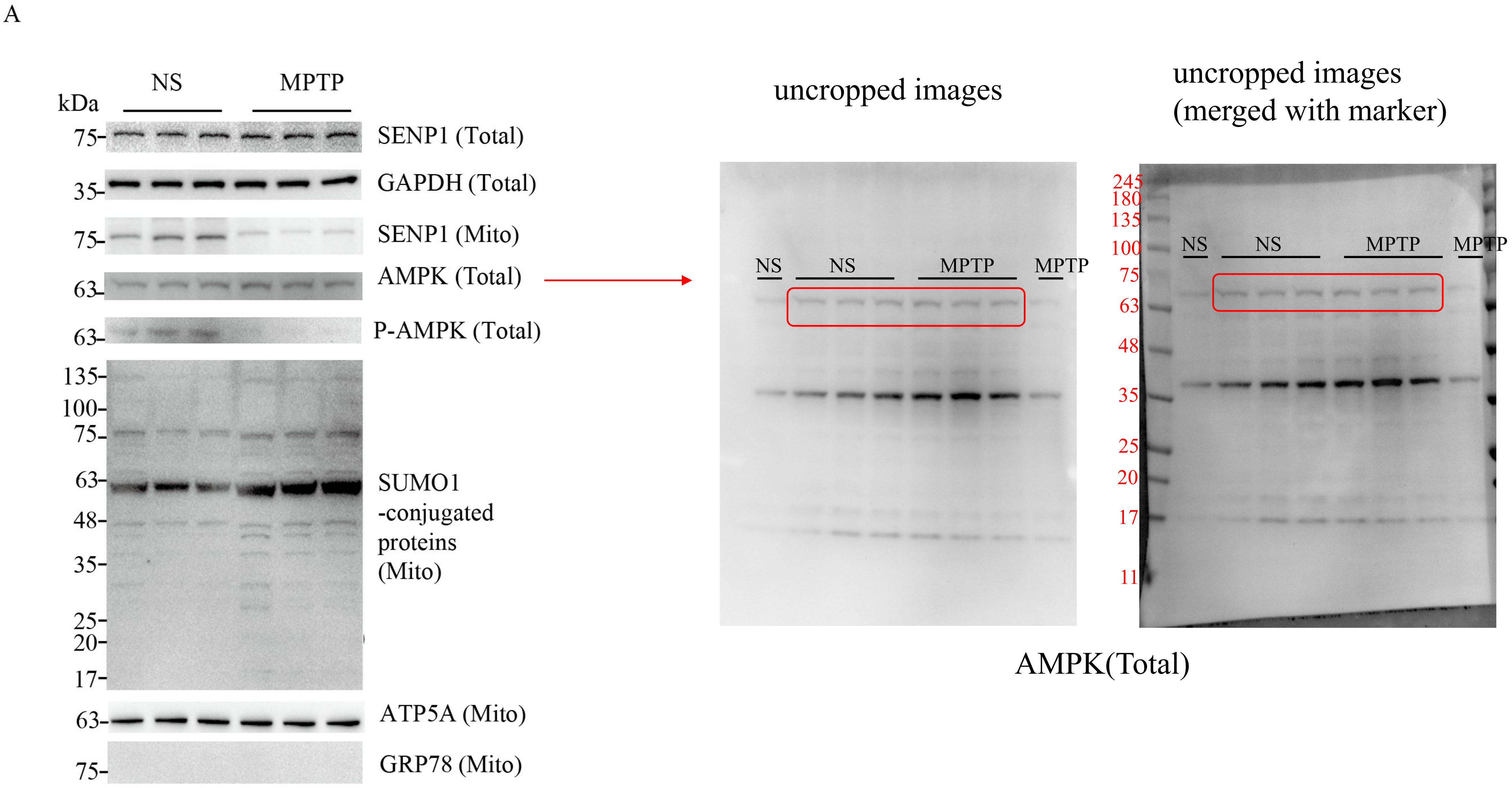

AMPK(Total)

Figure 2A

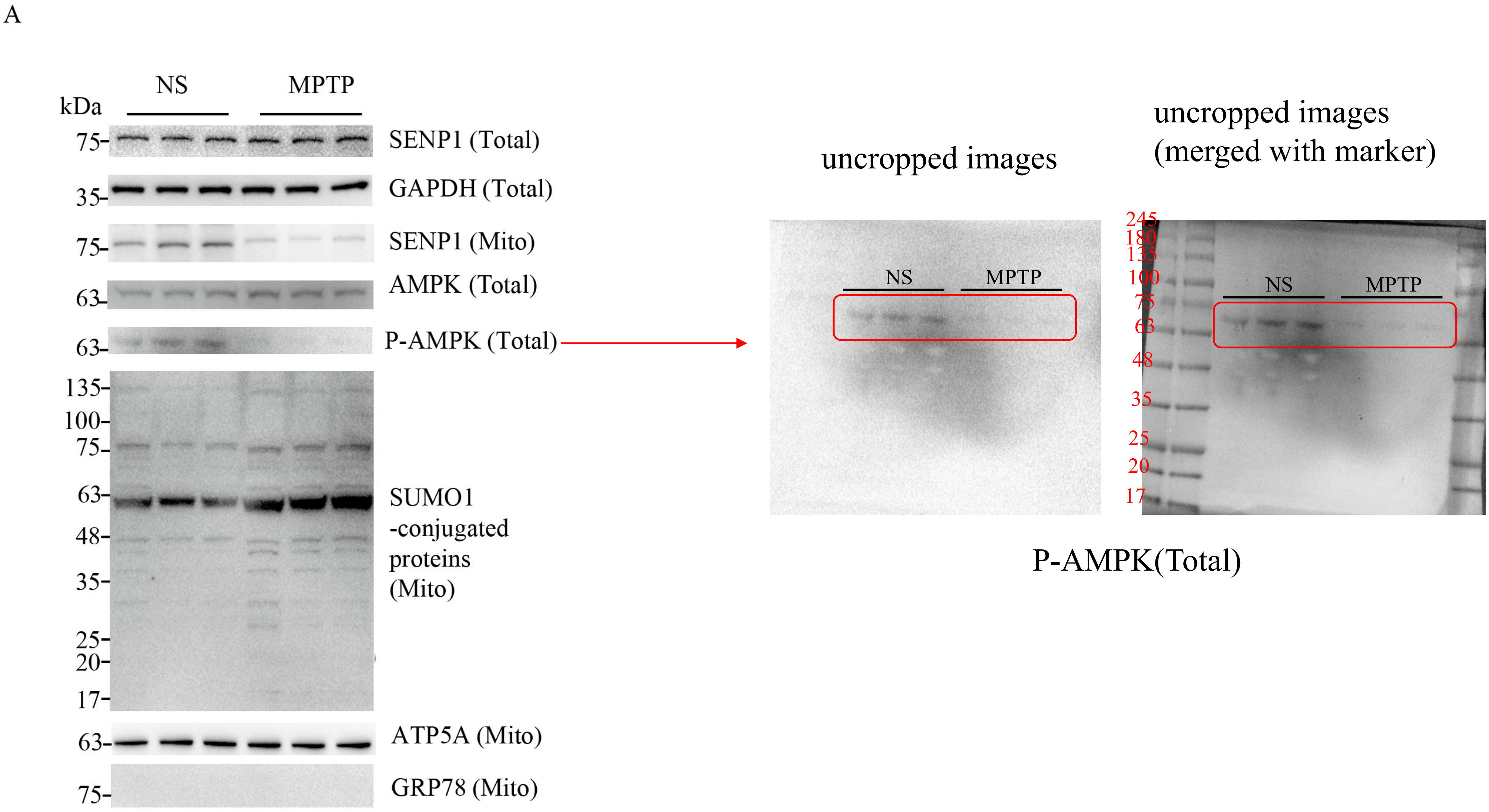

P-AMPK(Total)

Figure 2A

A

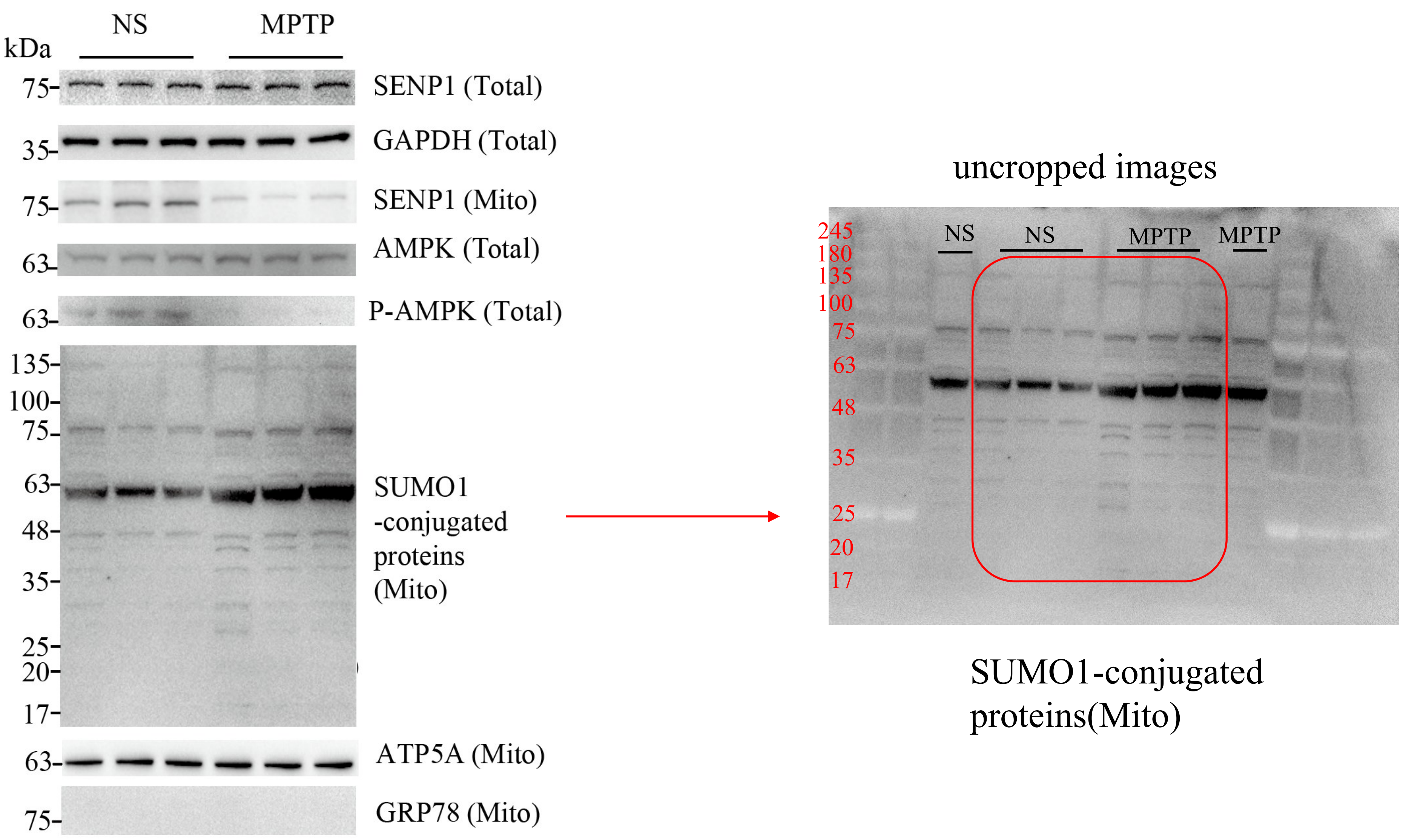

NS
MPTP
kDa
75
SENP1 (Total)
35
GAPDH (Total)
75
SENP1 (Mito)
63
AMPK (Total)
63
P-AMPK (Total)
135
100
75
63
48
35
25
20
17
SUMO1
-conjugated
proteins
(Mito)
63
ATP5A (Mito)
75
GRP78 (Mito)
uncropped images
245
180
135
100
75
63
48
35
25
20
17
NS
NS
MPTP
MPTP
SUMO1-conjugated
proteins(Mito)

Figure 2A

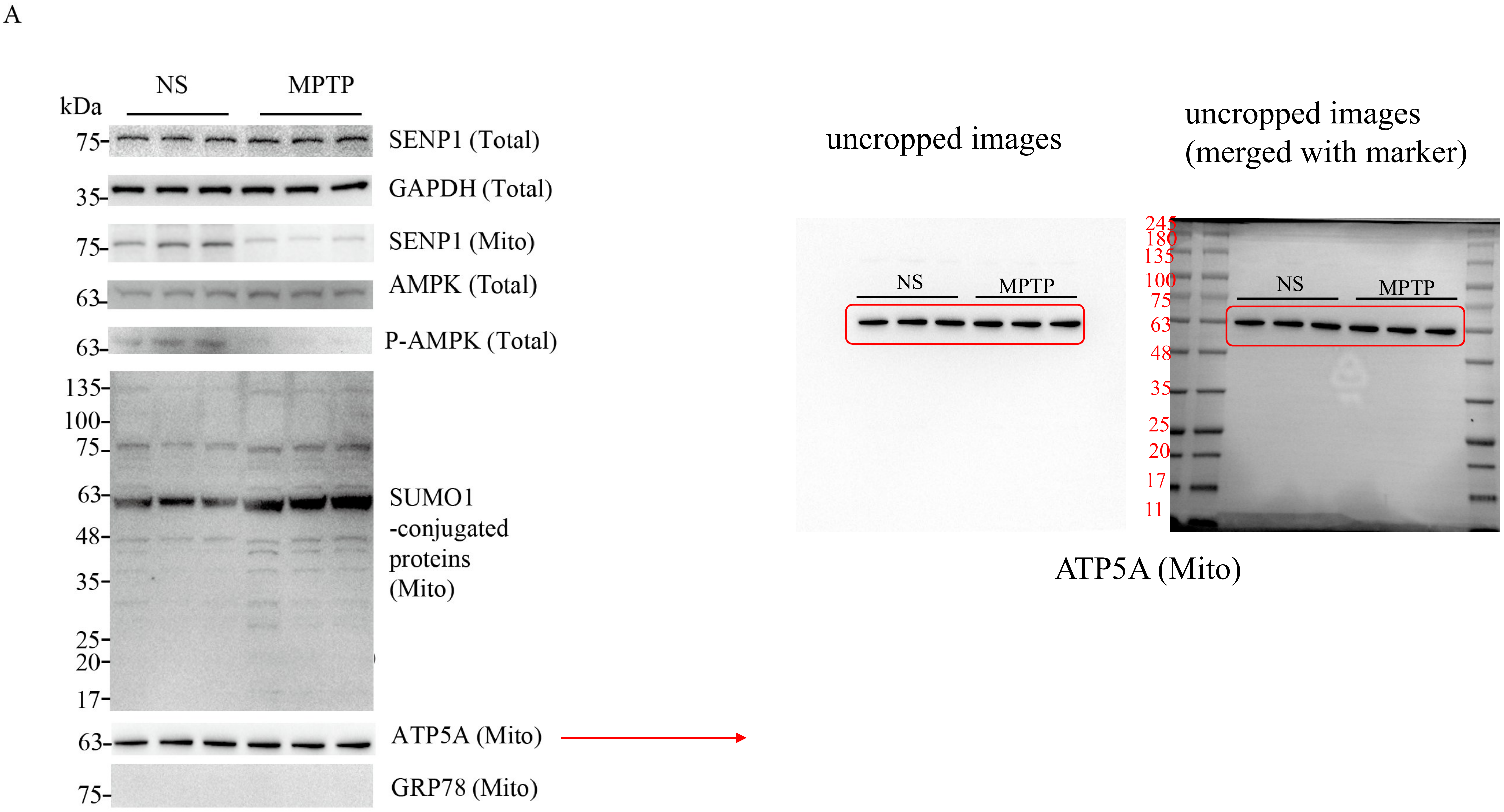
A
kDa
NS
MPTP
75-
SENP1 (Total)
35-
GAPDH (Total)
75-
SENP1 (Mito)
63-
AMPK (Total)
63-
P-AMPK (Total)
135-
100-
75-
63-
48-
35-
25-
20-
17-
SUMO1 -conjugated proteins (Mito)
63-
ATP5A (Mito)
75-
GRP78 (Mito)
uncropped images
uncropped images (merged with marker)
NS
MPTP
245
180
135
100
75
63
48
35
25
20
17
11
ATP5A (Mito)

Figure 2A

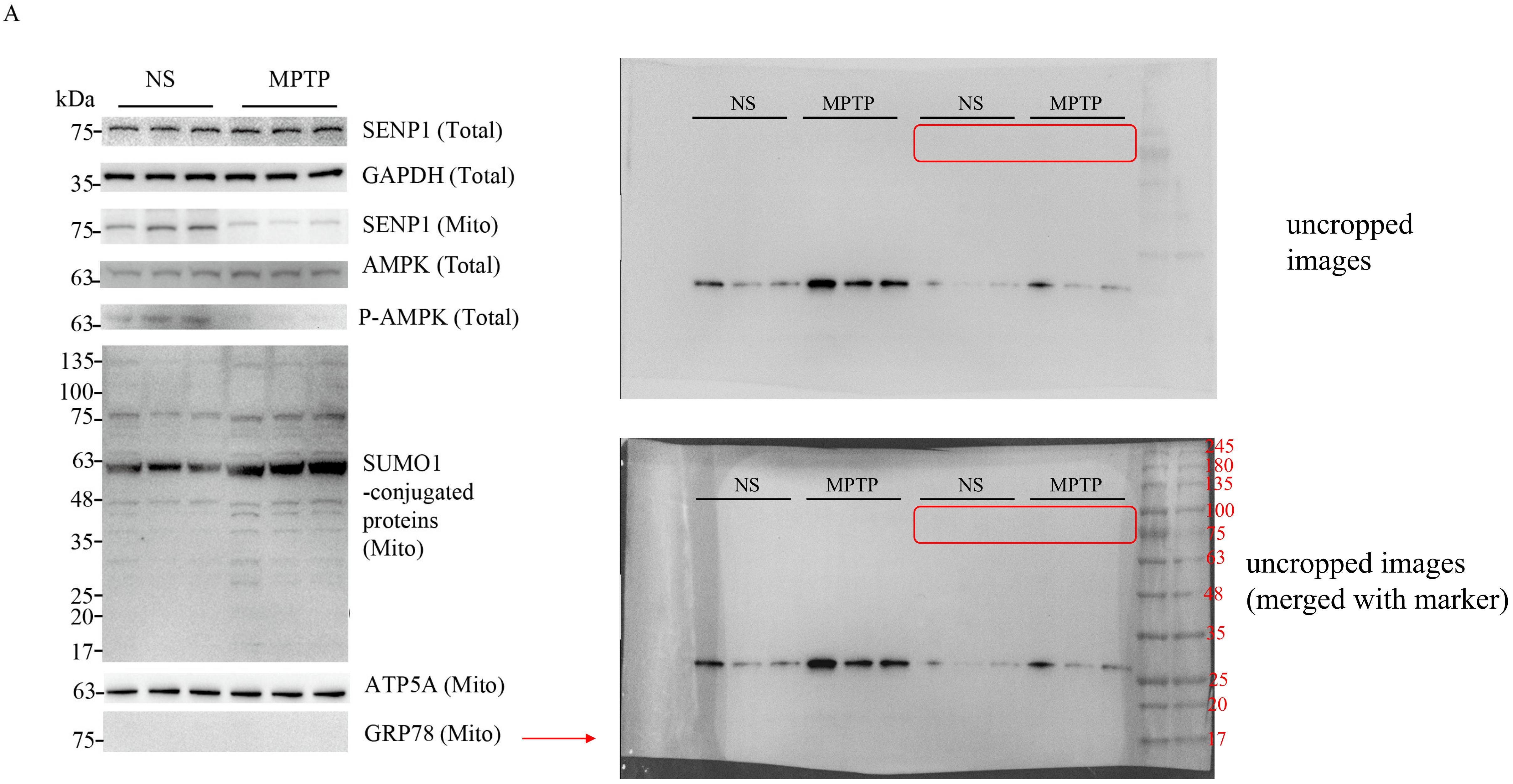

GRP78 (Mito)

Figure 2B

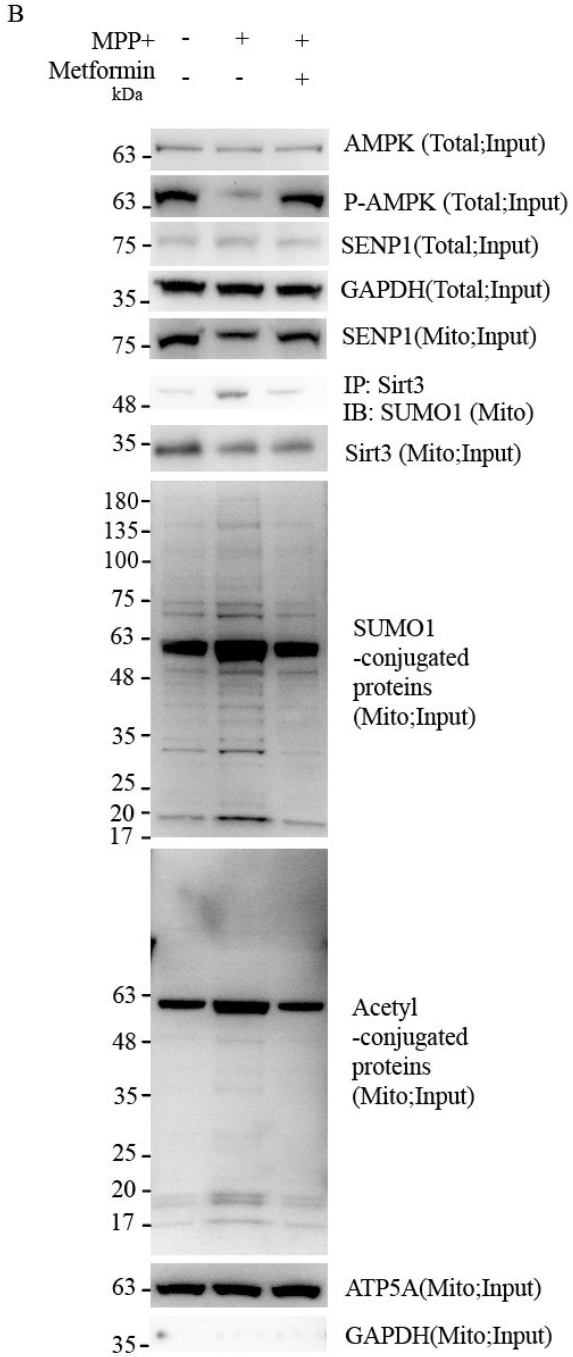

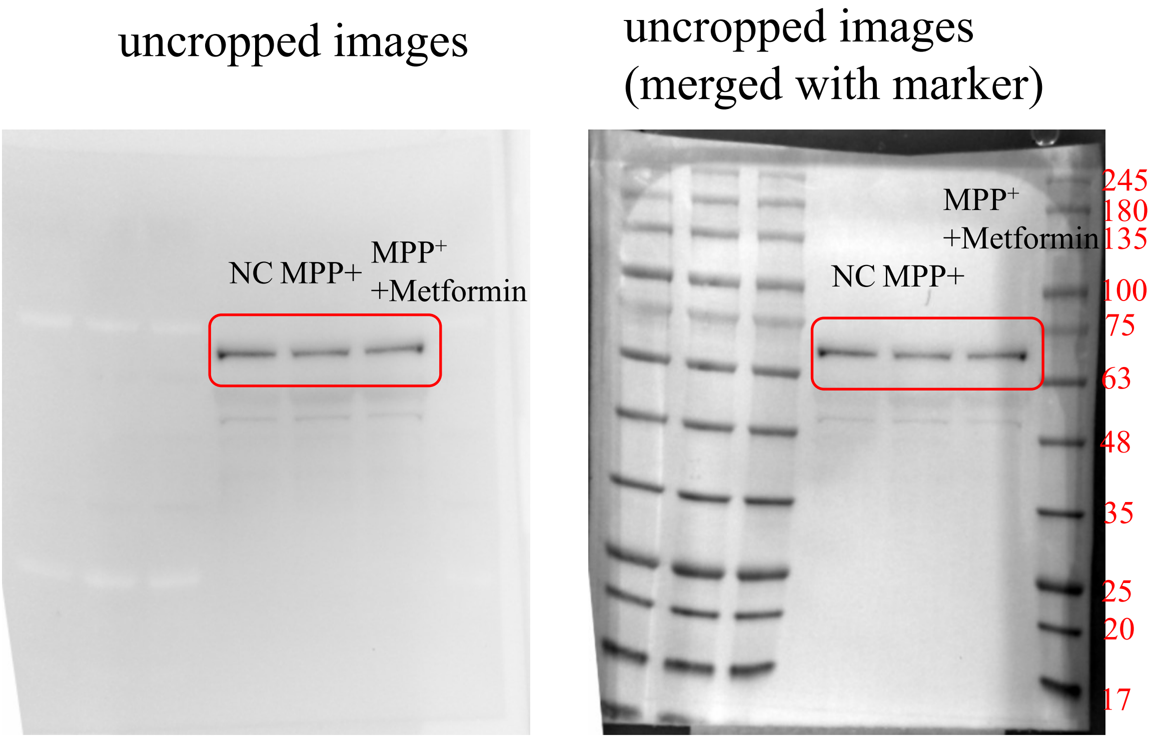

AMPK (Total; Input)

Figure 2B

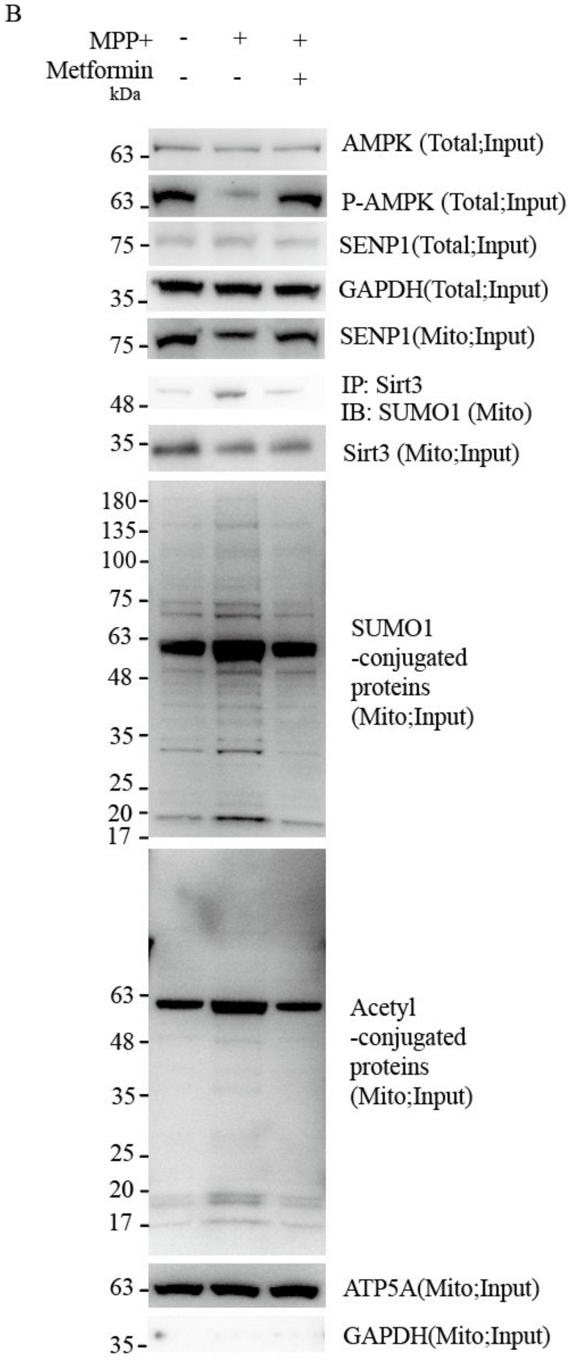

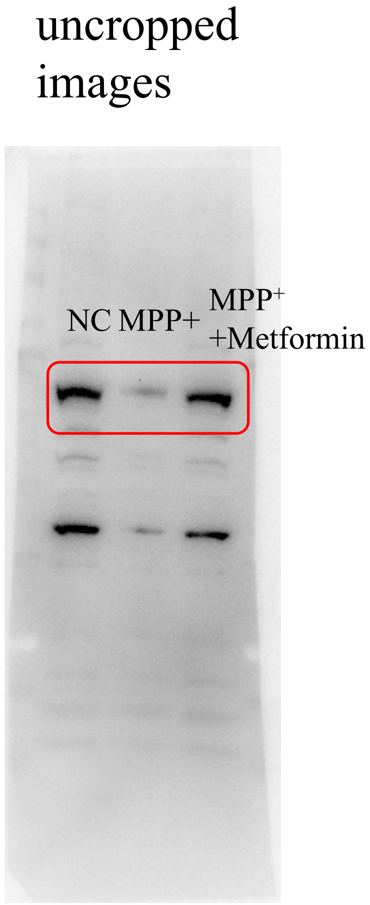

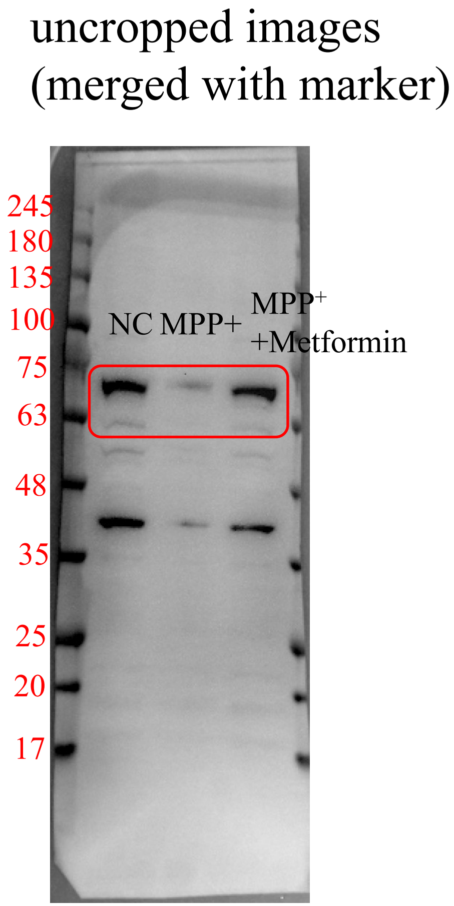

P-AMPK(Total; Input)

Figure 2B

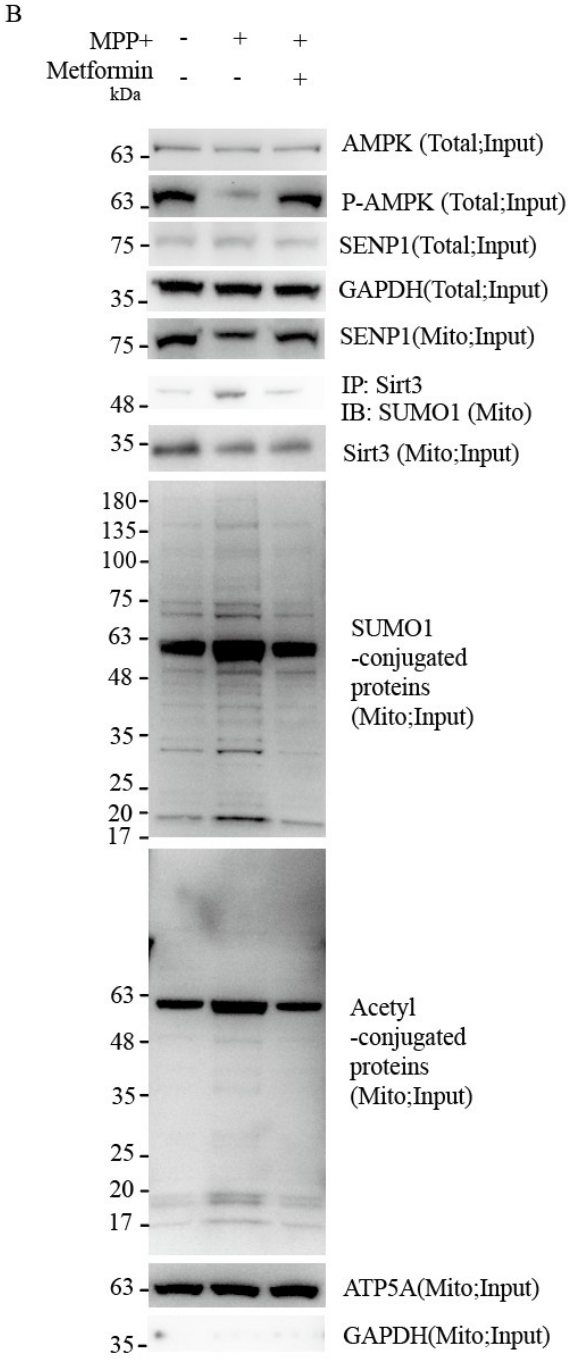

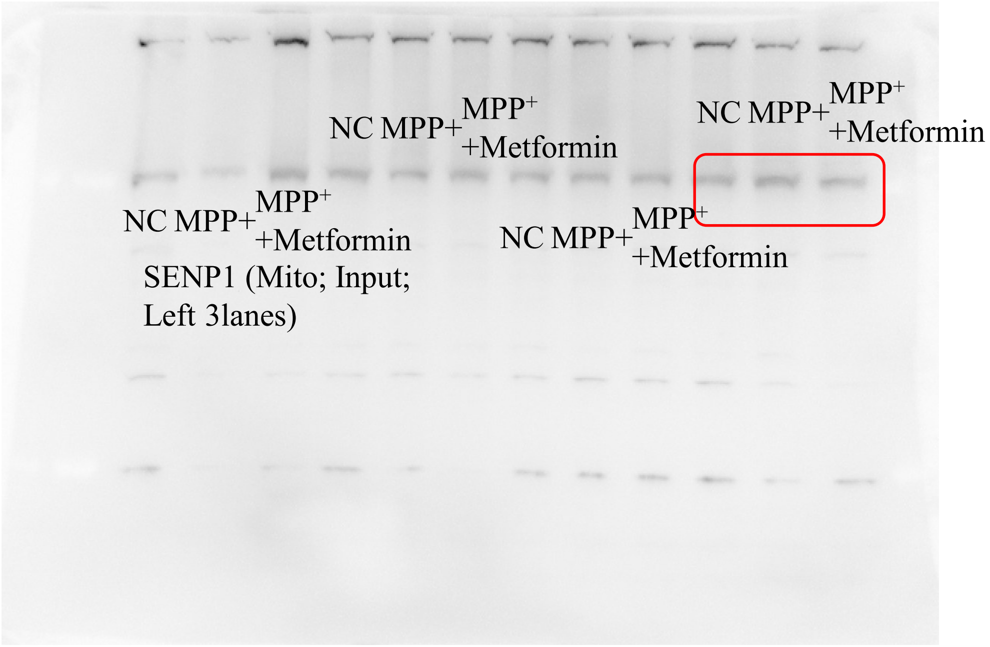

uncropped images

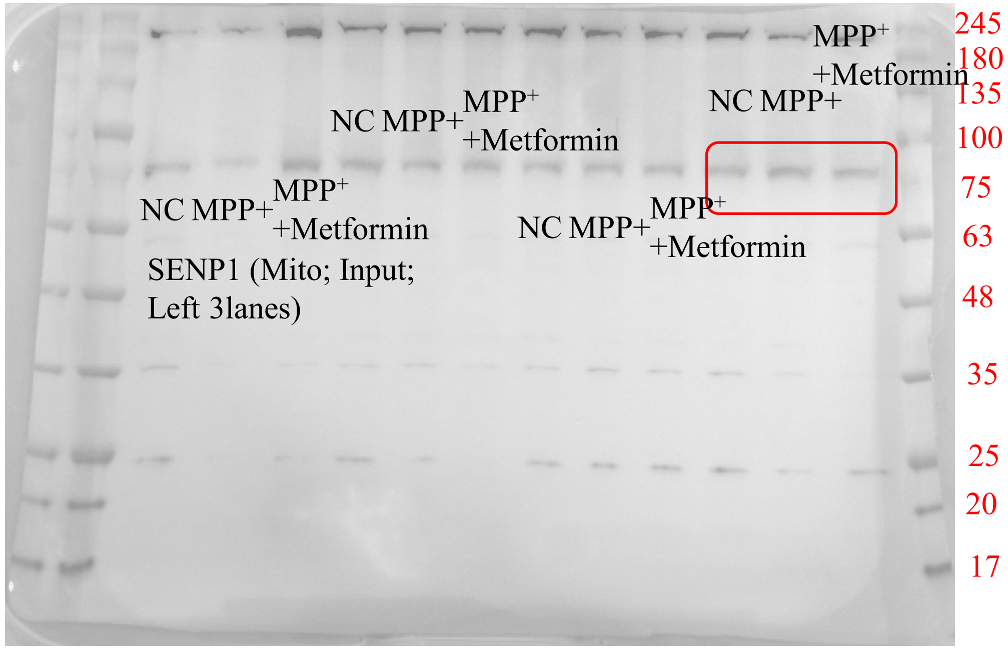

uncropped images (merged with marker)

SENP1 (Total; Input)

Figure 2B

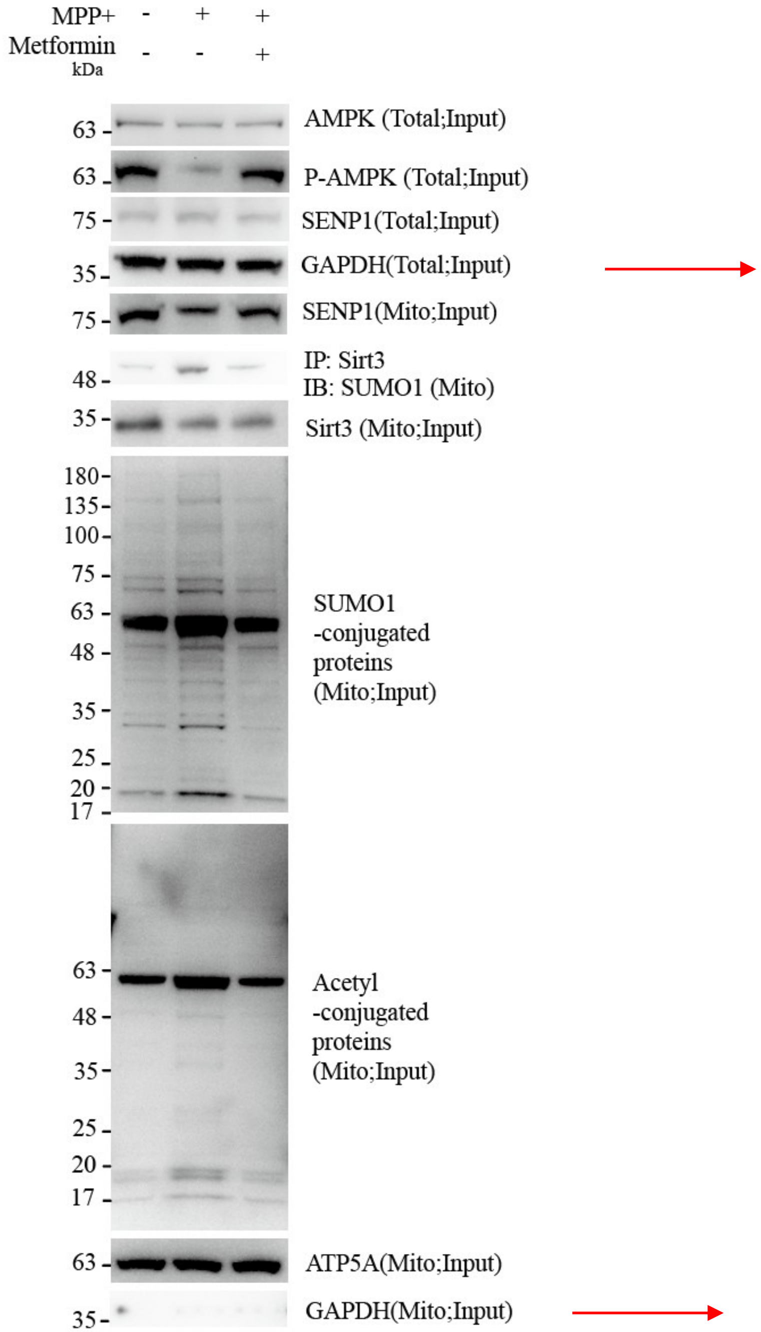

uncropped images

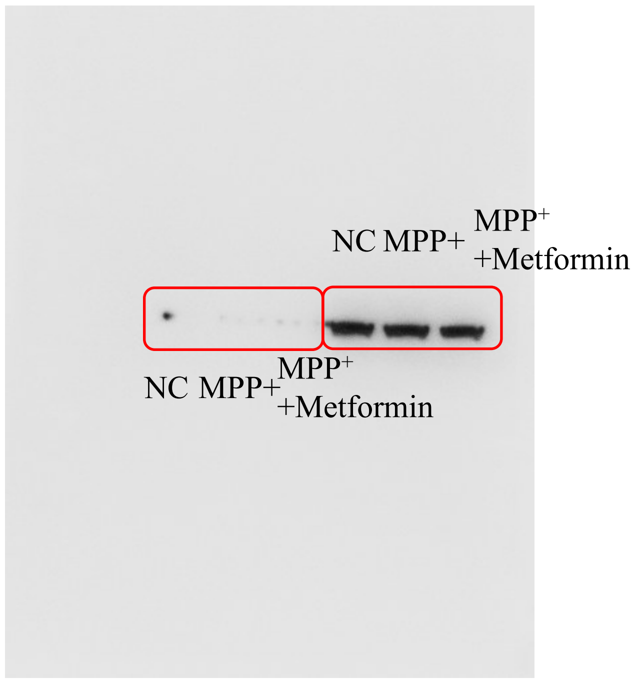

uncropped images
(merged with marker)

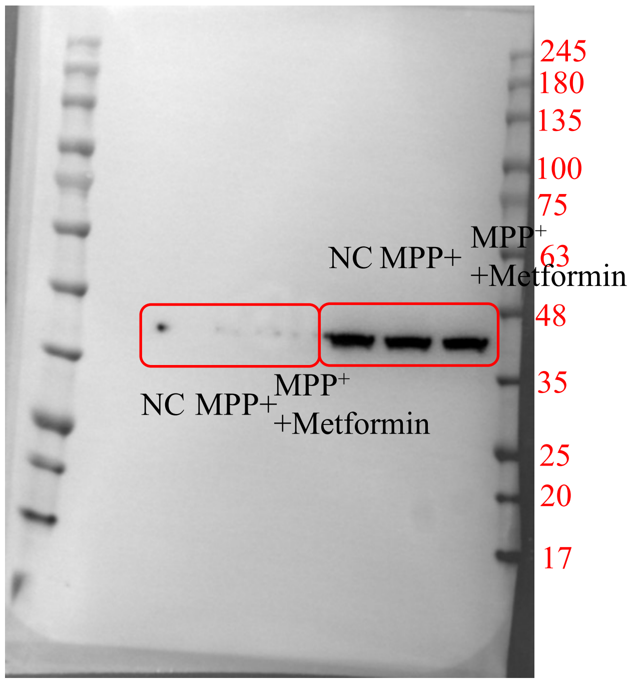

GAPDH (Mito; Input; Left 3 lanes) and
GAPDH (Total; Input; Right 3 lanes)

Figure 2B

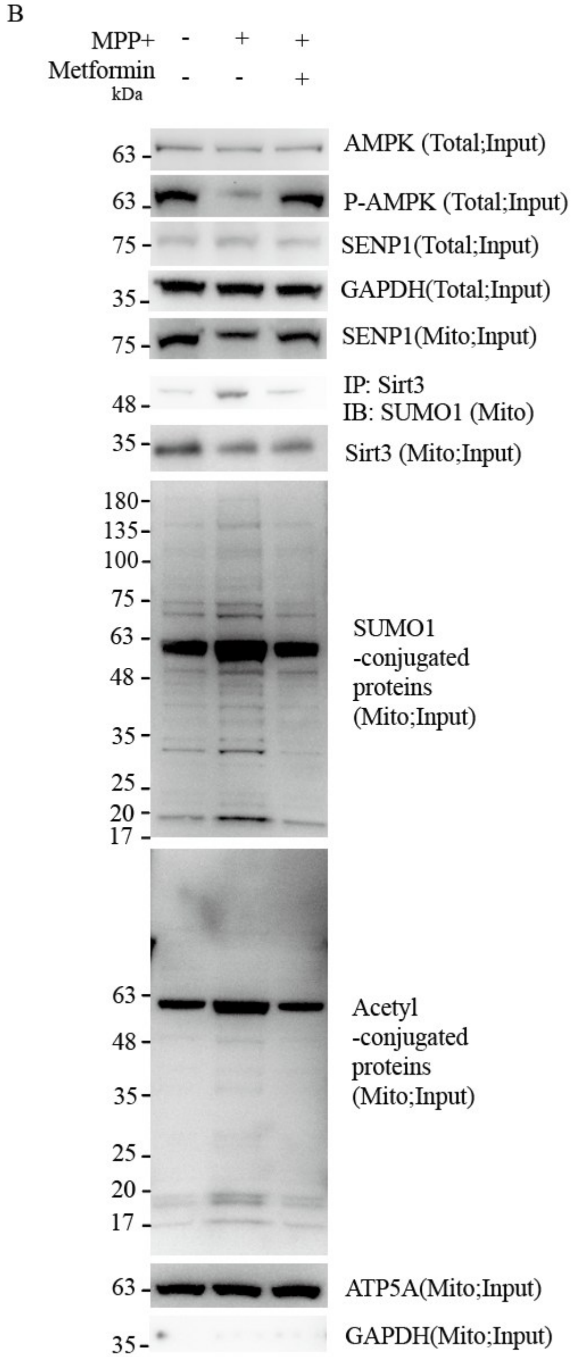

uncropped images

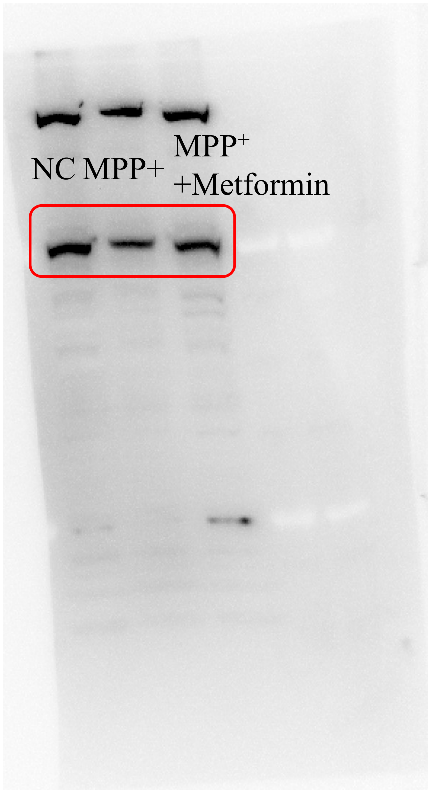

uncropped images
(merged with marker)

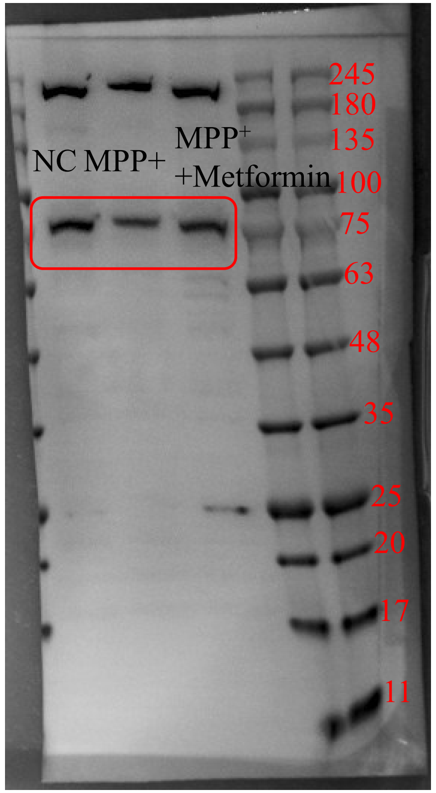

SENP1 (Mito; Input)

Figure 2B

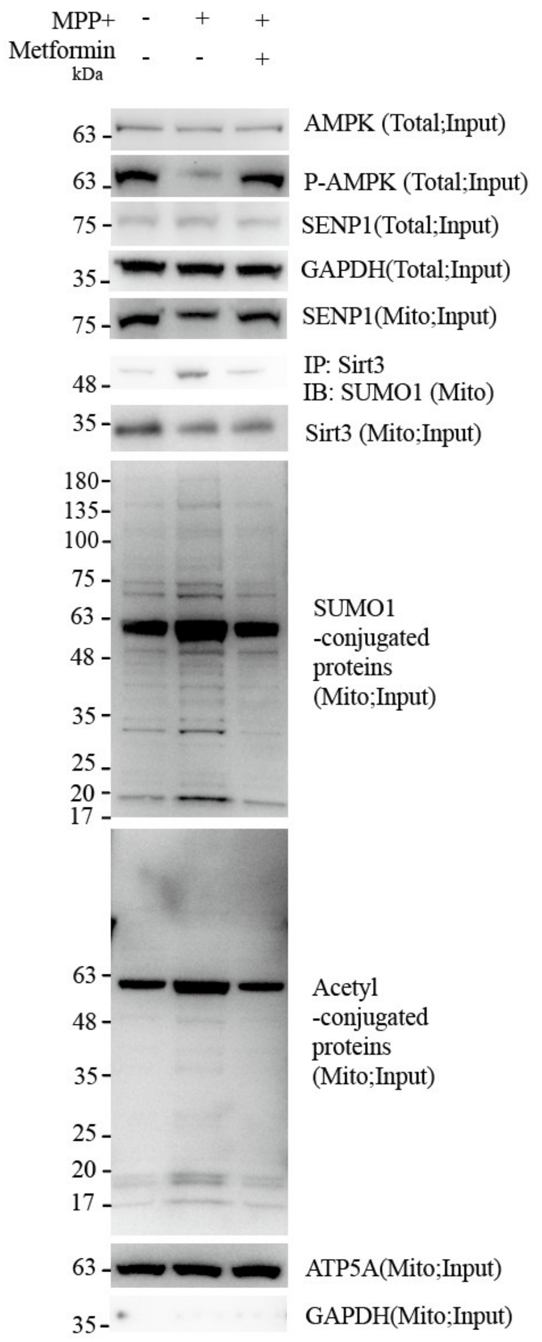

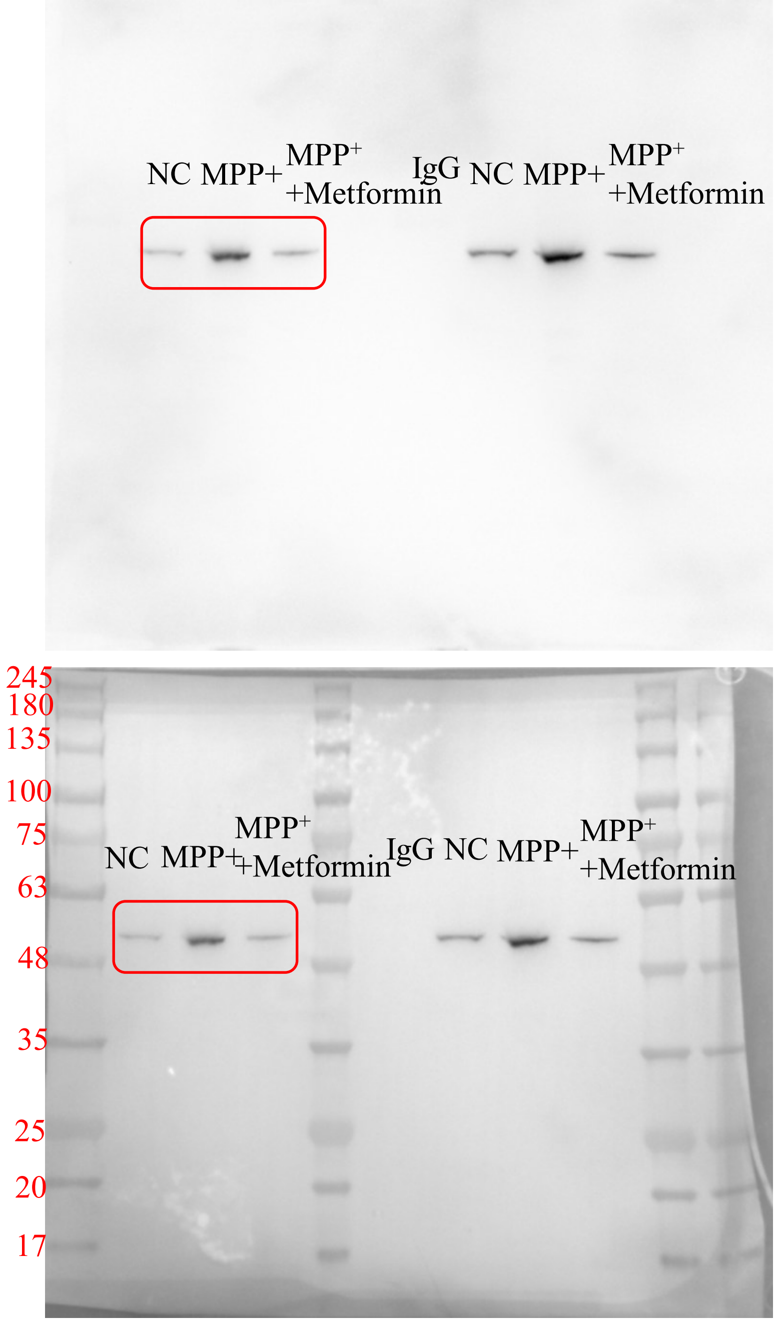

uncropped images

uncropped images (merged with marker)

IP Sirt3 IB SUMO1(Mito)

Figure 2B

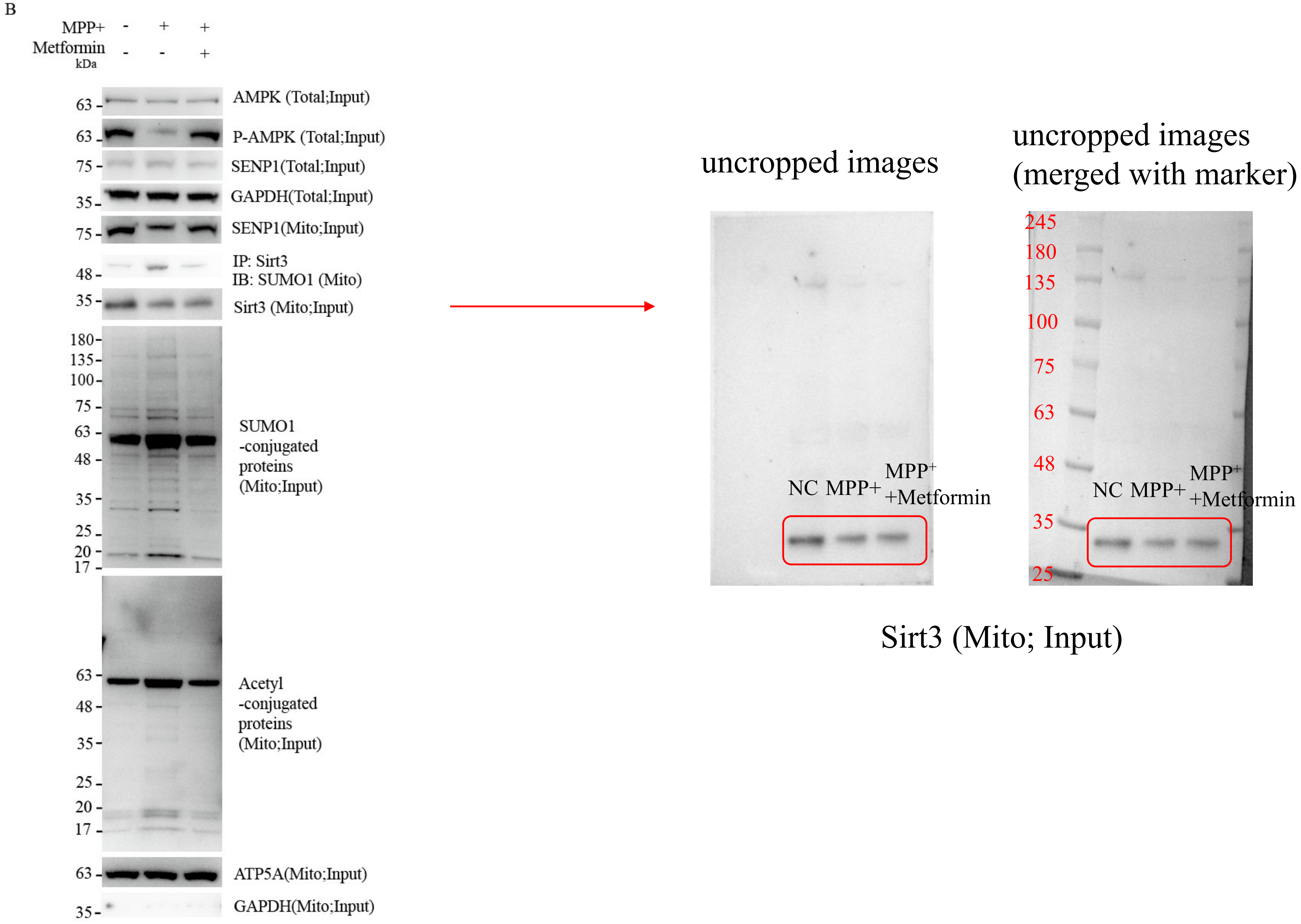

B
MPP+ - + +
Metformin - - +
kDa
63 AMPK (Total;Input)
63 P-AMPK (Total;Input)
75 SENP1(Total;Input)
35 GAPDH(Total;Input)
75 SENP1(Mito;Input)
48 IP: Sirt3
IB: SUMO1 (Mito)
35 Sirt3 (Mito;Input)
180 135 100 75 63 48 35 25 20 17
SUMO1
-conjugated
proteins
(Mito;Input)
63 48 35 25 20 17
Acetyl
-conjugated
proteins
(Mito;Input)
63 ATP5A(Mito;Input)
35 GAPDH(Mito;Input)
uncropped images
uncropped images
(merged with marker)
245 180 135 100 75 63 48 35 25
NC MPP+ MPP+
+Metformin
NC MPP+ MPP+
+Metformin
Sirt3 (Mito; Input)

Figure 2B

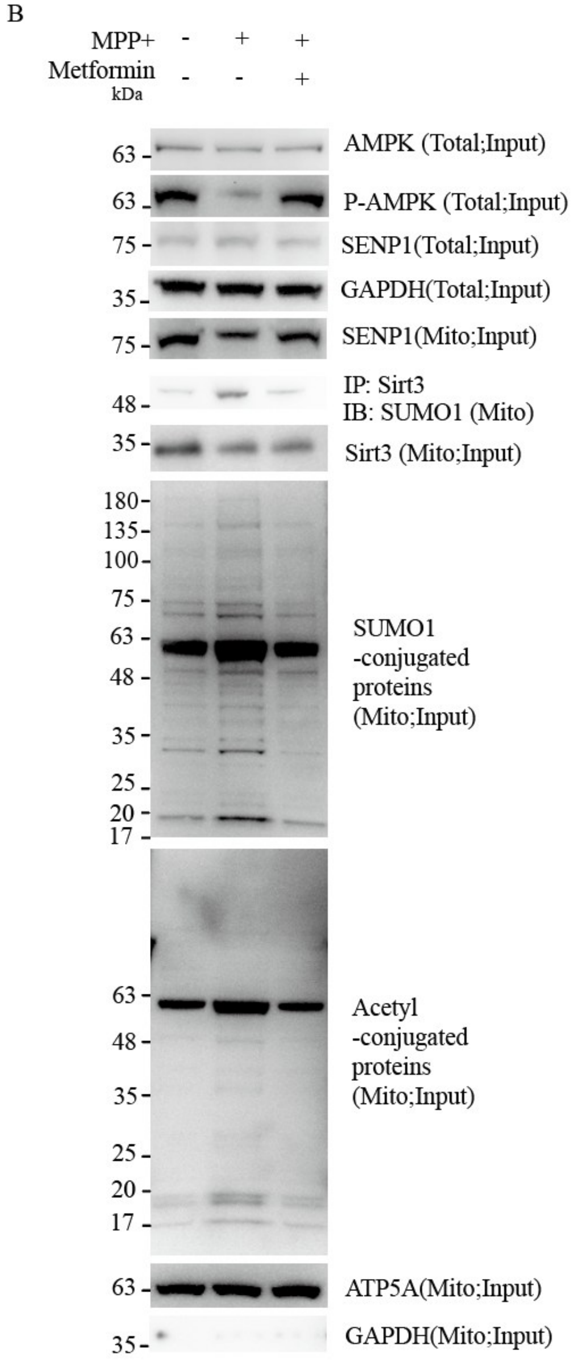

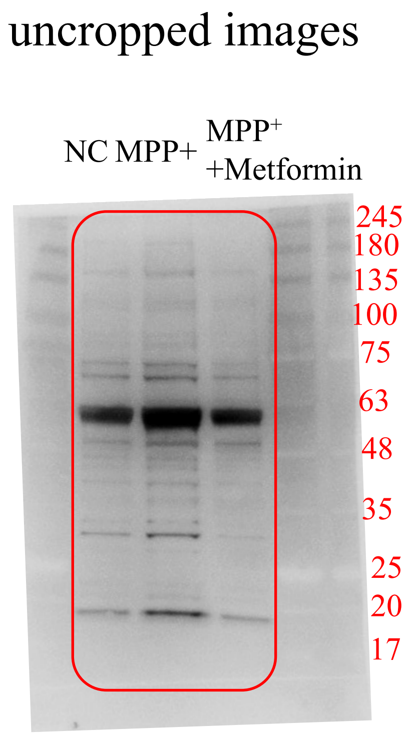

SUMO1-conjugated proteins (Mito; Input)

Figure 2B

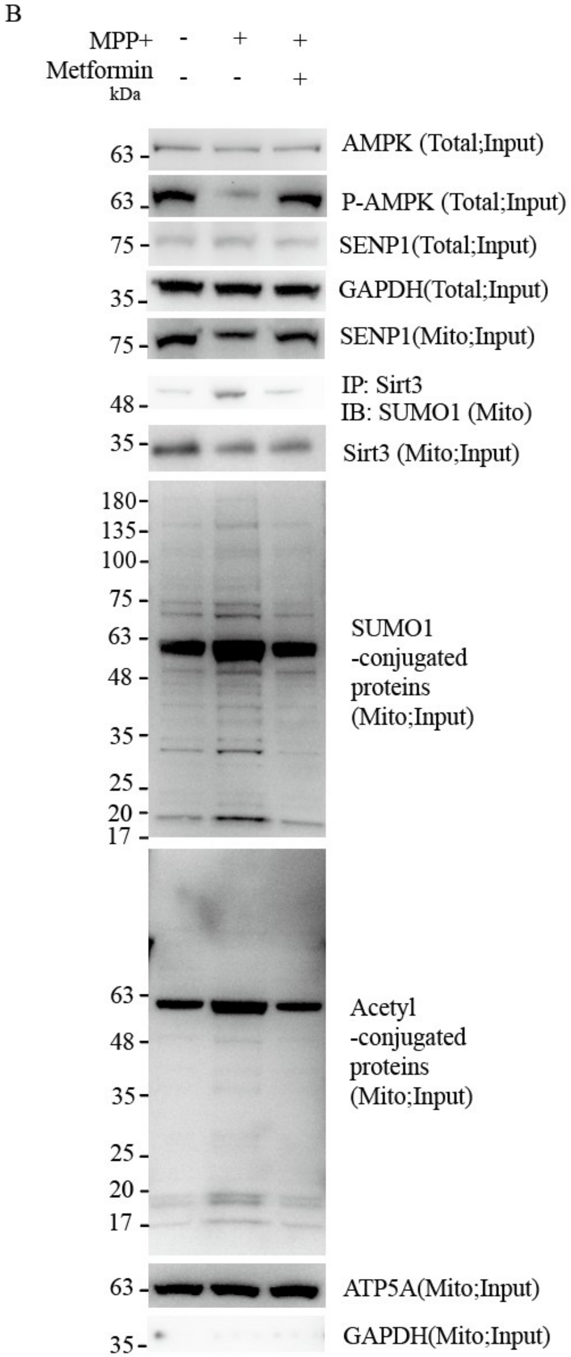

uncropped images

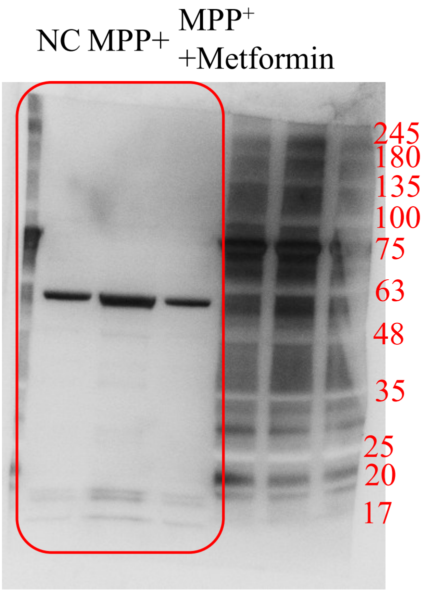

Acetyl-conjugated proteins
(Mito; Input)

Figure 2B

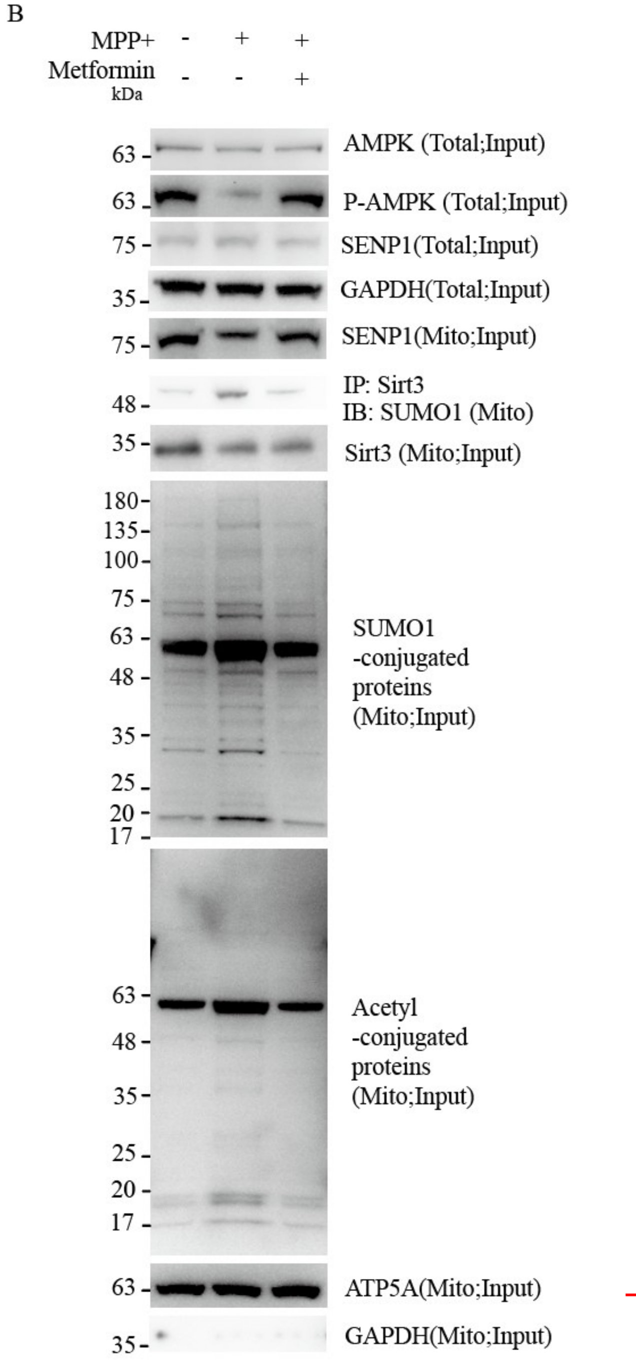

uncropped images

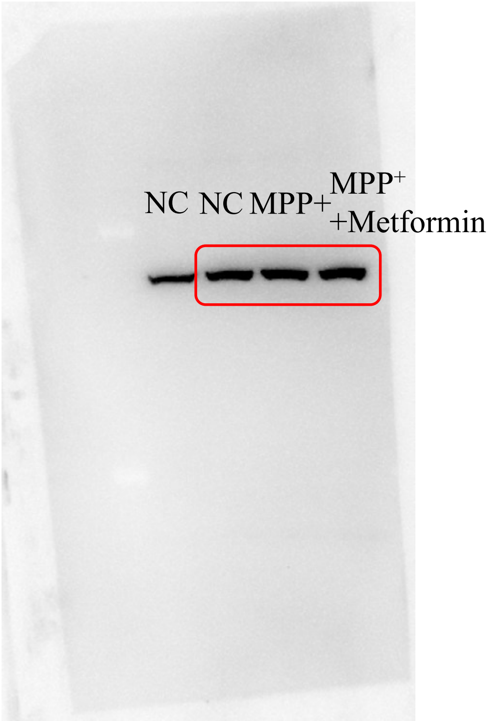

uncropped images (merged with marker)

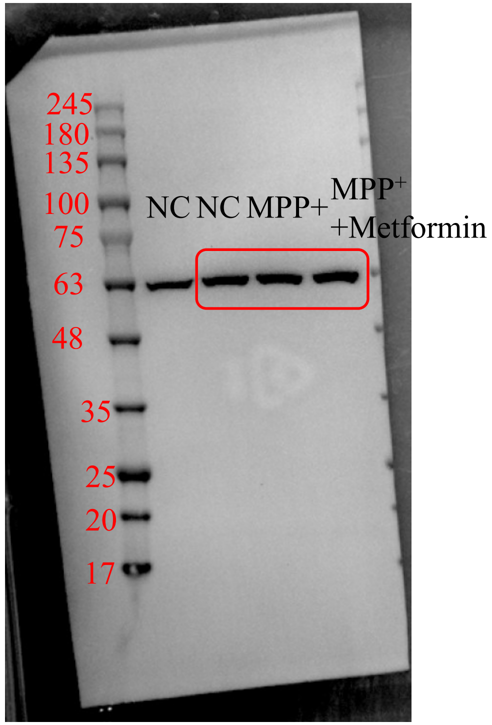

ATP5A (Mito; Input)

Figure 3A

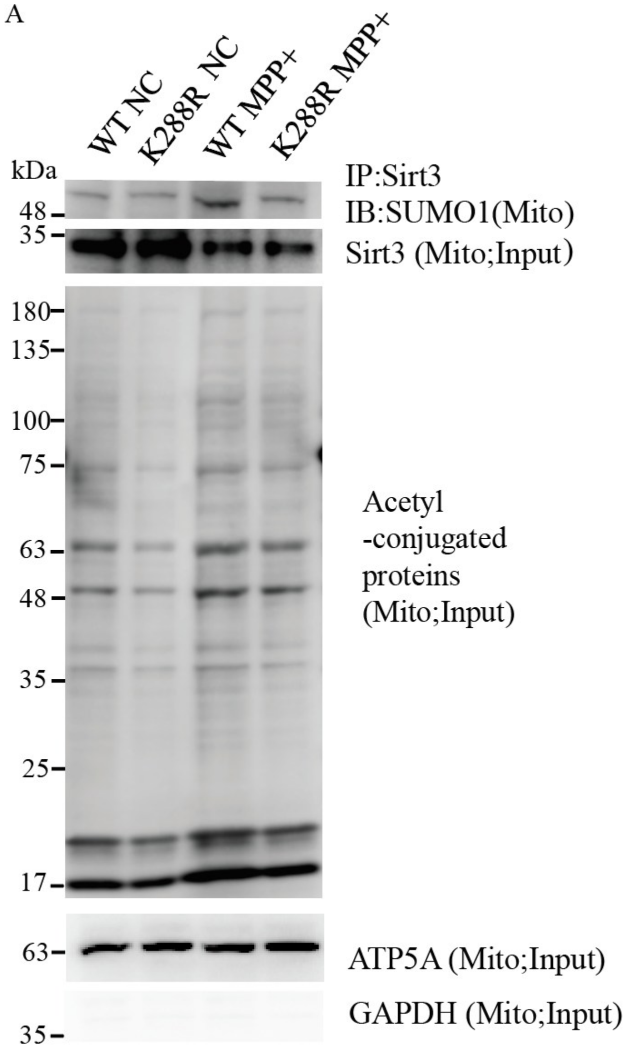

uncropped images

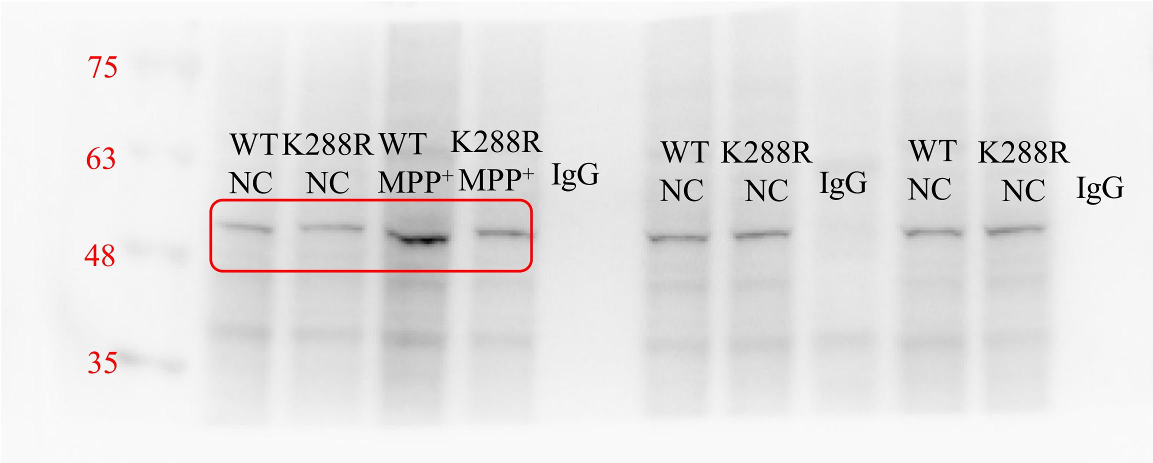

IP Sirt3 IB SUMO1(Mito)

Figure 3A

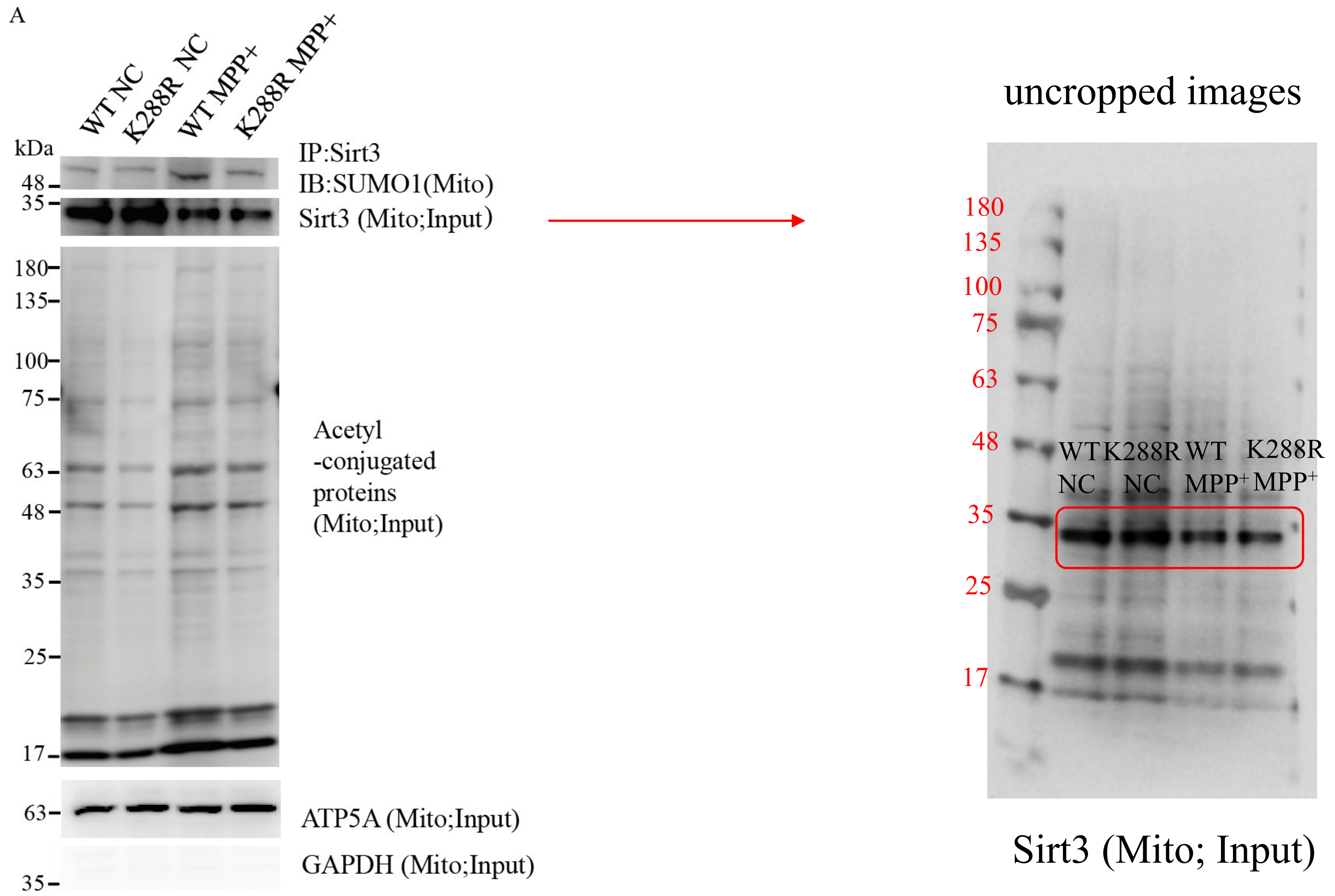
A
WT NC
K288R NC
WT MPP+
K288R MPP+
kDa
48
35
IP:Sirt3
IB:SUMO1(Mito)
Sirt3 (Mito;Input)
180
135
100
75
63
48
35
25
17
Acetyl
-conjugated
proteins
(Mito;Input)
63
ATP5A (Mito;Input)
GAPDH (Mito;Input)
35
uncropped images
180
135
100
75
63
48
35
25
17
WT NC
K288R NC
WT MPP+
K288R MPP+
Sirt3 (Mito; Input)

Figure 3A

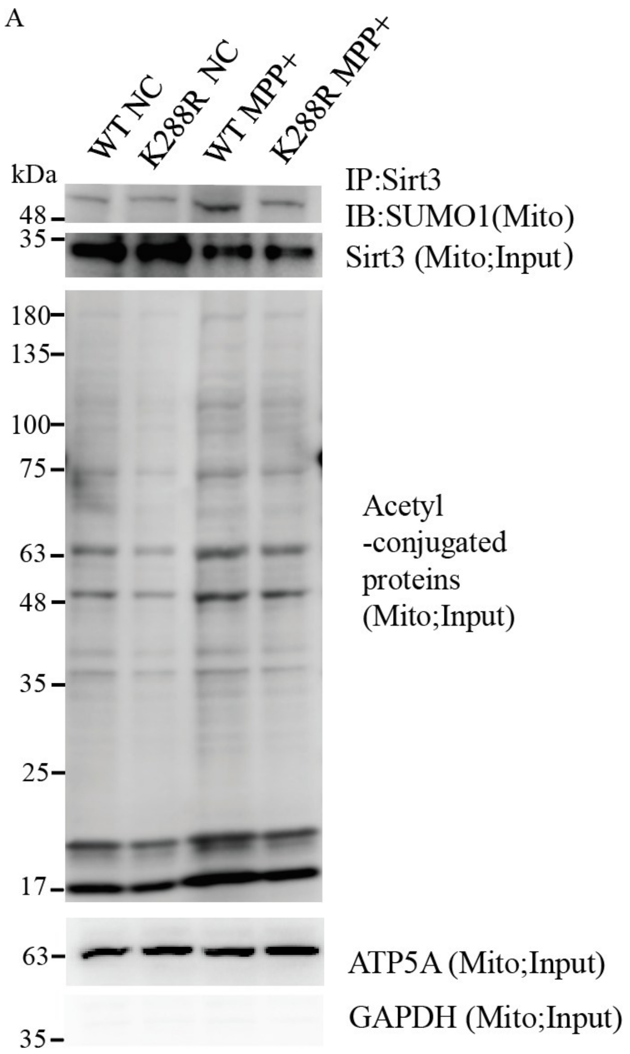

uncropped images

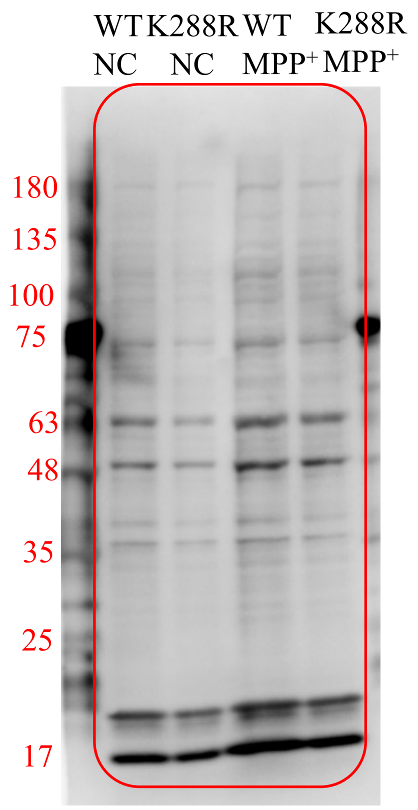

Acetyl-conjugated proteins (Mito; Input)

Figure 3A

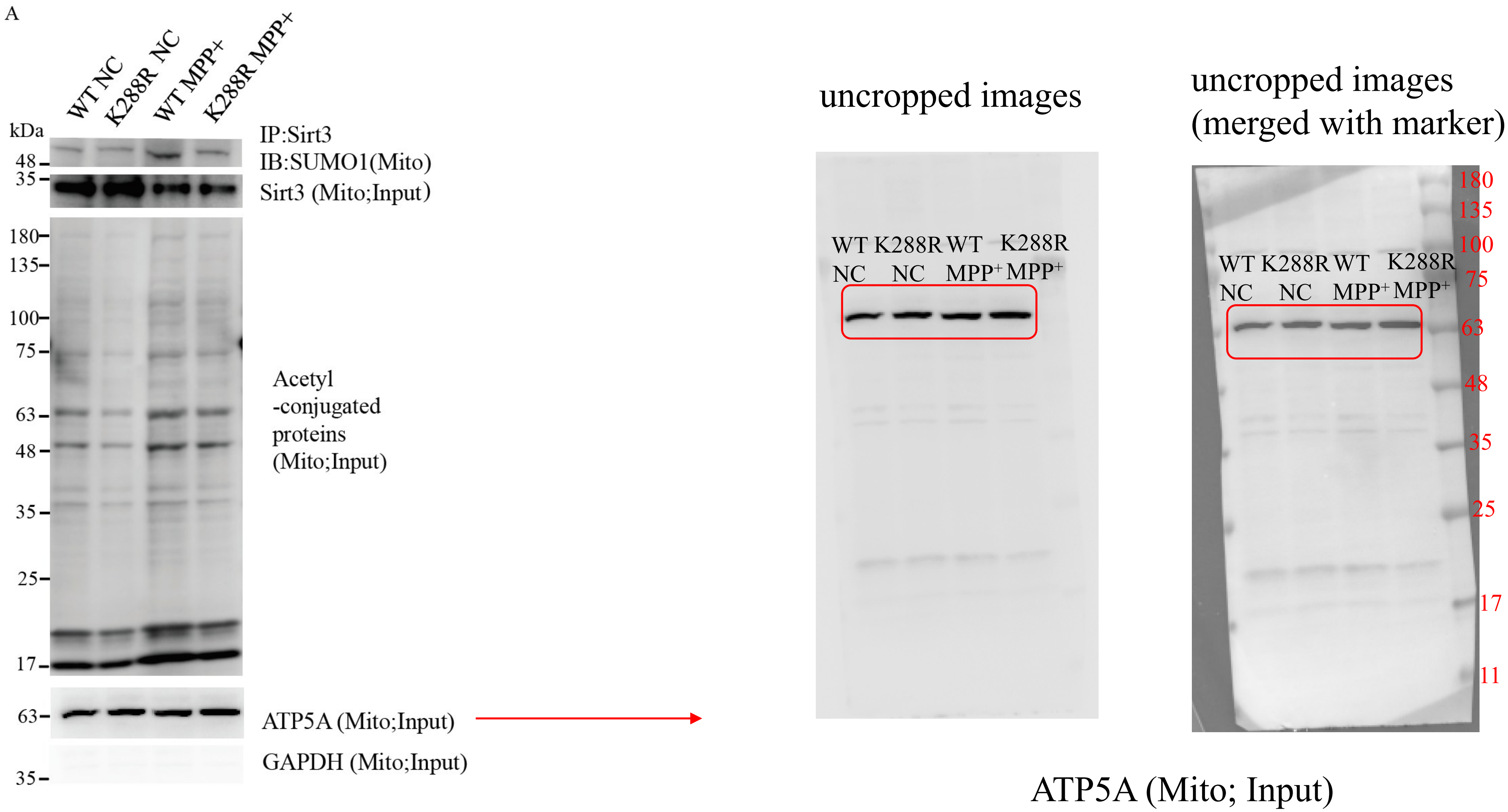
A
WT NC
K288R NC
WT MPP+
K288R MPP+
kDa
48
35
IP:Sirt3
IB:SUMO1(Mito)
Sirt3 (Mito;Input)
180
135
100
75
63
48
35
25
17
Acetyl
-conjugated
proteins
(Mito;Input)
63
ATP5A (Mito;Input)
GAPDH (Mito;Input)
35
uncropped images
WT NC
K288R NC
WT MPP+
K288R MPP+
uncropped images
(merged with marker)
180
135
100
75
63
48
35
25
17
11
ATP5A (Mito; Input)

Figure 3A

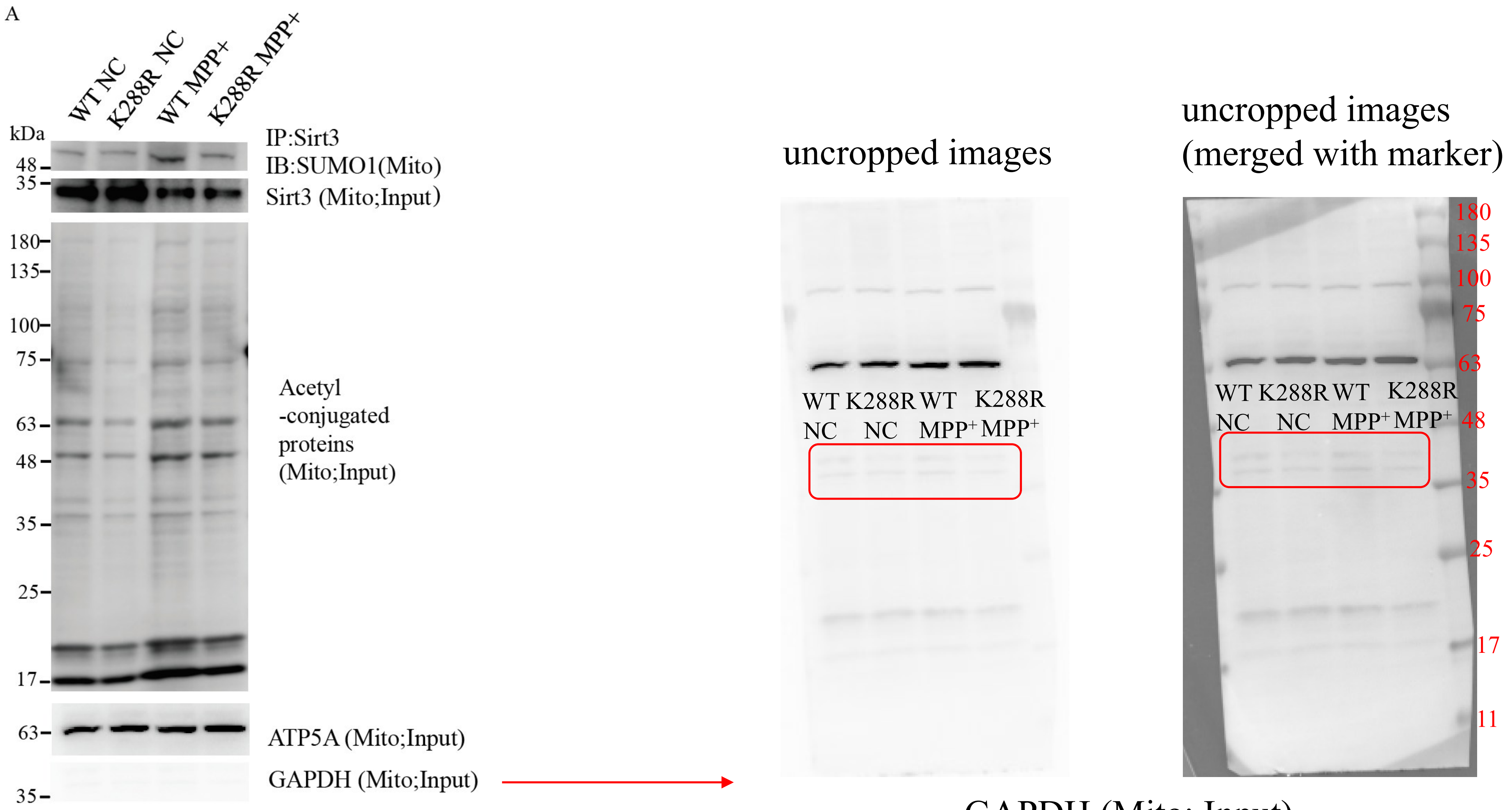

GAPDH (Mito; Input)

Figure 3E

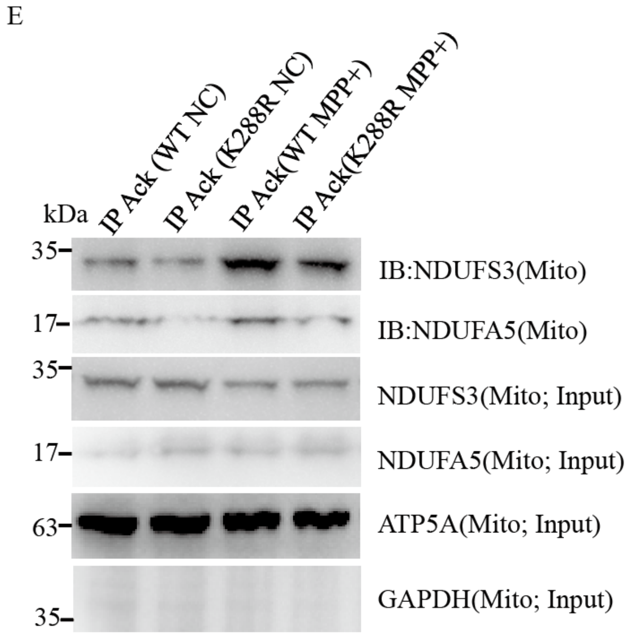

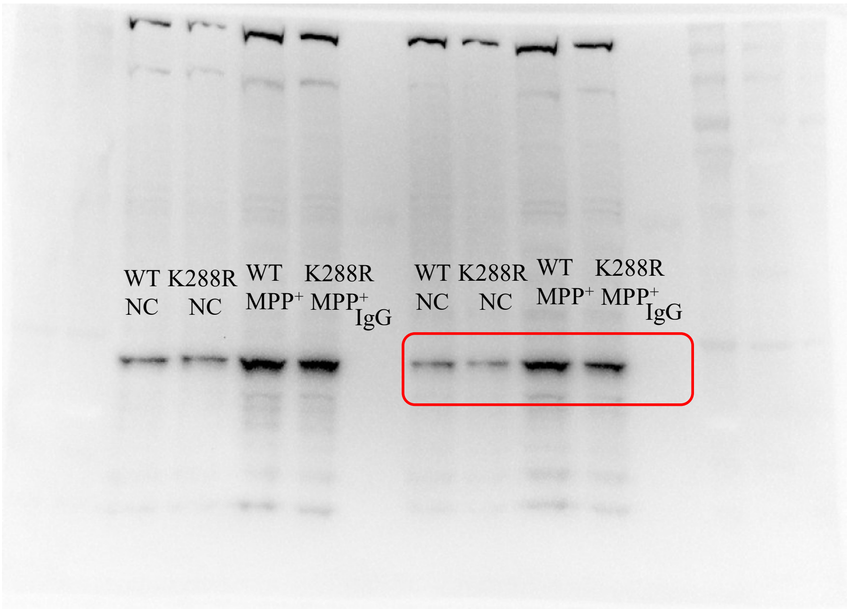

uncropped images

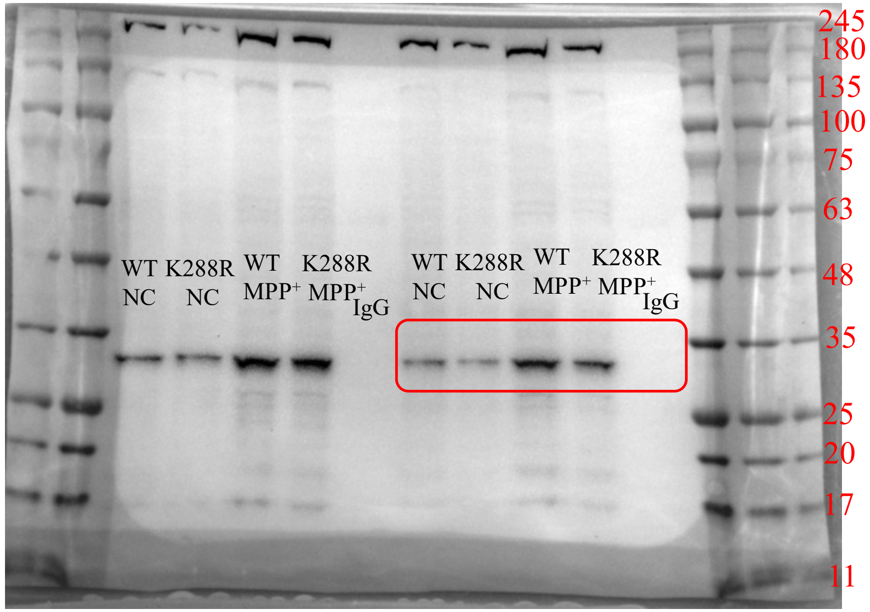

uncropped images (merged with marker)

NDUFS3 (Mito)

Figure 3E

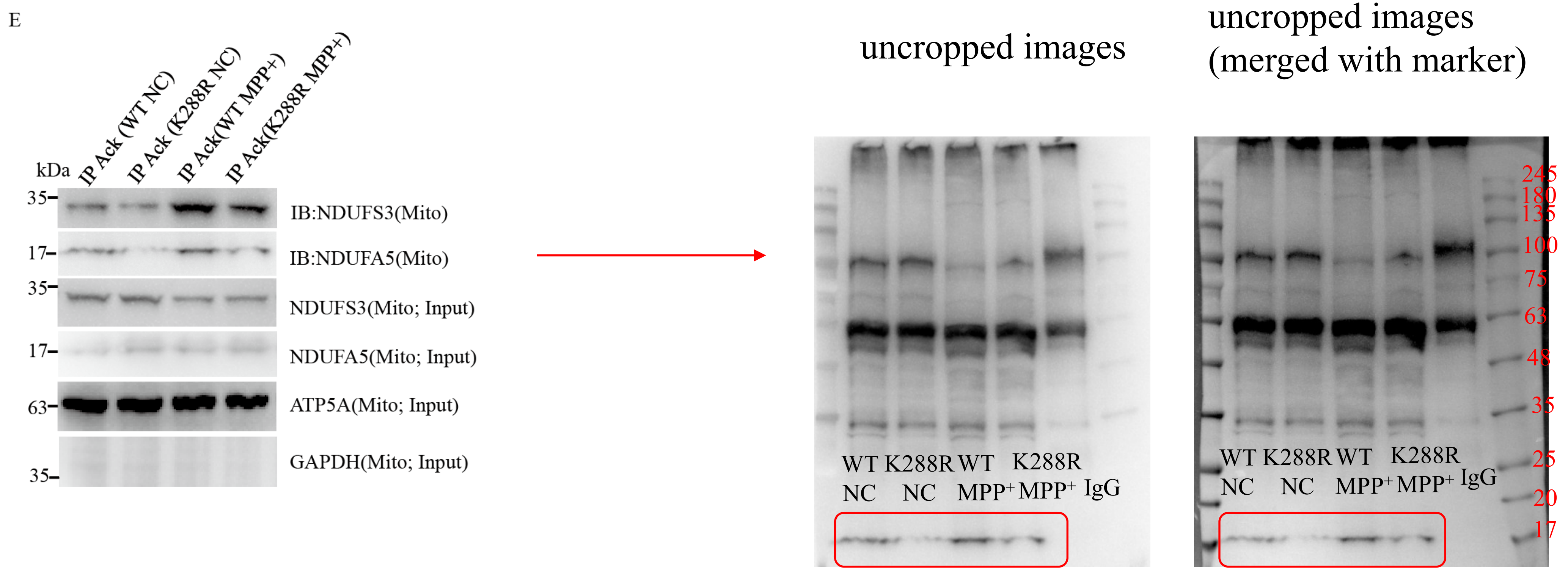

NDUFA5 (Mito)

Figure 3E

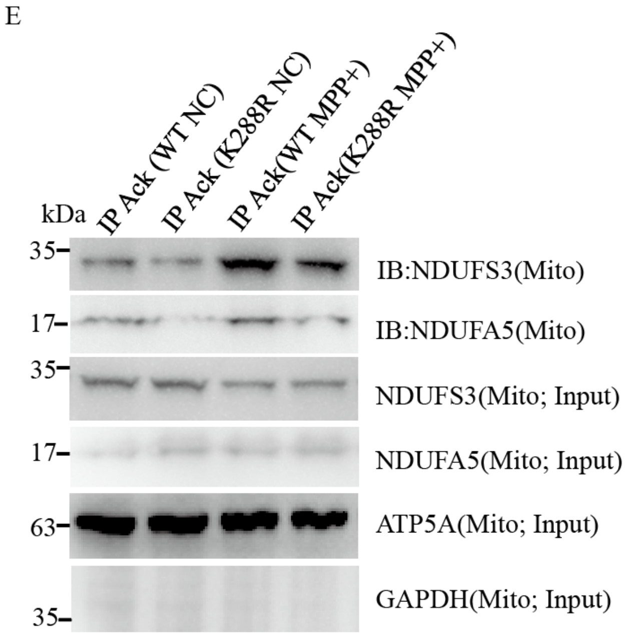

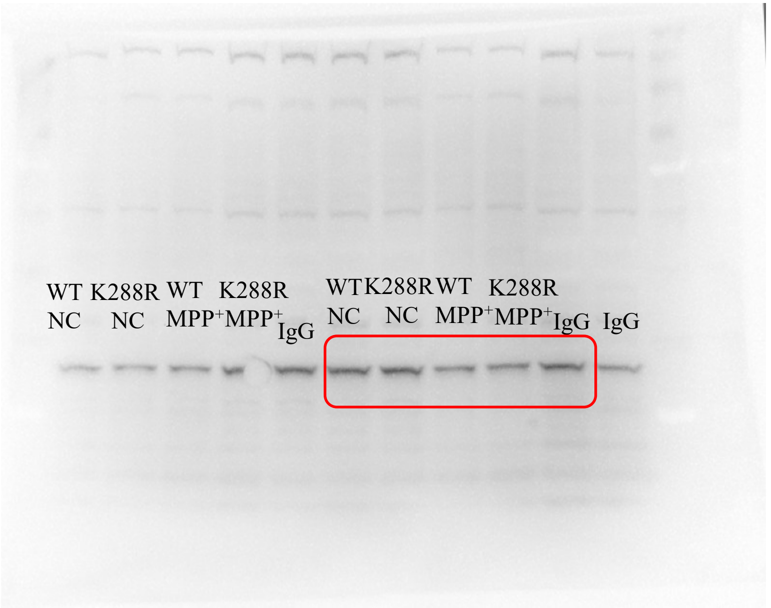

uncropped images

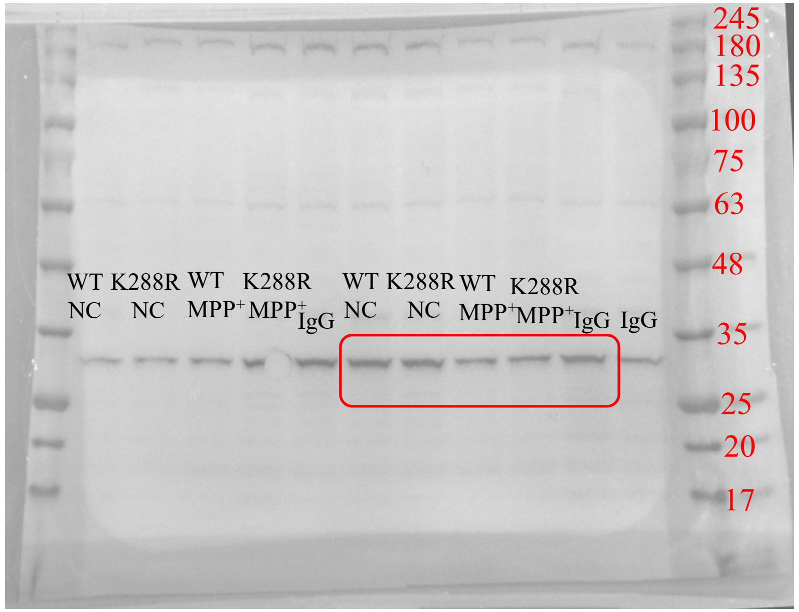

uncropped images (merged with marker)

NDUFS3 (Mito; Input)

Figure 3E

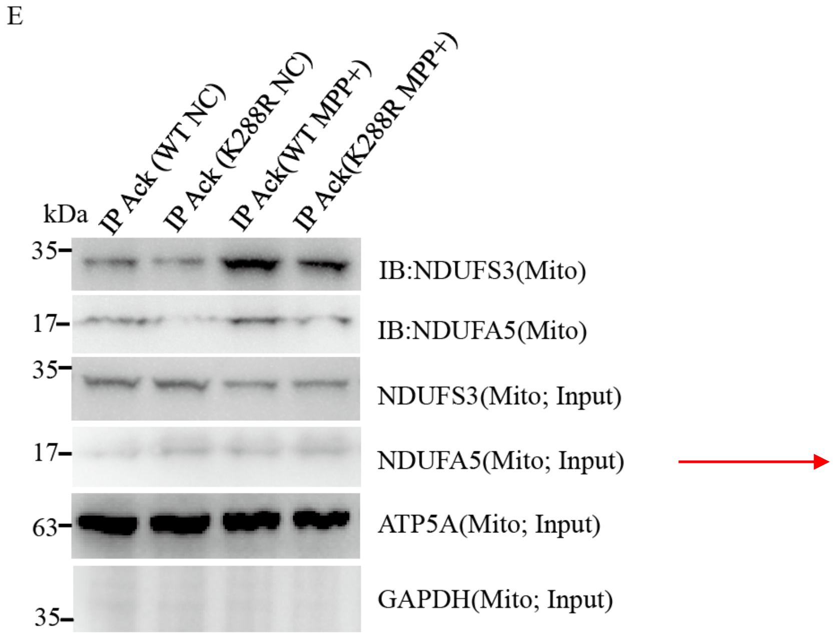

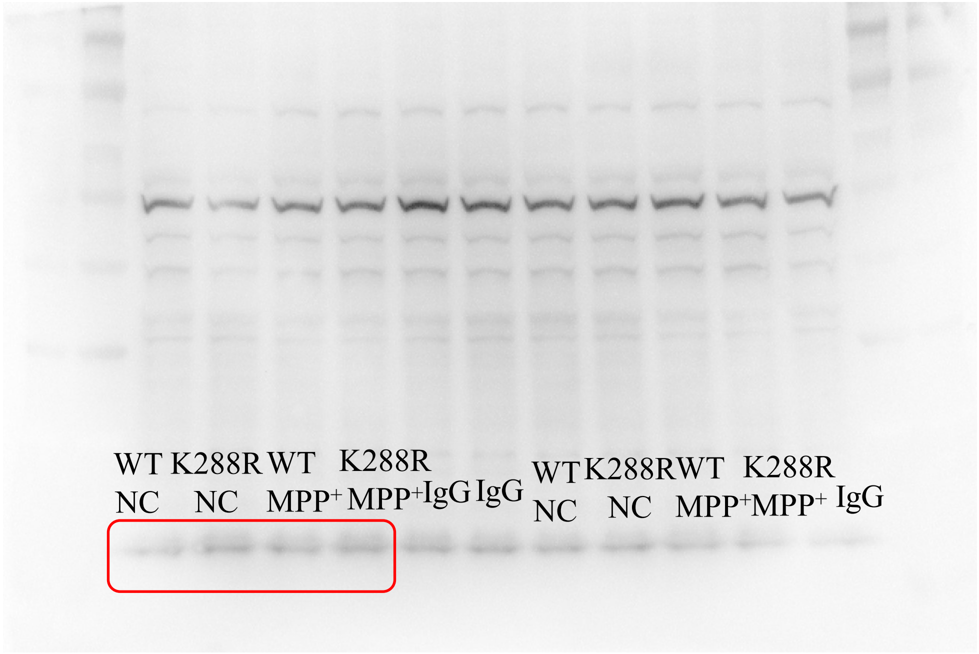

uncropped images

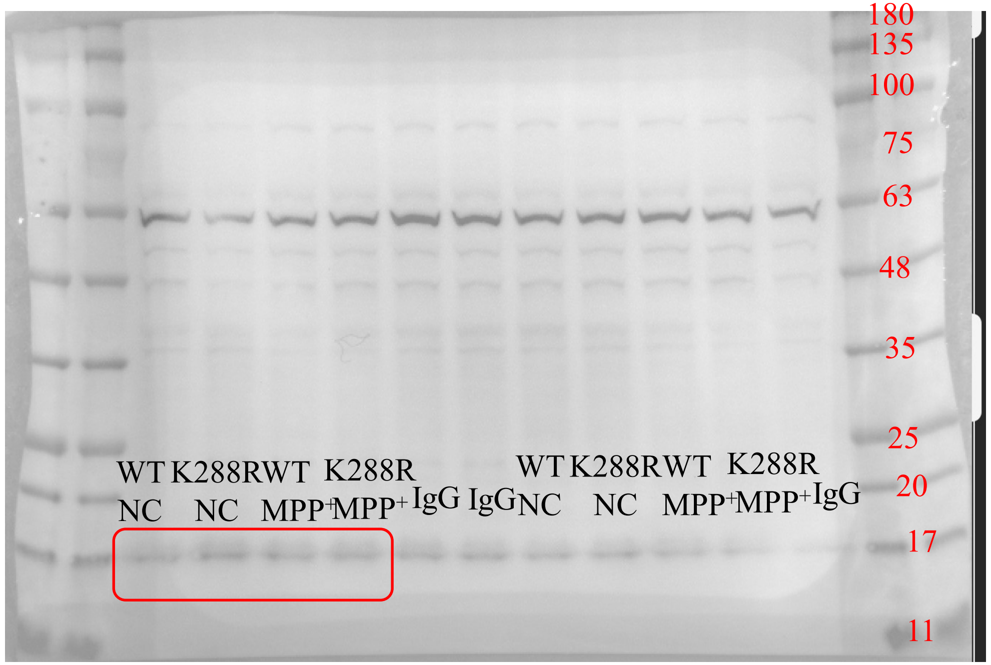

uncropped images (merged with marker)

NDUFA5 (Mito; Input)

Figure 3E

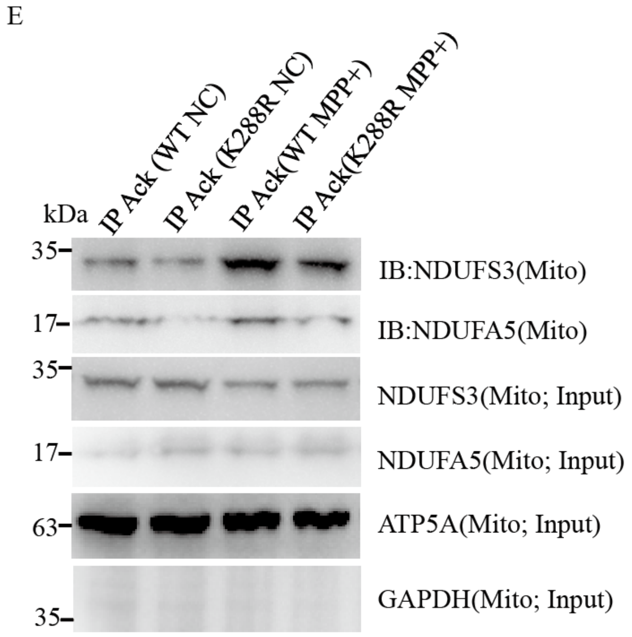

uncropped images

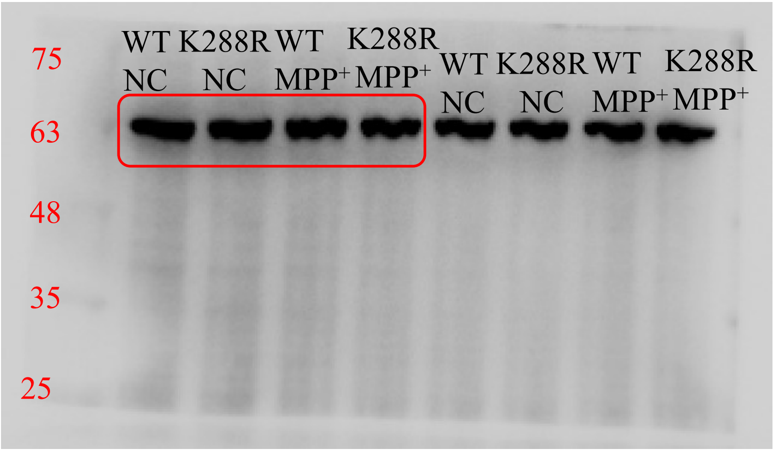

ATP5A (Mito; Input)

Figure 3E

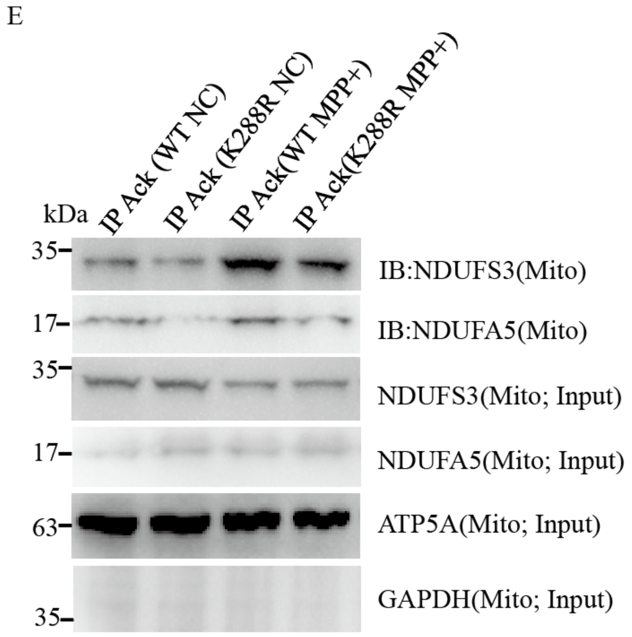

uncropped images

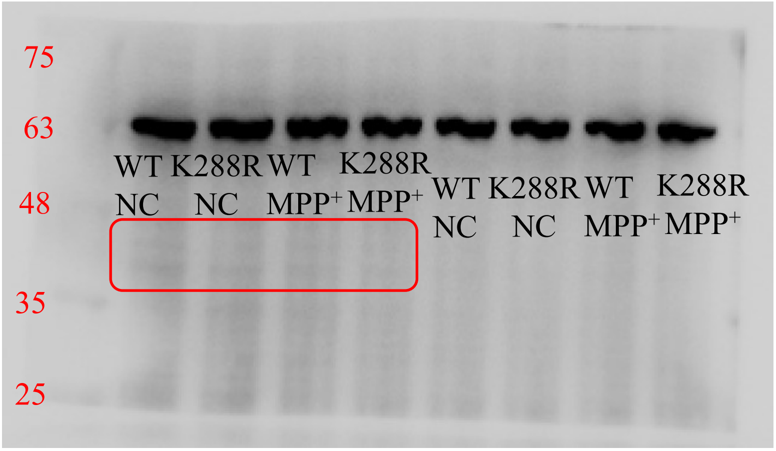

GAPDH (Mito; Input)

Figure 5G

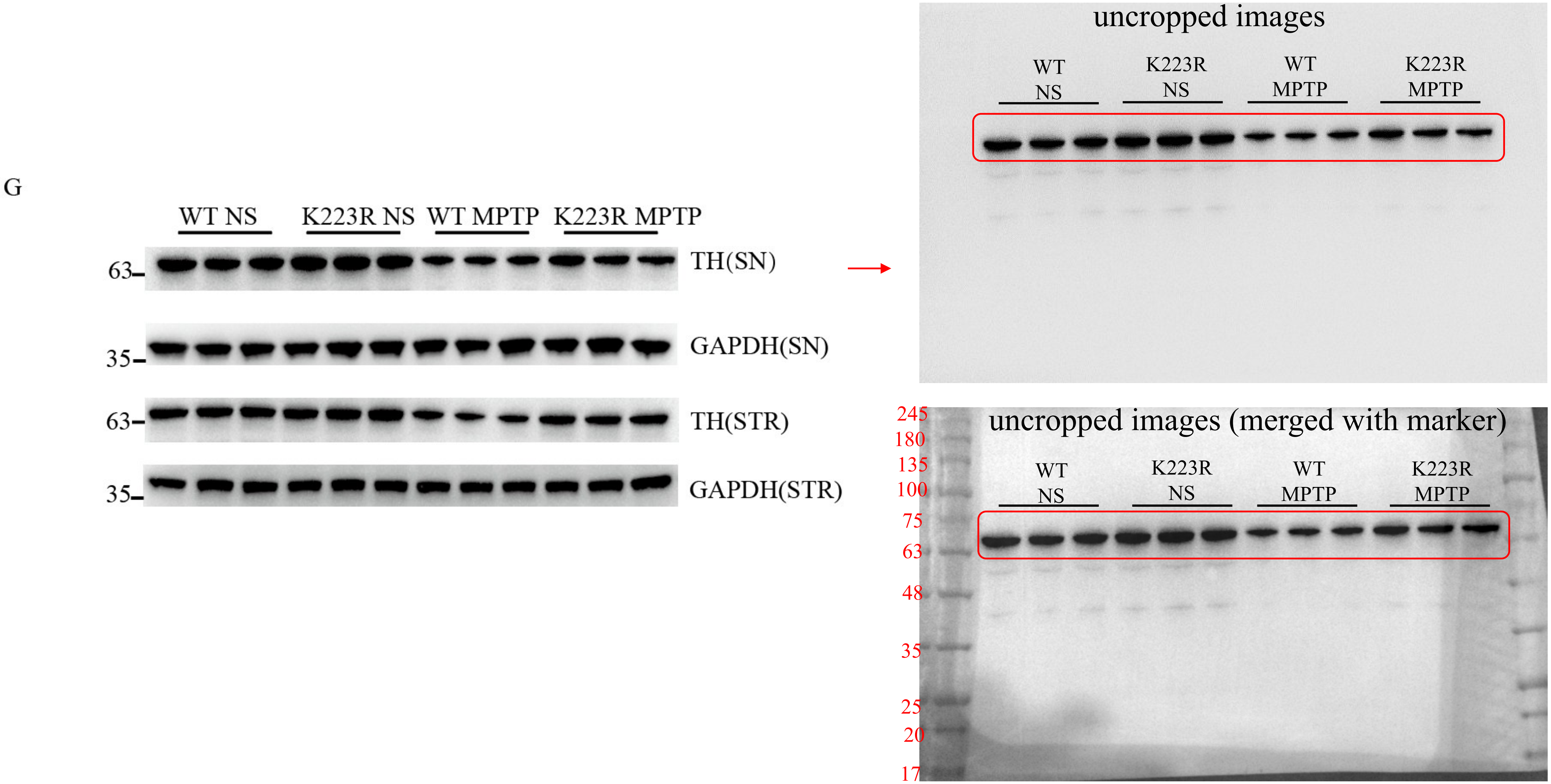

TH(SN)

Figure 5G

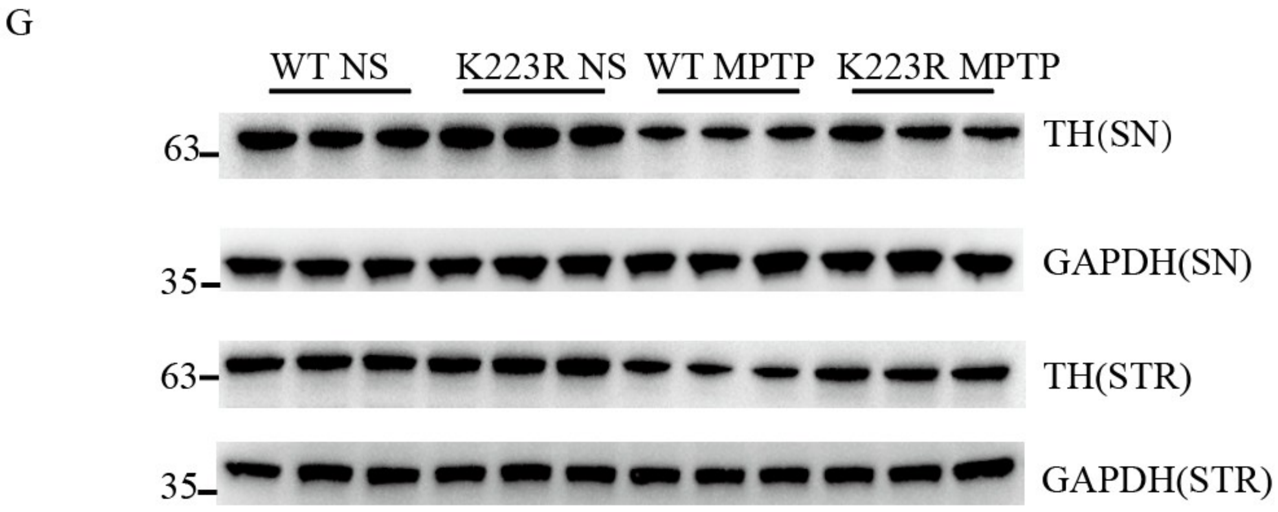

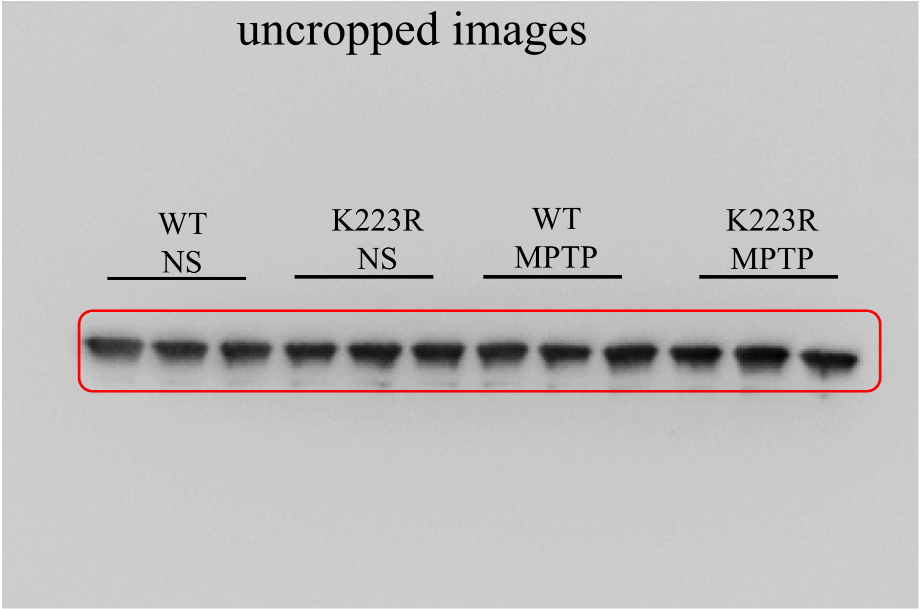

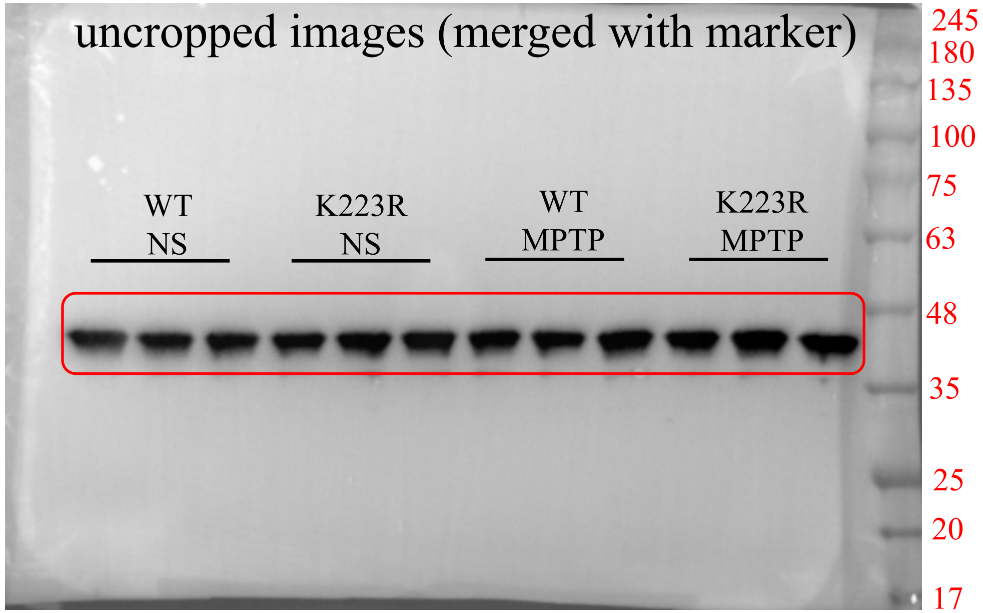

GAPDH(SN)

Figure 5G

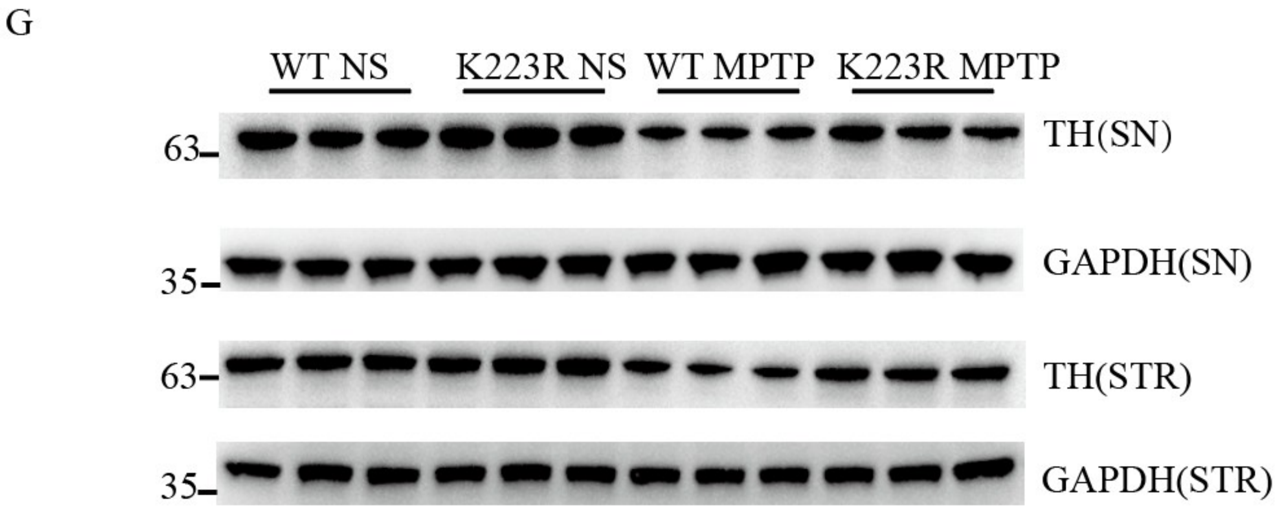

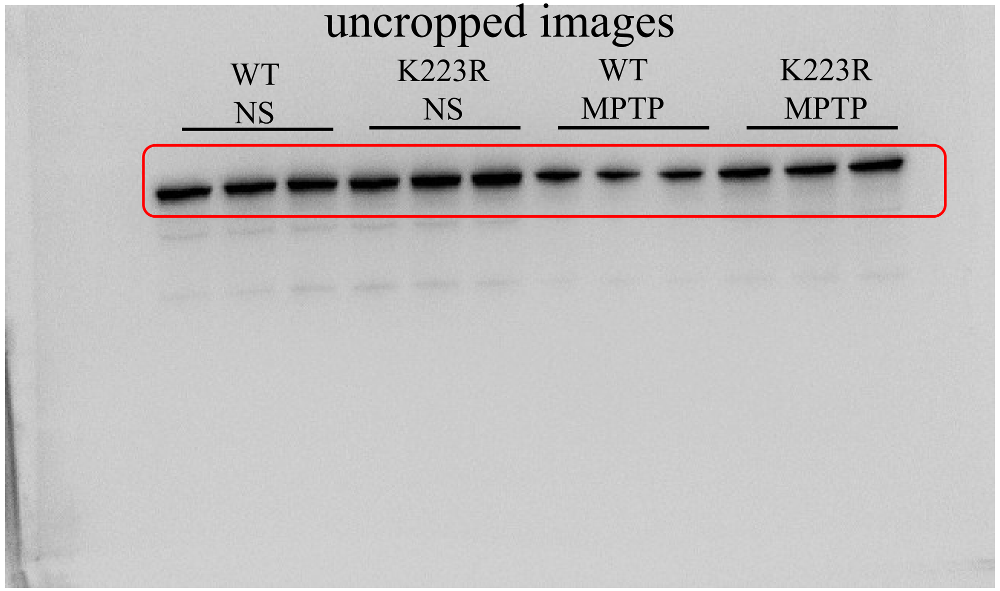

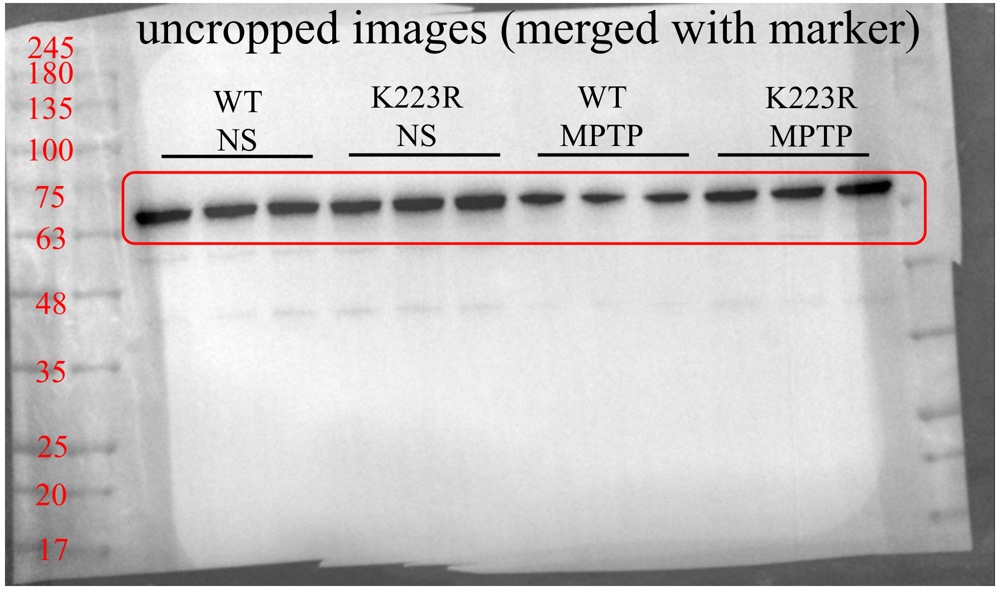

TH(STR)

Figure 5G

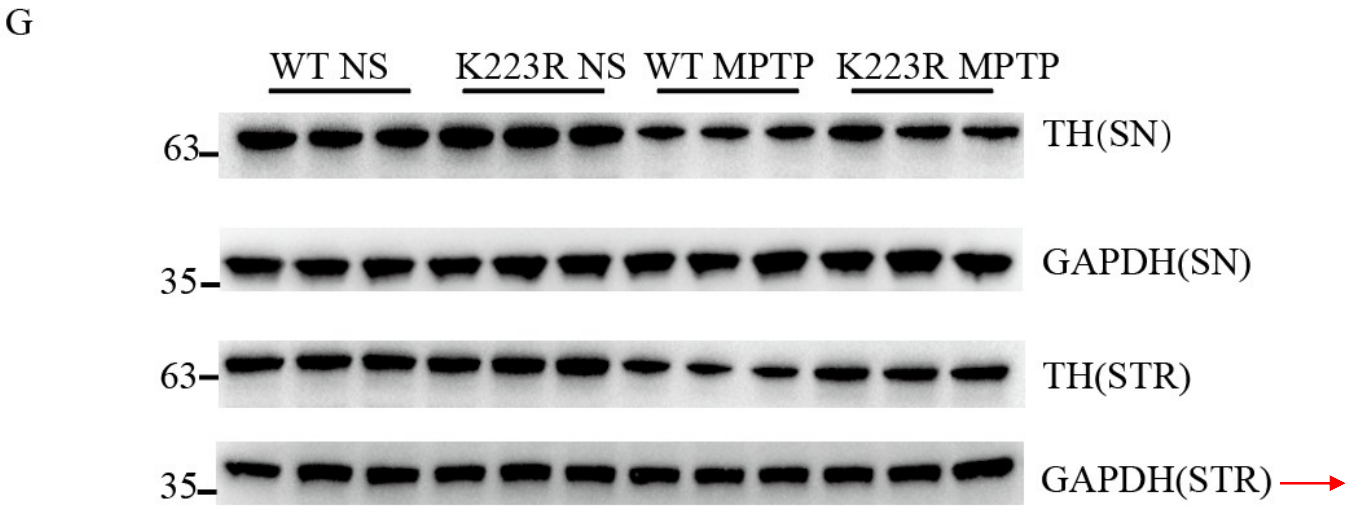

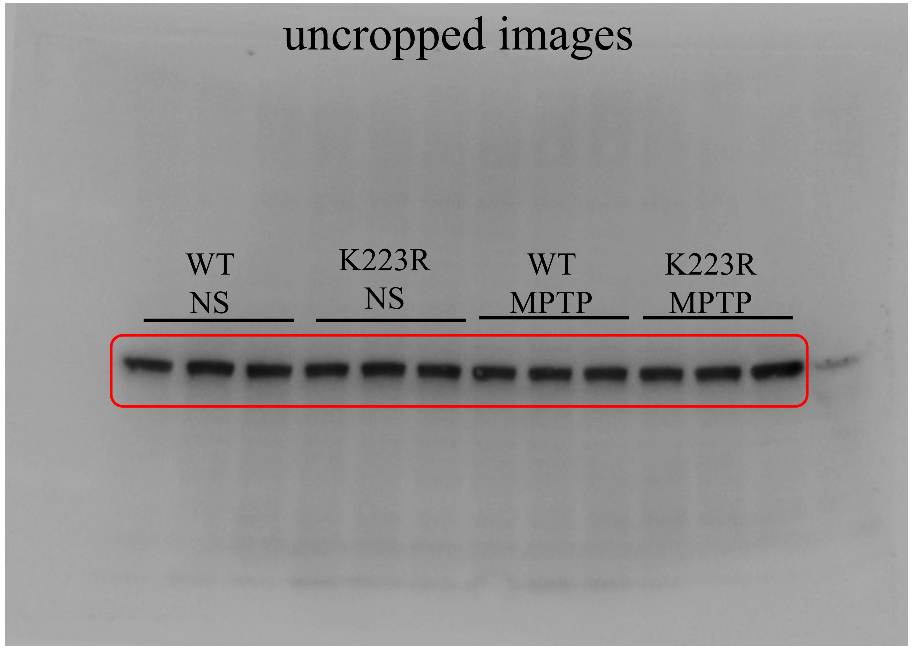

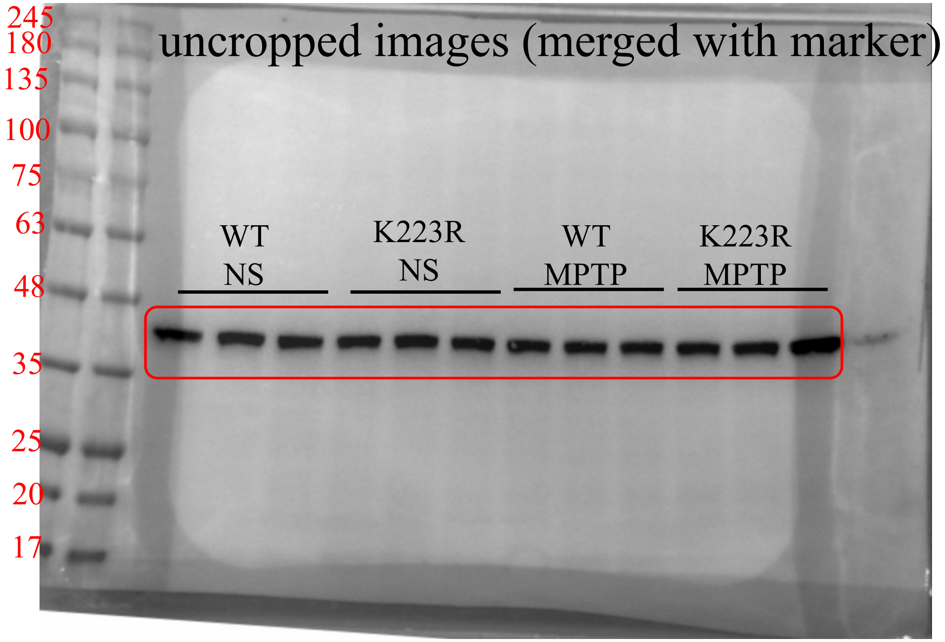

GAPDH(STR)

Additional Figure 1

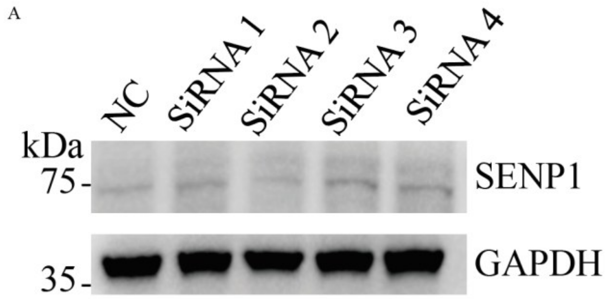

uncropped images

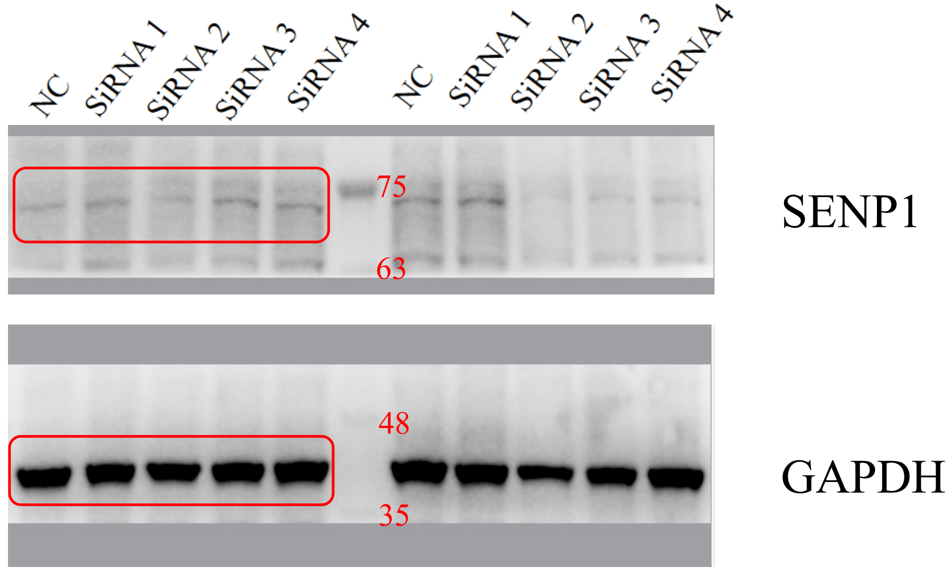

Supplement: Supplementary file 2 — Additional file 2: Uncropped Western blot images. [file 40035_2025_489_MOESM2_ESM.pdf]
